# Effectiveness of Text Message Interventions for Weight Management in Adolescents: Systematic Review

**DOI:** 10.2196/15849

**Published:** 2020-05-26

**Authors:** Stephanie Ruth Partridge, Rebecca Raeside, Anna Singleton, Karice Hyun, Julie Redfern

**Affiliations:** 1 The University of Sydney Westmead Applied Research Centre Faculty of Medicine and Health Sydney Australia; 2 The University of Sydney Prevention Research Collaboration, Charles Perkins Centre Sydney School Public Health Sydney Australia; 3 The George Institute for Global Health Sydney Australia

**Keywords:** adolescent, text message, obesity, overweight, prevention, mHealth

## Abstract

**Background:**

The incidence of obesity among adolescents is increasing. Text messages are a primary communication form for adolescents and potentially a scalable strategy for delivering population health interventions.

**Objective:**

This study aimed to determine the effectiveness of text message interventions in reducing BMI in adolescents and describe characteristics that are common to effective interventions.

**Methods:**

This systematic review included randomized controlled trials of text message lifestyle interventions involving adolescents aged 10 to 19 years with outcomes focused on obesity prevention or management. Primary outcome was objective or self-report change in BMI.

**Results:**

In total, 4362 records were identified, and 215 full-text articles were assessed for eligibility. A total of 8 unique studies were identified, including 767 participants, mean age 14.3 (SD 0.9) years, BMI 29.7 (SD 1.6) kg/m2 and 53.1% (407/767) female (31/101, 30.7%-172/172, 100.0%). All interventions were multicomponent. The median active intervention period was 4.5 months. During the active and extended intervention phases, text messages accounted for >50% (8 studies) and >85% (3 studies) of contact points, respectively. Text messages were heterogeneous, with a median of 1.5 text messages sent per week (range: 1-21). A total of 4 studies utilized two-way text message communication with health professionals Of the 8 studies, 7 demonstrated reductions in BMI or BMI z-score in the intervention group compared with the control at the end of the final follow-up. The effect was only statistically significant in 1 study at 6 months. Over 6 months, reductions in BMI (kg/m2) ranged from 1.3% to 4.5% and BMI z-score ranged from 4.2% to 28.1%. Overall quality of the studies was low.

**Conclusions:**

Further research is required to elucidate the effectiveness and potential impact of text message interventions on weight and weight-related behaviors in adolescents.

## Introduction

### Background

The prevalence of overweight and obesity among children and adolescents is now estimated to be 18% globally, with the prevalence of overweight increasing by 47% over the last four decades [1]. Lifestyle risk factors, namely, suboptimal diet, physical inactivity, and overweight and obesity are well established during adolescence [2-4], making this second decade of life (10-19 years) [5] a critical period for the development of lifelong health trajectories [6]. Weight gain during adolescence is associated with earlier onset type 2 diabetes mellitus and cardiovascular disease (CVD) [2,7]. Compared with adolescents without obesity, adolescents with obesity have increased levels of circulating free fatty acids, which reduce insulin sensitivity, potentially contributing to impaired insulin secretion [7]. Moreover, adolescents who gain weight and maintain a high BMI into adulthood have higher odds of developing hypertension, dyslipidemia, and systemic inflammation [2]. More short-term adverse health outcomes associated with adolescent overweight and obesity include weight stigma [8] and reduced quality of life and self-esteem [9]. Interventions to prevent obesity, including combinations of both physical activity and diet interventions have so far remained mainly school based and with limited evidence of effectiveness on BMI [10]. Therefore, for health systems to impact population overweight and obesity, interventions need to be personalized, low cost, and scalable with a broad reach to engage all adolescents at risk of obesity.

The ubiquity of mobile phones has given rise to new ways of delivering health care through mobile health (mHealth) [11]. mHealth has the potential to provide personalized, low-cost, and population-wide behavior change programs for obesity prevention and management, particularly for adolescents who are digital frontrunners [12]. Adolescent mobile phone ownership is high in both developed and developing countries. For example, over 90% of adolescents in Australia and the United States own a mobile phone [13,14] and the average age to first own a mobile phone was 10.4 years in Korean children [15]. Moreover, adolescents spend on average more than 3 hours per day using a mobile phone, with their device always nearby [16], and text messaging remains a primary means of communication [13]. As such, text message interventions have the potential to be a wide-reaching and engaging mode of health care delivery for adolescents.

There is evidence which indicates that text messaging interventions in adults can promote improvements in lifestyle behavior change for weight loss, smoking cessation, physical activity, management of chronic disease and CVD risk, blood pressure lowering, and diabetes care [17-22]. Text messages are a feasible and acceptable form of communication for adolescents with obesity [23,24]. Features of text messages that are well suited to dietary and physical activity behavior change interventions for adolescent weight management include two-way communication between participant and health professional; direct communication with participants (ie, not required to actively log on to a smartphone app or website for support); and the ability to prompt and reward the repetition of positive behavior change in real time.

A critical step to translate and disseminate mHealth obesity prevention and management programs for adolescents is to understand the effectiveness and acceptability of the mHealth intervention components, which may help improve the integration of evidence-based practices into health systems. Previous systematic reviews have found limited studies utilizing text messages for improving diet and physical activity behaviors of adolescents [25,26]. However, Rose et al [26] did identify successful behavior change strategies for improved nutrition and physical activity behaviors, namely, education, goal setting, and self-monitoring, which are feasible for delivery via text message. Another review investigating mobile technologies for the prevention and treatment of pediatric obesity found considerable heterogeneity in the three included studies utilizing text messages [27].

### Objective

In recent years, evidence on the effectiveness of interventions delivered via text messages for adolescent weight management have been accumulating but not yet consolidated. Moreover, the collective evidence on text message development and process evaluation data, such as how adolescents engage with and use the text messages, have not been synthesized. Therefore, the aim of this systematic review was (1) to evaluate the effect of interventions delivered via text messages on weight outcomes in an adolescent population and (2) to understand intervention characteristics that are common to effective interventions delivered by text message.

## Methods

### Protocol and Registration

This systematic review was conducted and reported following the Preferred Reporting Items for Systematic Reviews and Meta-Analysis (PRISMA) statement guidelines [28] (Multimedia Appendix 1) and followed the predetermined methods documented in a protocol. The review was registered in the International Prospective Register of Systematic Reviews (PROSPERO; registration number: CRD42018109197).

### Eligibility Criteria

Studies were included that met the following criteria: (1) randomized controlled trials (RCTs) that included lifestyle interventions of any duration which included mobile phone SMS or text message. Only RCTs were included as they provide the strongest evidence for the benefits of a health care intervention [29]; (2) participants were adolescents, defined by the World Health Organization as the second decade of life, 10 to 19 years [5], both girls and boys, not pregnant and free of acute illness or chronic disease, as some conditions may influence body weight outcomes or ability to change lifestyle behaviors; (3) only studies focused on obesity prevention or management were included; and (4) studies with interventions of any duration that involved the delivery of text messages via a mobile phone device, including multicomponent interventions that were delivered in part by text message. Studies with interventions that use at least any two of the behavioral techniques (BCTs) to achieve behavior change, for example, goal setting, were included. BCTs are defined as a mechanism of action in an intervention that contributes to positive behavior change [30]. The mode of text message delivery included standard SMS or messaging apps like WhatsApp (studies were excluded if they included text messages that are only appointment or other reminders); (5) all study settings were included, that is, health care, community, home, or school based; (6) a comparator group of participants receiving standard care (no messages or some form of control text message); (7) a study outcome of change in body weight measured in BMI or BMI z-score (also called BMI standard deviation scores); (8) studies published in any language were considered; and (9) studies published after 2005 were considered. The cut-off date of 2005 was selected as the current generation of adolescents (*Generation Z*) appeared in the population after 1995, and the oldest of this generation were 10 years old in 1995. The criteria for included studies in this review are summarized in the Population, Intervention, Comparator, Outcomes, and Setting format in Table 1.

**Table 1:** Summary description of Population, Intervention, Comparator, Outcomes, and Setting (PICOS) components.

PICOS components	Description
Population	Individuals (adolescents, 10-19 years) of any demographic background
Intervention	Interventions that include mobile phone SMS or texting intervention
Comparator	Intervention vs usual care
Outcomes	Changes in body weight measured in terms of BMI and/or BMI z-score^a^
Setting	Randomized controlled trials conducted in any setting

^a^Units BMI is above or below average BMI for age- and sex-specific reference values.

### Information Sources and Searches

A total of 10 major electronic databases (Pre-Medline, MEDLINE, Cochrane, Cochrane Central Register of Controlled Trials, Excerpta Medica Database, Cumulative Index of Nursing and Allied Health Literature, Allied and Complementary Medicine Database, Informit, Scopus, and Web of Science) were systematically searched until January 21, 2019, and hand searching was conducted until July 12, 2019. The database searches were developed in collaboration with a librarian. Search terms included combinations, truncations, and synonym of the following: (1) adolescent; (2) phone text messaging (mHealth or *telemedicine* or *tele-medicine* or *mobile phone* or *mobile phones* or *cell phones* or *cell phone* or *cell phones* or SMS or *short message service* or *short message* or texting or *text message* or *text messages* or *text messaging* or *text messaged* or *text messaging* or txt or text); and (3) weight loss or weight maintenance (*diet, reducing* or *weight reduction programs* or *weight loss* or *weight maintenance* or *weight loss* or *weight management* or weight and loss or manage* or maintenance or maintain or maintain*). Appropriate RCT filters were used to maximize the identification of RCTs. Additional articles were obtained through a hand search of reference lists, conference proceedings, key journals in the field, abstracts, clinical trial databases, and by contacting experts in the field. Full electronic search strategies for each database and screenshots of all search results are available in Multimedia Appendices 2 and 3.

### Study Selection

One author (SP) carried out all electronic database searches. Search results across databases were merged using reference management software, Endnote X8 (Camelot UK Bidco Limited, Clarivate Analytics, United Kingdom), and duplicate records of the same study were removed. Study selection followed the process described in the Cochrane Handbook of Systematic Reviews and PRISMA statements. Two researchers (SP and RR) independently screened titles and abstracts to remove irrelevant studies to identify studies that met the inclusion criteria described above using a predetermined eligibility assessment form. Any disagreements were discussed and resolved by consensus between two authors (SP and RR), and a third author was consulted (AS) in the case of unresolved disagreement.

### Data Collection Process

For studies meeting the inclusion criteria, information was extracted using a predesigned electronic data extraction table based on PRISMA statement [28] and data items required for the Cochrane Collaboration’s risk of bias tool [29] that was developed and test-piloted for this review. One author extracted (SP) the data, and a second author (RR) independently cross-checked a random 20% of the data for accuracy. Extracted data included data items on study characteristics (design, aim, sample size, active intervention duration, extended intervention duration, follow-up time points, attrition, comparison of dropouts, and recruitment methods), participant characteristics (age, weight, BMI (kg/m^2^) and/or BMI z-score at baseline, gender, and ethnicity), intervention characteristics (components, theoretical underpinning, BCTs using the behavior-specific taxonomy of 40 BCTs for physical activity and healthy eating behaviors [CALO-RE taxonomy] [30], setting, peer support, personnel interaction, text message details, and comparators), study outcome measures (method of assessment, changes in body weight measured in BMI change in kg/m^2^, and/or BMI z-score change in units during the intervention and all follow-up period[s], changes in diet, physical activity and/or psychosocial well-being if reported or collected, measures of error, and statistical significance), and adherence measures (number, type, and definition of adherence measures). When two or more articles reported results from the same study, all articles were considered together for complete data extraction. Authors were contacted for missing, incomplete, or unclear data.

### Data Synthesis and Analysis

The key characteristics of the included studies were summarized in text form and tabulated using the information collected from the data extraction form. The primary outcome of interest was the change in body weight measured in BMI change in kg/m^2^ or BMI z-score change in units. Where possible, for all study arms, the mean or median change was recorded at baseline, postintervention, and any additional follow-up(s). Measures of error were standard error or standard deviation and associated *P* values for change between groups at follow-up(s), and overtime were recorded if available. The clinical significance of outcomes was also considered. Modest reductions in BMI z-score (0.01-0.15) in adolescents with overweight or obesity have been associated with improvements in several cardiovascular risk factors and considered to be clinically meaningful [31]. More significant improvements in cardiovascular risk can be seen with BMI z-score reductions of >0.25 in adolescents with obesity [32]. Interventions were heterogeneous; only 5 studies reported the mean change in BMI, only 4 studies reported the mean change in BMI z-score, and all were at varying follow-up time points. This heterogeneity rendered the sample size too small for a meta-analysis of these outcomes to be conducted at each follow-up time point [33].

### Risk of Bias Assessment

#### Cochrane Risk of Bias Assessment

The Cochrane Collaboration’s tool was used to assess the risk of bias at the individual study level [29]. The primary sources of systematic bias in trials were assessed including the selection of participants (random sequence generation and allocation concealment methods); performance (blinding of participants and personnel); detection (blinding of outcome assessment); attrition (incomplete outcome data); and reporting (selective reporting of study outcomes). Two authors (SP and RR) independently evaluated each study for risk of bias and permitted a judgment of low risk, high risk, or unclear risk. A third author (AS) was consulted in the case of unresolved disagreement.

### Quality of Evidence Assessment

#### Grading of Recommendations Assessment, Development, and Evaluation Assessment

The quality of the body of evidence was determined using the Grading of Recommendations Assessment, Development and Evaluation (GRADE) system [34]. In total, 5 categories were considered to ascribe a quality rating: limitations in study designs; consistency of results; the directness of the evidence concerning study populations, intervention design, and outcomes measured; the precision of outcomes; and the presence of publication biases. Two authors (SP and RR) independently evaluated the quality of the body of evidence. A third author (AS) was consulted in the case of unresolved disagreement.

## Results

### Study Selection

**Figure 1:**
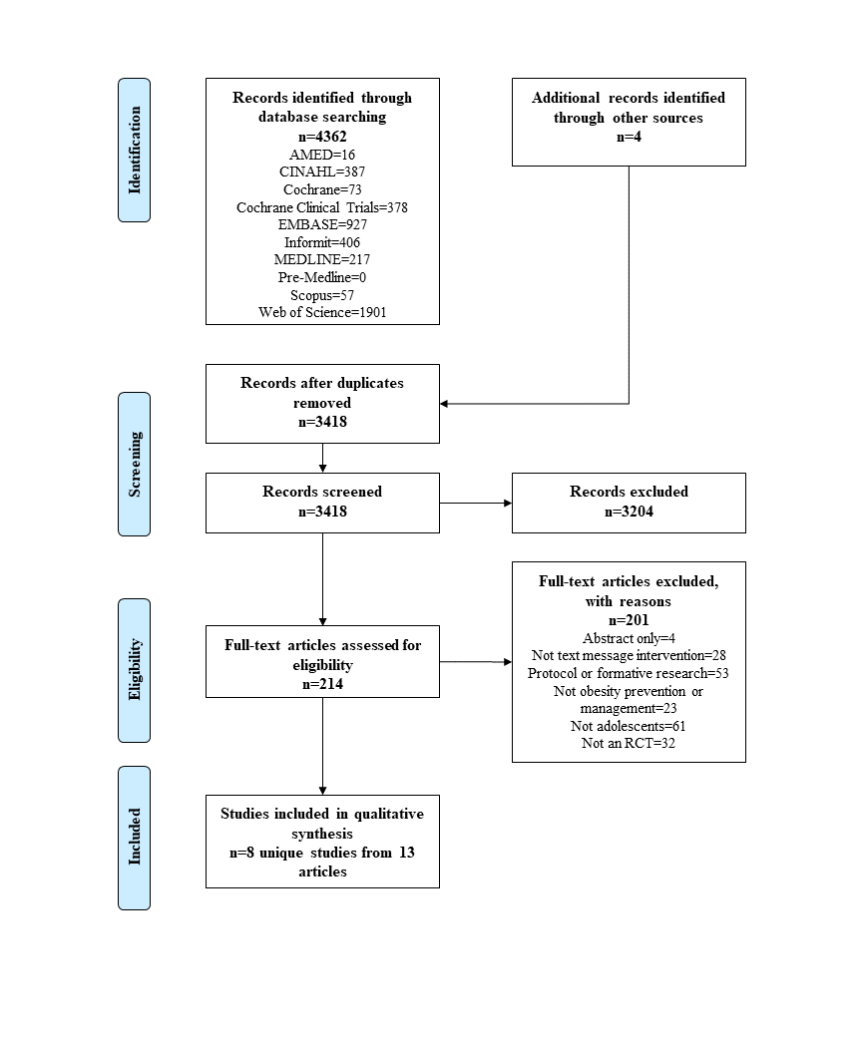
Preferred Reporting Items for Systematic Reviews and Meta-Analysis (PRISMA) flow diagram of included studies. RCT: randomized controlled trial. CINAHL: Cumulative Index of Nursing and Allied Health Literature; AMED: Allied and Complementary Medicine Database.

The search found 4362 articles from all electronic database searches and an additional 4 articles through hand searching of reference lists (Figure 1). After exclusion of duplicates, 3418 articles were screened by title and abstract, and 3203 were excluded. A total of 215 full-text articles were assessed for edibility, and 201 were excluded with reasons outlined in Figure 1 and Multimedia Appendix 4. Eight unique studies [35-42] from 13 publications were included in this review [24,35-41,43-47].

### Effectiveness of Interventions

#### Body Mass Index

All studies measuring BMI (kg/m^2^) measured height and weight in person by trained personnel using standardized procedures and calibrated equipment (Table 2). The 5 studies calculating BMI z-score specified the country of origin for the age- and sex-specific reference values [37,39-42]. Of the 8 studies, 7 demonstrated reductions in BMI or BMI z-score in the intervention group compared with the control at the end of the final follow-up (Table 3). The effect was only statistically significant in 1 study at 6 months [37]. Significant time by group effects were observed in 3 studies [36,37,40]; 2 studies reported the effect of the intervention on both BMI and BMI z-score [37,40], 3 studies reported the effect of the intervention on BMI only [35,36,38], and 3 studies reported the effect of the intervention on BMI z-score only [39,41,42]. A decrease in BMI ranged between 1.3% and 4.5% in the intervention group compared with the control at 6 months, with the exception of 1 study, which saw an increase in BMI of 3.8% in the intervention group compared with the control group at 6 months [38]. The 3 studies which reported BMI z-score change at 6 months varied with a decrease of 28.1%, 4.2%, and 4.5% in the intervention group compared with the control [37,41,42].

**Table 2:** Method of BMI and BMI z-score assessment (n=8).

Parameter, author, year, country		Method of assessment
**BMI**		
	Abraham et al, 2015, China [35]	Height and weight measured in person, by a trained researcher using standard procedures and calibrated equipment
	Bagherniya et al, 2018, Iran [36]	Height and weight measured in person by a trained researcher in duplicate using standard procedures and calibrated equipment
	Chen et al, 2017, the United States [37]	Height and weight measured in person, by trained research assistant who was blinded to group assignment
	Love-Osborne et al, 2016, the United States [38]	Height and weight measured in person with shoes off, repeated twice by study staff
	Nguyen et al, 2012, Australia [40,46]	Height and weight measured in person by a trained researcher using standard procedures and calibrated equipment
**BMI z-score**		
	Chen et al, 2017, the United States [37]	BMI z-scores based on age- and sex-specific reference values (US reference data)
	Jensen et al, 2019, the United States [42]	BMI z-scores based on age- and sex-specific reference values (US reference data)
	Mameli et al, 2018, Italy [39]	BMI z-scores based on age- and sex-specific reference values (Italian reference data)
	Nguyen et al, 2012, Australia [40,46]	BMI z-scores based on age- and sex-specific reference values (US reference data)
	Patrick et al, 2013, the United States [41]	BMI z-scores based on age- and sex-specific reference values (US reference data)

**Table 3:** Mean change in BMI or BMI-z score at follow up(s) (n=8).

Author, year, citation, outcome, follow-up timepoint (months), and study arms					Mean difference within groups (SD)		Effect size (%)		Level of effect size^a^		Mean difference between groups		Mean difference between groups over time
**Abraham et al, 2015, China [35]**													
	**BMI**												
		**3**				**0.6 decrease**		**+**		***P*=.14**		**NR^b^**	
			IT^c^	0 (NR)									
			sLMP^d^	0.4 (NR)									
			C^e^	0.2 (NR)									
		**6**				**1.3 decrease**		**+**		***P*=.07**		**NR**	
			IT	−0.1 (NR)									
			sLMP	−0.5 (NR)									
			C	0.3 (NR)									
**Bagherniya et al, 2018, Iran [36]**													
	**BMI**												
		**3.5**											
			I^f^	NR		NR		NR		NR		NR	
			C	NR		—^g^		—		—		—	
		**7**											
			I	−0.7 (NR)		3.9 decrease		**+**		*P*=.13		*P*<.001	
			C	0.4 (NR)		—		—		—		—	
**Chen et al, 2017, the United States [37]**													
	**BMI**												
		**3**											
			I	−0.4 (NR)		3.3 decrease		**+**		NR		NR	
			C	0.46 (NR)		—		—		—		—	
		**6**											
			I	−0.44 (NR)		4.5 decrease		**+ +**		−1.05 (SE 1.22, 90% CI −3.09 to 0.95); *P*=.39		−0.58 (SE 0.13, 90% CI −0.84 to −0.40); *P*=.001	
			C	0.83 (NR)		—		—		—		—	
	**BMI z-score**												
		**6**											
			I	−0.18 (NR)		28.1 decrease		**+ + +**		−0.15 (SE 0.15, 90% CI −0.40 to 0.09); *P*=.001		−0.12 (SE 0.03, 90% CI −0.16 to −0.07); *P*=.001	
			C	0.26 (NR)		—		—		—		—	
**Jensen et al, 2019, the United States [42]**													
	**BMI z-score**												
		**6**											
			I	−0.09 (NR)		4.2 decrease		**+**		Not statistically significant, *P* value, NR		NR	
			C	−0.03 (NR)		—		—		—		—	
**Love-Osborne et al, 2016, the United States [38]**													
	**BMI z-score**												
		**6-8**											
			I	1.2 (NR)		3.8 increase		**+**		NR		NR	
			C	0.0 (NR)		—		—		—		—	
**Mameli et al, 2018, Italy [39]**													
	**BMI z-score**												
		**3**											
			I	NR		NR		NR		0.00 (95% CI −0.11 to 0.12); *P*>.05		0.01 (95% CI −0.15 to 0.18); *P*=.87	
			C	NR		—		—		—		—	
**Nguyen et al, 2012, Australia [40,46]**													
	**BMI**												
		**2**											
			I	NR		NR		NR		NR		NR	
			C	NR		—		—		—		—	
		**12**											
			I	0.6 (NR)		1.9 increase		**+**		0.1 (95% CI −1.2 to 1.3); *P*>.05		0.1 (95% CI −0.3 to 0.4); *P*>.05	
			C	0.0 (NR)		—		—		—		—	
		**24**											
			I	0.0 (NR)		3.2 decrease		**+**		0.1 (95% CI −1.2 to 1.3); *P*>.05		0.8 (95% CI 0.2 to 1.4); *P*<.05	
			C	1.0 (NR)		—		—		—		—	
	**BMI z score**												
		**2**											
			I	NR		NR		NR		NR		NR	
			C	NR		—		—		—		—	
		**12**											
			I	−0.06 (NR)		1.0 increase		**+**		−0.00 (95% CI −0.11 to 0.10); *P*>.05		−0.09 (95% CI −0.12 to −0.06); *P*<.001	
			C	−0.08 (NR)		—		—		—		—	
		**24**											
			I	−0.2 (NR)		5.4 decrease		**+ +**		−0.01 (95% CI −0.11 to 0.10); *P*>.05		−0.13 (95% CI −0.20 to −0.06); *P*<.05	
			C	−0.09 (NR)		—		—		—		—	
**Patrick et al, 2013, the United States [41]**													
	**BMI z score**												
		**6**											
			W^h^	−0.1 (NR)		4.5 decrease		**+ +**		NR		*P*=.93	
			WG^i^	0.0 (NR)		—		—		—		—	
			WSMS^j^	−0.1 (NR)		—		—		—		—	
			C	0.0 (NR)		—		—		—		—	
		**12**				**4.5 decrease**		**+ +**		**NR**		**—**	
			W	−0.1 (NR)									
			WG	−0.2 (NR)									
			WSMS	−0.1 (NR)									
			C	0.0 (NR)									

^a^+ denotes effect size 0%–4%; + + denotes effect size 5%–9%; + + + denotes effect size ≥10%.

^b^NR: not reported.

^c^IT: Internet intervention group.

^b^NR: not reported.

^d^sLMP: Simplified Lifestyle Modification Program intervention group.

^e^C: Control.

^f^I: Intervention.

^g^Not applicable.

^h^W: website only.

^i^WG: website + group.

^j^WSMS: website + text messages.

At 3 months, Abraham et al [35] investigated the mean difference in BMI (kg/m^2^) between the intervention and control groups and found no effect (*P*=.04) on BMI (kg/m^2^). At 6 months, 3 studies investigated the mean difference in BMI between the intervention and control groups and found no effect [36-38]. Two studies found significant group by time effects for mean decreases in BMI (kg/m^2^) from baseline to 6 months [36,37]. Chen et al [36] reported a decrease in BMI of 0.58 kg/m^2^ (SE 0.13; 90% CI −0.84 to −0.40; *P*=.001) [37] and the other study reported only a value of *P*<.001 [36]. Nguyen et al found that the intervention with adjunctive electronic contact including text messages (once per month from 2 months) compared with group-based behavioral lifestyle intervention only had no effect at 12 (BMI 0.01 kg/m^2^; 95% CI −1.2 to 1.3; *P*>.05) [40] or 24 months (BMI 0.01 kg/m^2^; 95% CI −1.2 to 1.3; *P*>.05) [46]. From baseline to 24 months, there was a significant group by time effect for BMI, with a mean decrease between the intervention and control groups of 0.8 kg/m^2^ (95% CI 0.2 to 1.4; *P*<.05) [46].

At 3 months, Mameli et al [39] found comparable mean decreases in BMI z-score between the intervention and control groups (−0.03 units, 95% CI 0.14 to 0.09 vs −0.04 units, 95% CI 0.16 to 0.08, respectively), which were not significantly different (*P*>.05) and no time effect from baseline to 3 months was observed (*P*=.87). At 6 months, Chen et al [37] found a significant mean difference in BMI z-scores between groups of −0.15 units (SE 0.15; 90% CI −0.40 to 0.09; *P*=.001) and a significant decrease in BMI z-score over time between groups (−0.12 units; SE 0.03; 90% CI −0.16 to −0.07; *P*=.001). Jensen et al [42] found that BMI z-score did not differ across the intervention and control groups at 6 months (*P* value not reported). However, participants in the intervention group significantly decreased BMI z-score by 0.32 units compared with baseline values (*P*<.01), whereas the control group participants did not decrease BMI z-score over the 6 months (*P*=.63). Similar to the results for BMI, Nguyen et al [40,46] found no significant difference in BMI z-score between groups at 12 and 24 months (*P*>.05). However, from baseline to 12 and 24 months, there was a significant group by time effect for BMI z-score, with a mean decrease between the intervention and control groups of 0.09 units (95% CI −0.12 to −0.06; *P*<.001) and 0.13 units (95% CI −0.20 to −0.06; *P*<.05), respectively. Patrick et al [41] found no difference in BMI z-score from baseline, 6, and 12 months between groups (*P*=.93).

#### Lifestyle Outcomes

There was limited overlap in secondary outcomes between studies, which limited comparisons. In total, 3 studies measured blood pressure, and no consistent significant differences were shown between the intervention and control groups. Abraham et al [35] found no decrease in systolic or diastolic blood pressure between the intervention and control groups. Chen et al [37] found a significant reduction in diastolic blood pressure of 2.66 mmHg over 6 months between the intervention and control groups (90% CI −4.02 to −1.31; *P*=.001). Whereas Nguyen et al [40] found the intervention group had a mean higher systolic blood pressure difference of 3 mmHg at 12 and 24 months only after adjusting for sex, age, and perceived athletic ability. Bagherniya et al [36] and Nguyen et al [40] found no effect of the intervention on waist circumference at 6 months or 12 and 24 months, respectively. A total of 4 studies measured physical activity using different instruments, including a 0 to 10 scale of physical activity validated in Chinese youth [35], a short question adapted from the California Health Interview Survey [37], and a 7-day physical activity recall interview [41] or physical activity questionnaire [36]. Chen et al [37] observed a significant increase of 0.40 physical activity days per week in the intervention group compared with the control group over the 6 months (90% CI 0.15 to 0.66; *P*=.01) [37] and Bagherniya et al [36] found the intervention group significantly increased daily minutes of physical activity compared with controls at 6 months (*P*<.001). Two studies assessed the quality of life using the Impact on Weight Quality of Life–Kids [42] and the pediatric quality of life inventory [41]. Jensen et al [42] found no between-group difference in the weight-related quality of life. However, both groups demonstrated significant improvements in parent-report scores (*P*s<.05) [42], and Patrick et al [41] found positive correlations between physical functioning quality of life and behavior change strategies in girls. Body fat percentage was measured in 2 studies at 6 months, and no differences were observed [35,41]. Dietary intakes were measured in 2 studies via short self-report questions on fruit, vegetable, and sugar-sweetened beverage intake [37] or by a food frequency questionnaire [41] at 6 months, and no differences were observed. Other outcomes included waist-to-height ratio, stress, depression self-efficacy, and social support for behavior change, of which none were consistent between studies.

### Study Characteristics

All RCTs included in the review were published in English [35-41]. The median sample size was 75 participants (range 40-172 participants), with a total of 767 participants (Table 4). In all, 2 studies recruited participants from obesity clinics [35,39], 3 studies recruited from primary care [37,41,42], 2 studies recruited from schools [36,38], and the remaining study utilized several recruitment strategies [40,45] (Table 5). Participants ranged in age from 12 to 18 years, with a mean age of 14.3 years (SD 0.9). All participants had overweight (BMI on 85th-95th percentile) or obesity (BMI>95th percentile) with a mean BMI of 29.7 kg/m^2^ (SD 1.6). One study reported the mean participant BMI percentile for age and sex at baseline, which was 91.5 (SD 4.2) [42]. On average, 53% of participants were female (range 27%-100%). Overall, 6 studies reported participant ethnicity [35-38,41,42], with 2 studies recruiting 100% of participants who were Chinese [35,37]; in 1 study, 100% of the participants were Persian [36]; in 1 study, 64% of the participants were white [42], and the samples in the remaining 2 studies were predominately Hispanic [38,41]. In all, 5 studies with a follow-up at 2 to 3 months had a median attrition rate of 6.8% (range 0%-30.4%) [35-37,39,40]. The median attrition rate increased for each subsequent follow-up: 15% at 6 to 8 months (range 0%-38.5%, n=6 studies) [35-38,41,42]; 26.9% at 12 months (range 12.3%-46.2%, n=2 studies) [40,41]; and 38.5% at 24 months (range 25.9%-41.1%, n=1 study) [40].

**Table 4:** Characteristics of included studies (n=8).

Author, year, country	Study design	Total (n)	I^a^ (n)	C (n)	Active (months)^b^	Extended (months)^b^	Follow-up(s) (months)	Attrition at follow-up(s) (%)	Dropouts compared
Abraham et al, 2015, China [35]	RCT^c^	48	IT^d^: 16; sLMP^e^: 16	16	3	3	3, 6	3 months: 0; 6 months: 0	Not applicable
Bagherniya et al, 2018, Iran [36]	RCT	172	87	85	7	0	3.5, 7	3.5 months: I: 10.3; C^f^: 2.4; 7 months: I: 16.1; C: 4.7	Not reported
Chen et al, 2017, the United States [37]	RCT	40	23	17	3	3	3, 6	3 months: I: 0; C: 0; 6 months: I: 8.7; C: 11.8	Not reported
Jensen et al, 2019, the United States [42]	RCT	47	29	18	6	0	6	6 months: I: 34; C: 33	No difference
Love-Osborne et al, 2016, the United States [38]	RCT	165	TM^g^: 38; NTM^h^: 44	83	6-8	0	6-8	I: 5; C: 11	No difference
Mameli et al, 2018, Italy [39]	RCT	43	23	20	3	0	3	I: 30.4; C: 25	No difference
Nguyen et al, 2012, Australia [40,46]	RCT	151	73	78	2	22	2, 12, 24	2 months: I: 6.8; C: 11.5; 12 months: I: 12.3; C: 15.4; 24 months: I: 41.1; C: 35.9	No difference
Patrick et al, 2013, the United States [41]	RCT	101	W^i^: 26; WG^j^: 26; WSMS^k^: 24	25	12	0	6, 12	6 months: W: 31.0; WG: 38.5; WSMS: 22.7; C: 37.5; 12 months: W: 31.0; WG: 46.2; WSMS: 22.7; C: 33.3	No difference

^a^I: intervention.

^b^Intervention duration.

^c^RCT: randomized controlled trial.

^d^IT: Internet intervention group.

^e^sLMP: Simplified Lifestyle Modification Program intervention group.

^f^C: control.

^g^TM: text message.

^h^NTM: no text message.

^i^W: website only.

^j^WG: website + group.

^k^WSMS: website + text messages.

**Table 5:** Characteristics of participants from included studies (n=8).

Author, year, country	Recruitment of population	Age range, years	Age (years), mean (IQR or SD)	BMI range	BMI (kg/m^2^), mean (IQR or SD)	Female (%)	Ethnicity, other SES^a^ factors (%)
Abraham et al, 2015, China [35]	Obesity clinic	12-18	IT^b^: 14.9^c^ (13.7-16.2); sLMP^d^: 14.1^c^ (13.5-15.3); C^e^: 14.3^c^ (13.5-15.8)	>95th	IT: 29.3^c^ (26.7-30.9); sLMP: 31.5^c^ (29.8-33.7); C: 30.1^c^ (28.4-32.3)	39.6	Chinese: 100; Parent tertiary education: 27
Bagherniya et al, 2018, Iran [36]	Government and private schools	12-16	I^f^: 13.5 (0.7); C: 13.4 (0.6)	≥85th	I: 29.2 (3.9); C: 27.2 (2.9)	100	Persian: 100; Parent tertiary education: 100
Chen et al, 2017, the United States [37]	Primary care providers	13-18	I: 15.0 (1.7); C: 14.8 (1.6)	≥85th	I: 27.4 (3.3); C: 28.4 (4.4)	42	Chinese American: 100; Parent mean years of education: 10
Jensen et al, 2019, the United States [42]	Primary care pediatric practices	12-18	15.0 (1.5)	≥85th; <95	91.5 (4.2) (BMI %)	79	Hispanic: 23; African American: 2
Love-Osborne et al, 2016, the United States [38]	Public schools	12-18	I: 15.7 (1.5); C: 16.0 (1.5)	≥85th	I: 31.9 (6.2); C: 31.6 (6.5)	I: 58; C: 46	Hispanic: I: 88 and C: 89
Mameli et al, 2018, Italy [39]	Obesity clinic	10-17	I: 12.6 (1.7^g^); C: 12.4 (2.2^g^)	≥95th	I: 29.6 (3.3^g^); C: 28.6 (2.6^g^)	I: 31^g^; C:43^g^	Parent tertiary education: 17^g^
Nguyen et al, 2012, Australia [40,46]	Media, schools, health professionals and community organizations	13-16	I: 14.0 (0.9); C: 14.2 (1.0)	1.0-2.5^h^	I: 30.8 (4.2); C: 30.8 (3.5)	27	Not reported
Patrick et al, 2013, the United States [41]	Pediatric primary care	12-16	W^i^: 14.1 (1.4); WG^j^: 14.3 (1.5); WSMS^k^: 14.3 (1.8); C: 14.5 (1.5)	>85th	W: 2.2 (0.07)^l^; WG: 2.2 (0.07)^l^; WSMS: 2.2 (0.07)^l^; C: 2.2 (0.07)^l^	63	Hispanic: 74

^a^SES: socioeconomic status.

^b^IT: Internet intervention group.

^c^Median age and BMI (IQR).

^d^sLMP: Simplified Lifestyle Modification Program intervention group.

^e^C: control.

^f^I: intervention.

^g^Completers-only data

^h^95% confidence intervals

^i^W: website only.

^j^WG: website + group.

^k^WSMS: website + text messages.

^l^BMI z-score (standard error).

### Intervention Characteristics

A total of 5 studies were two-arm RCTs [36,37,39,40,42], 2 were three-arm RCTs [35,38], and 1 a four-arm RCT (Table 5) [41]. The median active intervention period was 4.5 months (range 2-12 months) and 2 studies had an extended intervention period of 3 months [35,37], and 1 had 22 months [40]. In all, 5 studies followed up participants at 2 to 3 months [35-37,39,40], 6 studies at 6 to 7 months [35-38,41,42], 2 studies at 12 months [40,41] and 1 study at 24 months [40]. All interventions were multicomponent, and no interventions were delivered solely via text messages (Table 6). Two studies had intervention groups consisting of Web-based educational modules delivered via a website and text messages [35,41]; 2 studies had intervention groups which provided participants wearable devices and access to smartphone apps and text messages [37,39], and the remaining 4 studies had intervention groups which involved in-person individual or group sessions with health professionals and text messages [36,38,40,42]. In all, 4 studies were grounded in the social cognitive theory [35-37,40]. Overall, 2 studies were primarily set in an obesity clinic [35,39], 2 in high schools [36,38], 3 in primary care [37,40,42], and 1 was mostly online [41]. The majority of studies encouraged peer support from parents, and all studies included interactions with research and health professionals. Control conditions consisted of usual care, with 6 of the 8 studies including control conditions with in-person educational or information sessions [35,36,39-42].

The median number of text messages sent each week was 1.5 (range 1-21 text messages; Table 7). One study specified that 3 text messages were sent each day; however, it was not specified if this was every day [42]. In this study, control participants also received a form of control text messages, and both groups demonstrated reductions in BMI z-score [42]. In all, 4 studies used one-way text messages (participants unable to reply) [36-39] and 4 studies used two-way text messages (participants able to respond) [35,40-42]. A total of 4 studies had personalized text messages [38,39,41,42], 2 studies had semipersonalized text messages [35,40], and 2 studies did not have personalized text messages [36,37]. Overall, 6 studies utilized text messages in the active intervention phase, ranging from 17% to 100% of the intervention contact points [35,36,38,39,41,42]. In all, 2 studies utilized text messages only in the extended intervention phase accounting for 100% [37] and 60% [40] of the intervention contact points. Both studies with text messages only in the extended phase had significant between-group differences in BMI or BMI z-score at the end of follow-up [37,40]. The 4 studies, which personalized the text messages, had text message content–related individual behavioral goals [35,38] or utilized data from wearables [39] or self-reported text messages [42] to provide personalized feedback on lifestyle behaviors. All 4 studies did not significantly affect BMI outcomes. The BCTs used in the text messages are presented in Multimedia Appendix 5. The number of domains ranged from 3 to 5, with all text messages in each study utilizing goals and planning and credible source. The number of BCTs ranged from 4 to 8, with all text messages in each study using goal setting for behaviors and credible source.

A total of 3 studies provided details about the development of the intervention content broadly [37] or text messages [24,35,42]. Abraham et al [35] conducted focus groups with 11 Chinese American adolescents asking open-ended questions about the use of text messages in the program, and adolescents agreed that individualized weekly text message reminders could be a good way to enhance motivation to adopt healthy behaviors. No further details about the text message development were provided. Chen et al [37] formed an advisory group consisting of two primary care physicians and four adolescents and consulted other adolescents (n=10) via a focus group. The advisory group identified the purpose and goals of the intervention and focus groups and piloted the procedures and appropriateness of the intervention content. Adolescents *liked the program* and minor changes were made. No specific details relating to text message development were provided. In a separate publication, Jensen et al [24] detailed the process of text message development. The iterative process involved the development of an evidence-based text message bank by the research team, which was then tested in a pilot study with 20 adolescents (mean age 14 years, 85% female, 77% white) who received 1 text message per day for 3 months [24]. Feedback was provided at monthly intervals and at the end of the study. Overall, participants liked the text messages, liked receiving them after school, and they could recall topics the text messages addressed. The most popular text messages were recipe ideas, followed by testimonials and messages with pictures. Most found the messages personally relevant, and messages helped them to make healthy choices and kept them focused on weight management. A greater variety of messages were suggested. The findings informed the RCT.

In total, 6 studies provided results related to the rates of replies of text messages during the intervention. Abraham et al [35] found 78.3% of participants responded to text messages about diet goals and 77.5% of participants responded to text messages about exercise goals. In a study by Nguyen et al [40], response rates were lower, with messages about healthy eating and booster session reinforcement having the highest reply rates (42% and 34%, respectively), whereas text messages about self-esteem or stress management had the lowest response rates (4%). Jensen et al [42] found over 6 months that intervention participants sent self-monitoring text messages on 47% of intervention days compared with only 22% control participants [42]. Intervention participants demonstrated significantly higher self-monitoring adherence (*P*<.01) than control participants. The 3 text messages per day were helpful for 85% of intervention participants. However, 69% of intervention participants found the text message content annoying and repetitive. Over half (54%) would prefer fewer and more personalized text messages.

Love-Osborne et al [38] sent an average of 12 personal goal-related text messages and 12 text messages to remind participants to return log sheets about their weekly weight and lifestyle behaviors. However, it was reported that only few adolescents turned in log sheets in either group. Only 1 study asked participants to rate the helpfulness of text messages, with 27 of 39 adolescents rating the messages *somewhat helpful* or *very helpful* [40]. One study, which included Fitbit and website education resource in addition to text messages, found that the 100% of participants would recommend the program to others and 91% shared the Fitbit data with their primary care provider [37]. Only 1 study detailed that text messages were sent from an automated computer system capable of tailoring the text messages using an algorithm based on participant feedback via text message [42]. No other studies provided details about the system or infrastructure used to deliver the text messages. One study reported a research assistant spent 2 hours per week sending personalized text messages [35].

**Table 6:** Characteristics of interventions (n=8).

Author, year, country, and intervention description		Theory	Setting	Peer support	Intervention personnel interaction	Comparator description	Comparator personnel interaction
**Abraham et al, 2015, China [35]**							
	IT^a^: usual care + 12 website lessons + text messages	TTM^b^; SCT^c^	Obesity clinic; website; mobile phone	Parents	IT: usual care	Usual care: physician check-ups with obesity counselling	3 in-person sessions for adolescents with their parent(s)
	sLMP^d^: usual care+ 4 counselling sessions	SCT	Obesity clinic	Parents	sLMP: usual care + 4 in-person counselling sessions with nutritionist		
**Bagherniya et al, 2018, Iran [36]**							
	I^e^: 14 sports workshops + 7 counselling sessions + up to 60 fun exercise sessions + up to 60 competitive sports sessions + up to 3 family activity sessions + text messages^f^	SCT	School; local gyms; mobile phone	Parents; teachers	I: 14 in-person sport group workshops (personnel not specified) + 7 in-person sports counselling sessions (personnel not specified) + up to 56 fun group exercise sessions with specialist in physical education	Control group: education classes, lectures, printed handbook	3 in-person educational classes for adolescents; 2 in-person lectures for parents/teachers
**Chen et al, 2017, the United States [37]**							
	I: Fitbit Flex monitoring device + APP^g^ for self-monitoring diet, physical activity + iStart Smart 8-module online education program with in-app messages + text messages (extended intervention only)	SCT	Primary care; mobile phone	Nil	I: In-person demonstrations on how to access Fitbit data and iSmart 8-module online education program	Control group: pedometer, paper food and activity diary, 8 module online program	Nil
**Jensen et al, 2019, the United States [42]**							
	I: Single in-person 50 min MI^h^ session + self-monitoring and adaptive text messages	MI	Primary care	Nil	I: Single in-person MI session with a clinical physiologist doctoral student designed to elicit motivation for change, assess potential barriers to change, and reinforce weight-related behavior change talk and education on Stoplight Eating Plan	Control group: In-person MI session + self-monitoring text messages	1 in-person MI session
**Love-Osborne et al, 2016, the United States [38]**							
	I: Health Educator visits + paper-based self-monitoring log sheet + text messages (TMs^i^ subgroup of intervention group)^j^	MI	School; mobile phone	Nil	I: Up to 8 in-person visits with health educator	Not reported	Not reported
**Mameli et al, 2018, Italy [39]**							
	I: Participants provided a WB^k^ + APP and asked to record the real-time food intake	Not reported	Obesity clinic; mobile phone	Parents	I: In-person training session on how to use WB and APP	Control group: Mediterranean diet advice + instruction to increase physical activity and decrease sedentary time	1 in-person baseline information session
**Nguyen et al, 2012, Australia [40,46]**							
	I: Loozit group program of seven 75-min group sessions during active phase + seven 60-min group sessions during extended phase + ATC^l^ including 32 emails or text messages+ 14 TC^m^ sessions	SCT	Primary care; hospital; mobile phone	Parents	I: Active phase, 7 in-person group sessions with trained dietitians + Extended phase, 7 in-person group sessions + 32 emails or text messages + 14 TC sessions all with trained dietitians	Control group: Loozit Program only	Active phase, 7 in-person group sessions with trained dietitians + Extended phase, 7 in-person group sessions with trained dietitians
**Patrick et al, 2013, the United States [41]**							
	WSMS^n^: W^o^ + text messages	BDM^p^; TTM	Website; mobile phone	Nil	WSMS: communication with health counsellor via text message	Control group: printed educational material + encouraged to attend three 60-min group nutrition sessions at local hospital + monthly mailed tip sheets	Up to 3 in-person group nutrition sessions
	WG^q^: W + 12 90-min group sessions + 24 health coaching calls	BDM; TTM	Website	Parents	WG: In-person group sessions with health counsellor	Not reported	Not reported
	W: wkly check-in emails + monthly mailed tip sheets + access to program website + website tutorials	BDM; TTM	Website	Nil	W: communication with health counsellor via email	Not reported	Not reported

^a^IT: Internet intervention group.

^b^TTM: transtheoretical model.

^c^SCT: social cognitive theory.

^d^sLMP: Simplified Lifestyle Modification Program intervention group.

^e^I: intervention.

^f^In addition, parent text messages and newsletters and increased sports equipment in schools.

^g^APP: smartphone app.

^h^MI: motivational interviewing.

^i^TMs: text messages group.

^j^All participants in the intervention group (TMs and NTMs) were offered text messages during the second school semester.

^k^WB: wristband.

^l^ATC: additional therapeutic contact.

^m^TC: telephone coaching.

^n^WSMS: website + text messages.

^o^W: website only.

^p^BDM: behavioral determinants model.

^q^WG: website + group.

^r^Separate sessions for adolescents and parents or caregivers.

**Table 7:** Text message details of included studies (n=8).

Author, year, country	Duration, n	Direction	Personalized	Content	Intervention (%)^a^
Abraham et al, 2015, China [35]	1/week	Two-way	Semi	Individual diet and exercise goals	Active: 46; Extended: 100
Bagherniya et al, 2018, Iran [36]	1/week	One-way	No	Main goals of program, strategies to overcome barriers	Active: 17
Chen et al, 2017, the United States [37]	2/week	One-way	No	Reinforced adoption and maintenance of healthy lifestyles and weight management practices	Active: 0; Extended: 100
Jensen et al, 2019, the United States [42]	3/day	Two-way	Yes	Self-monitoring text messages: participants sent text messages reporting 4 behaviors; Adaptive text messages: Evidenced-based intervention content delivered in a gain-frame format (indicating what might be gained from adopting healthier behaviors)	Active: 100
Love-Osborne et al, 2016, the United States [38]	2/week	One-way	Yes	One individualized goal-related text message and one reminder to return self-monitoring log sheet	Active: 93
Mameli et al, 2018, Italy [39]	1/week	One-way	Yes	Using previous 7-day WB^b^ and app data, feedback about dietary compliance and quality, energy gap, sedentary time, physical activity and suggestions on how to reach each of 5 goals	Active: 100
Nguyen et al, 2012, Australia [40,46]	1/month	Two-way	Semi	Reinforce key healthy lifestyle principles covered during the active phase and extended phase group sessions	Active: 0; Extended: 60
Patrick et al, 2013, the United States [41]	3/week	Two-way	Yes	Related to weekly challenges and intervention goals	Active: 75

^a^Percentage of intervention delivered by text message was determined by (number of text message contact points ÷ total number of contact points)×100. Each in-person session, website session, or text message is counted as one contact point.

^b^WB: wristband.

### Risk of Bias Assessment

#### Cochrane Risk of Bias Assessment

**Figure 2:**
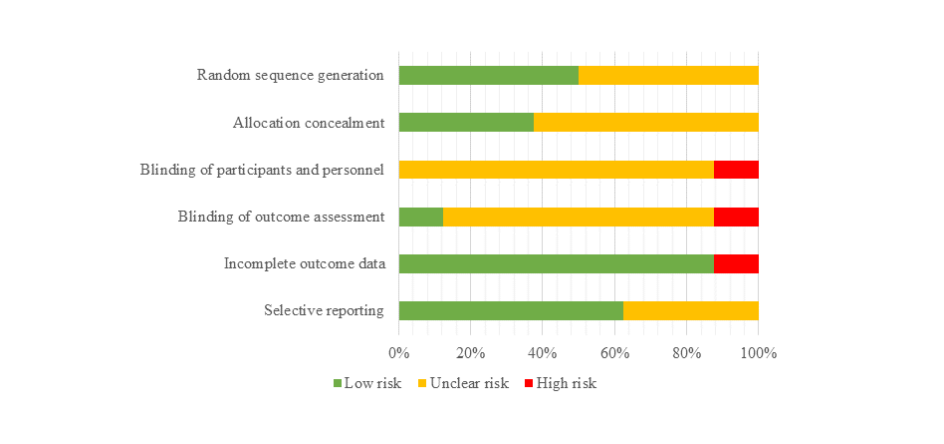
Risk of bias assessment summary.

Overall, the majority of studies were classified as having a low or unclear risk of bias across all main sources of bias (Figure 2 and Multimedia Appendix 6). In all, 5 studies described a random component in the sequence generation process, permitting a judgment of low risk of bias for random sequence generation [35,37,39,40,42]; 5 studies provided insufficient information to determine if the allocation was adequately concealed [36-39,41]; and 7 studies did not provide adequate information to determine if there was blinding of participants and personnel to the intervention [35-38,40-42]. One study stated that there was no blinding and was classified as high risk [39]. One study has blinded outcome assessment [36], 1 study had no blinding of outcome assessment [39], and the remaining studies were unclear [35,37,38,40-42]. A total of 7 studies adequately addressed incomplete data permitting a judgment of low risk of bias [35,37-42]. Overall, 4 studies were rated low risk of bias for selected reporting as they provided a study protocol or trial registration which prespecified all outcome [35-37,40].

#### Grading of Recommendations Assessment, Development, and Evaluation Overall Assessment

The search attempted to identify all interventions for weight loss or management in adolescents delivered via text messages to answer the research question. In the body of studies identified (n=8), there were limitations in study design, variant populations, complex interventions, and potential publication bias resulting in an overall low-quality rating (Table 8).

**Table 8:** Overall assessment of quality in 8 studies (767 participants in total) of weight loss or weight management interventions in adolescents delivered via text message using the Grading of Recommendations Assessment, Development, and Evaluation (GRADE) system.

Category	Rating with reason
Study limitations	−2 quality levels due to very serious limitations
Consistency	No subtraction in levels
Directness	−1 quality level, as the interventions are indirect
Precision	No subtraction of levels due to good precision
Publication bias	−1 quality level, as publication bias cannot be ruled out
Overall quality	Low: our confidence in the effect estimate is limited

#### Study Limitations

All 8 included studies were RCTs. However, within each of the primary sources of bias, there was insufficient information to permit judgments. In all, 3 studies did not provide sufficient detail on the random sequence generation [36,38,41], and 5 studies did not provide adequate detail on the allocation concealment to permit judgment based on selection bias [36-39,41]. A total of 7 studies did not provide sufficient detail of blinding methods for participants and personnel [35-38,40-42], and 1 study did not blind participants and personnel [39]. The blinding of the outcome assessment was unclearly described in 6 studies [35,37,38,40-42], and 1 study did not blind participants or personnel to the outcome assessment [39]. Attrition bias was rated as low risk in 7 studies, and the median attrition rate was below 20% at 3 and 6 months [35,37-41]. However, attrition rates increased above 20% for the 2 studies with 12- and 24-month follow-ups [40,41]. Reporting bias was rated as low risk in 4 studies [35-37,40] and unclear in 4 studies due to insufficient information reported in the publication [38,39,41,42], further limiting the quality of the body of evidence.

#### Consistency

Overall, 4 of the 5 studies found the interventions with text messages did not significantly decrease BMI in intervention participants compared with control participants. Of 5 studies, 3 found the interventions with text messages did not significantly decrease BMI z-score in intervention participants compared with control participants. This result suggests a trend toward no effect. However, no studies reported any subgroup analyses on the effect of text messages only. Therefore, it cannot be determined if text messages were responsible for the intervention effect on BMI or BMI z-score.

#### Directness

Comparisons between the control and intervention arms were direct for the included interventions; variations in the study design, populations, and interventions resulted in the overall body of evidence were indirect. The population of all interventions were adolescents, 10 to 19 years. However, 3 studies recruited all adolescents who were Chinese [35], Chinese American [37], or Persian [36], 2 studies recruited adolescents who were predominately Hispanic [38,41], 1 study recruited adolescents who were predominately white [42], and 2 studies did not report ethnicity of adolescent participants [39,40]. Only 4 studies reported the parents’ education level (range 17%-100% with tertiary education) [35-37,39]. The population is not representative of the broader adolescent population.

This review allowed for the inclusion of studies that used text messages as a component of a multicomponent intervention. Consequently, all studies used text messages in combination with other intervention components. In addition, 2 studies only used text messages during the extended intervention phase [37,40], and no studies evaluated the effectiveness of only the text message intervention delivery with respect to BMI. All studies measured BMI according to standardized procedures. However, the overall evidence is an indirect representation of the effect of weight management or weight loss interventions delivered via text messages on BMI.

#### Precision

A total of 6 studies reported power calculations and 3 studies were pilot or feasibility studies. All power calculations were based on the primary outcome of BMI (measured as BMI kg/m^2^ or BMI z-score). Sample size varied from 40 to 172 participants, with a total of 767 participants, which is considered a sufficient body of evidence for addressing the research question.

#### Publication Bias

A comprehensive search was conducted to ensure that all the available literature was retrieved. This search included major electronic databases, hand searching of reference lists, clinical trial databases and the gray literature, and contacting authors for additional information. Also, as text messages often formed part of larger multicomponent interventions, a conservative approach for full-text screening was adopted to ensure all possible studies were included. However, we may have missed unpublished interventions with insignificant results or negative findings. Therefore, publication bias cannot be ruled out.

## Discussion

### Principal Findings

In this review, we assessed 8 studies that investigated the effectiveness of interventions delivered by text messages for weight management in adolescent populations with overweight or obesity. Of the 8 studies, 7 demonstrated reductions in BMI or BMI z-score in the intervention group compared with the control group at the end of the final follow-up. The effect was only statistically significant in 1 study at 6 months [37]. Significant time by group effects were observed in 3 studies [36,37,40]. The intervention characteristics were heterogenous and multicomponent. However, in the 8 studies, text messages accounted for over 50% of intervention contact points during the active phase, and for the 3 studies with an extended intervention phase, text messages accounted for over 85% of the intervention contact points. Despite text messages accounting for large proportions of the intervention, it cannot be determined if the effects are attributable to the text messages, as no studies utilized a factorial study design and limited process evaluation data to elucidate the characteristics and effects of only text message intervention delivery on BMI outcomes. Also, the overall quality of the body of evidence was rated as low, and this restricts the conclusions that can be drawn. Taking these findings together, this review found that there is limited high-quality evidence for the effectiveness of text messages for weight management in adolescents with overweight or obesity; we, therefore, propose suggestions for improved research design.

### Comparison With Prior Work

There were only 8 RCT studies included in this review, which utilized text messages for weight management intervention delivery in adolescent populations. The small evidence base may indicate that digital research strategies in this area is in its infancy. However, it also well-recognized that investments into research for adolescent obesity prevention have not kept pace with population growth [48]. Many of the successes in childhood obesity prevention over recent times have been the result of targeted investment in interventions benefiting younger children [49,50]. Investing in obesity prevention and management interventions into adolescence is critical to consolidating these successes. A 2019 Cochrane review of 153 RCTs testing the effectiveness of a range of interventions that include diet or physical activity components, or both, designed to prevent obesity in children found only 20% (31/153) of studies targeted adolescents aged 13 to 18 years [10]. Over 50% (85/153) of interventions were targeted at children aged 6 to 12 years. The review concluded that strategies for changing diet or physical activity levels, or both, of children to help prevent them from becoming overweight or obese are effective in making modest reductions in BMI z-score in children aged 6 to 12 years. Similar to the finding of the current systematic review, the Cochrane review found limited evidence for adolescents aged 13 to 18 years, and the diet and physical activity strategies given to them in the studies did not reduce their BMI z-score [10]. Interventions to prevent obesity, including combinations of both physical activity and diet interventions, have so far remained mainly school based and with limited evidence of effective digital intervention strategies. A 2017 systematic review, which focused on digital interventions for improving the diet and physical activity behaviors of adolescents, identified only 4 text message interventions [26]. The 4 interventions were heterogenous and were ineffective at improving physical activity and dietary behaviors [26].

The interventions included in this review were also heterogenous, and in the most part, text messages formed a part of larger, complex, and multicomponent interventions. A systematic review by Moores et al [51], which included all study types of community-based interventions for the treatment of overweight and obesity in adolescents, found that digital technology was utilized in less than half of the 21 included interventions for adolescents, and text messages were only utilized as a part of larger interventions in 4 studies in total. The authors concluded that the inclusion of digital technology did not improve program effectiveness. Similar to the studies included in this review, the interventions were evaluated in traditional RCTs as an aggregation of components. As the traditional RCT evaluates the intervention only as a whole, this evaluation did not enable the isolation of the individual effects of the text messages. Greater consideration in intervention design is required to understand individual user preferences and their engagement with the different intervention components, particularly newer digital technologies. In future studies, this may be achieved through the creation and evaluation of an adaptive intervention using factorial research designs, such as the sequential multiple assessment randomized trial study designs [52]. Post hoc process evaluations were conducted in 5 of the 8 studies included in this review, which allowed some understanding of individual user preferences and how adolescents engaged with and used the text messages. The 3 studies which involved adolescents in the text message development found high adherence rates measured by text message responses and high overall approval ratings [35,37,42].

Adolescents are digital frontrunners and their lack of input into digital technologies to manage their health and well-being is likely to result in ineffective levels of engagement and, subsequently, ineffective interventions [48]. Only 3 of the 8 studies included in this review engaged adolescents in the text message development [35,37,42]. The most comprehensive text message development with adolescents was conducted by Jensen et al [42], who before the start of the intervention, tested the text messages in a 3-month feasibility study and surveyed adolescents about the text message content, timings, and interactivity at monthly intervals and semistructured interviews at the end of the study [24]. This participatory development process allowed the researchers to develop a text message bank that was engaging for participants in the intervention study, with 85% of participants finding the text messages helpful. Similarly, another feasibility study utilized extensive participatory methods with adolescents to develop 300 healthy lifestyle messages and a delivery protocol [53]. The participatory partnership with adolescents allowed researchers to understand adolescents’ preferred text message tone and appreciate adolescents’ sensitivity to the language. For example, messages that used an authoritarian tone were strongly disliked by adolescents. This finding was also highlighted in a process evaluation of a 6-month maintenance text message intervention with adolescents with overweight or obesity [54]. The text message program, which followed an 8-week in-person intervention, was developed without adolescent consultation, and the adolescents described a sense of shame when receiving the text messages and parents perceived the text messages as impersonal. Thus, co-design may increase the likelihood of acceptable text messages and, subsequently, may result in effective engagement in future interventions for adolescents.

Other characteristics related to text message intervention engagement include dose and interactivity. Of the 8 interventions in the current review, 4 had a low dose of text messages of once per month [40] or once per week [35,36,39]. Moreover, only 4 of the included studies utilized two-way text message communication, which allowed participants to have varying levels of interaction with research staff or health professionals [35,40-42]. Jensen et al [42] requested both groups self-report behaviors to the research team via text message [42]. Participants in the intervention group, who also received adaptive text messages (evidenced-based intervention content delivered in a gain-frame format indicating what might be gained from adopting healthier behaviors), demonstrated significantly greater self-reporting adherence compared with the control group (*P*<.01). Comparably, a text messaging program on adolescent reproductive health, which compared one-way text messages with reproductive health information vs interactive text messages with reproductive health quizzes, found interactive text messages increased reproductive knowledge by 24% vs 11% in the one-way text message group [55]. The interactive text message group was instead sent one multiple-choice quiz question each week. When the participant responded, they immediately received a confirmatory text message informing them whether they answered correctly along with the correct answer and additional information, which corresponded to the information provided in the unidirectional text message. Moreover, a momentary ecological assessment using text messages to assess the adolescent’s health information needs found adolescents are willing to use text messaging to report their health information in real time [56]. The research team sent 3 text messages per week asking adolescents if they had any questions about their health and where did they look for the answer. Adolescents responded to 90% of the 3 text messages sent each week by the research team. Adolescents’ most frequently reported questions were about diet and exercise, and adolescents had heightened awareness and health information needs regarding issues related to obesity. Importantly, this sample of adolescents was from a lower socioeconomic status, and text messages showed promise for engaging at-risk and hard-to-reach populations. Future interventions may consider interactive two-way text messages as an intervention delivery strategy for adolescent populations given the demonstration of acceptability in formative research. Unlike other digital technologies, such as online websites and smartphone apps, text messages are inexpensive to develop and to send and receive, and they do not require an internet connection.

To determine the effectiveness of text message interventions for adolescent weight management, consistent outcome measures of body weight are required. Moreover, consistency in secondary outcome measures is essential for behaviors that underlie weight management, such as diet and physical activity behaviors, which have independent effects of their own on health [57]. In the 8 included studies, the primary outcome of interest was weight change measured either using BMI z-score or BMI. BMI z-score is a widely accepted way of assessing a child’s weight status using measures of relative weight adjusted for child age and sex in their country. Of the 5 studies which reported BMI z-score, 2 studies provided insufficient details (groups means at baseline and follow-up time points) to determine an effect size [39,42] and 3 studies only reported BMI [35,36,38]. In growing adolescents, BMI varies with age and sex. As a result, for BMI to be meaningful in adolescents, it must be compared with a reference-standard that accounts for child age and sex [58]. Studies which report outcomes in BMI usually have small effect sizes compared with studies reporting BMI z-scores [51,59]. For example, Chen et al [37] had a BMI effect size of 4.5% at 6 months and BMI z-score effect size of 28.1% at 6 months [37]. This difference in outcome measures limits a quantitative comparison in effect between studies.

### Strengths and Limitations

The strengths of this systematic review include the use of a comprehensive search strategy, developed in conjunction with an experienced librarian. A thorough full-text search was undertaken as text messages often form part of larger multicomponent interventions and all publications in any language were considered. Moreover, a comprehensive quality assessment was undertaken using the Cochrane Risk of Bias tool and GRADE tool. Emails were sent to corresponding authors to obtain missing details and for clarification of text message implementation and outcome measures. Hence, more information was obtained than was published. However, several methodological limitations in this review restrict the conclusions that can be drawn. No studies were identified, which were text message–only interventions. This limited the conclusions that were drawn from the review. The risk of bias and methodological quality of the studies in the review was low and reflected the exploratory nature of text message–delivered interventions as research in this area is in its infancy. Only RCTs were included in the current review, and small uncontrolled studies were excluded, which may bias the results. Moreover, due to heterogenous intervention designs and missing outcome data, the sample size was too small for a meta-analysis of these outcomes to be conducted at each follow-up time point.

### Conclusions

This review demonstrates that there is limited evidence to suggest text messages are an effective tool to deliver interventions for weight management in adolescents with overweight or obesity. Interventions delivered by text message for adolescent weight management were heterogeneous, and the small number of studies indicates research in this area is in its infancy. Although very little high-quality research has been done in the adolescent population, there is high-quality research in adult populations that suggests text messages are an effective tool for the delivery of chronic disease management programs. Further research is required to elucidate the effectiveness and potential impact of only text message interventions on weight and weight-related behaviors in adolescents. Specifically, high-quality studies that evaluate text messages as the only modality of intervention delivery are required.

## Appendix

Multimedia Appendix 1 Preferred Reporting Items for Systematic Reviews and Meta-Analysis checklist.

Multimedia Appendix 2 Full electronic search strategies for each database.

Multimedia Appendix 3 Screenshots of full electronic search strategies for each.

Multimedia Appendix 4 Full text excluded and the reason for exclusion (n=201).

Multimedia Appendix 5 Behaviour change techniques, text message development and process measures in individual included studies (n=8).

Multimedia Appendix 6 The Cochrane Collaboration’s tool for assessing risk of bias in individual included studies (n=8).

## Abbreviations

BCTs: behavioral techniques
CVD: cardiovascular disease
GRADE: Grading of Recommendations Assessment, Development, and Evaluation
mHealth: mobile health
PRISMA: Preferred Reporting Items for Systematic Reviews and Meta-Analysis
RCTs: randomized controlled trials
