# Supplementary material for: Effectiveness of Text Message Interventions for Weight Management in Adolescents: Systematic Review
Source: JMIR Mhealth Uhealth. 2020 May 26;8(5):e15849. doi: 10.2196/15849 (PMC7284408; doi:10.2196/15849)
Supplement: Multimedia Appendix 2 [file mhealth_v8i5e15849_app2.docx]

**Table S1.** Example full electronic search strategy for Medline (18/01/2019)

| **#** | **Search Statement** | **Results** |
| --- | --- | --- |
| 1 | Text Messaging/ | 2116 |
| 2 | telemedicine/ or cell phone/ | 25357 |
| 3 | SMARTPHONE/ | 2539 |
| 4 | Reminder Systems/ | 3155 |
| 5 | ((mobile or cell*) adj phone*).mp. | 10436 |
| 6 | (short messag* service* or SMS).mp. [mp=title, abstract, original title, name of substance word, subject heading word, keyword heading word, protocol supplementary concept word, rare disease supplementary concept word, unique identifier, synonyms] | 4265 |
| 7 | (texting or text messag* or txt or messaging).mp. [mp=title, abstract, original title, name of substance word, subject heading word, keyword heading word, protocol supplementary concept word, rare disease supplementary concept word, unique identifier, synonyms] | 5213 |
| 8 | (mobile health or mhealth or m-health or ehealth or e-health or electronic health).mp. [mp=title, abstract, original title, name of substance word, subject heading word, keyword heading word, protocol supplementary concept word, rare disease supplementary concept word, unique identifier, synonyms] | 28650 |
| 9 | 1 or 2 or 3 or 4 or 5 or 6 or 7 or 8 | 63163 |
| 10 | child nutrition disorders/ or overnutrition/ or obesity/ or obesity, abdominal/ or obesity, metabolically benign/ or obesity, morbid/ or pediatric obesity/ | 193330 |
| 11 | overweight/ or exp obesity/ | 201179 |
| 12 | Body Mass Index/ or Obesity Management/ or Weight Loss/ | 140284 |
| 13 | (obese or obesity or overweight).mp. [mp=title, abstract, original title, name of substance word, subject heading word, keyword heading word, protocol supplementary concept word, rare disease supplementary concept word, unique identifier, synonyms] | 288091 |
| 14 | (weight adj3 (gain or maintenance or maintain* or loss or losing or manag* or reduc*)).mp. | 161968 |
| 15 | (BMI or body mass index or BMI z?score or BMI z-score or BMI percentile).mp. [mp=title, abstract, original title, name of substance word, subject heading word, keyword heading word, protocol supplementary concept word, rare disease supplementary concept word, unique identifier, synonyms] | 217738 |
| 16 | 10 or 11 or 12 or 13 or 14 or 15 | 523000 |
| 17 | 9 and 16 | 1917 |
| 18 | limit 17 to (yr="2005 -Current" and ("all child (0 to 18 years)" or "child (6 to 12 years)" or "adolescent (13 to 18 years)" or "young adult (19 to 24 years)") and (adaptive clinical trial or clinical study or clinical trial, all or clinical trial, phase i or clinical trial, phase ii or clinical trial, phase iii or clinical trial, phase iv or clinical trial protocol or clinical trial protocols as topic or clinical trial or controlled clinical trial or meta analysis or randomized controlled trial or "systematic review" or systematic reviews as topic)) [Limit not valid; records were retained] | 217 |

**Table S2.** Full electronic search strategy for Pre-Medline (18/01/2019)

| **#** | **Search Statement** | **Results** |
| --- | --- | --- |
| 1 | Text Messaging/ | 0 |
| 2 | telemedicine/ or cell phone/ | 0 |
| 3 | SMARTPHONE/ | 0 |
| 4 | Reminder Systems/ | 0 |
| 5 | ((mobile or cell*) adj phone*).mp. | 482 |
| 6 | (short messag* service* or SMS).mp. [mp=title, abstract, original title, name of substance word, subject heading word, keyword heading word, protocol supplementary concept word, rare disease supplementary concept word, unique identifier, synonyms] | 186 |
| 7 | (texting or text messag* or txt or messaging).mp. [mp=title, abstract, original title, name of substance word, subject heading word, keyword heading word, protocol supplementary concept word, rare disease supplementary concept word, unique identifier, synonyms] | 376 |
| 8 | (mobile health or mhealth or m-health or ehealth or e-health or electronic health).mp. [mp=title, abstract, original title, name of substance word, subject heading word, keyword heading word, protocol supplementary concept word, rare disease supplementary concept word, unique identifier, synonyms] | 1372 |
| 9 | 1 or 2 or 3 or 4 or 5 or 6 or 7 or 8 | 2081 |
| 10 | child nutrition disorders/ or overnutrition/ or obesity/ or obesity, abdominal/ or obesity, metabolically benign/ or obesity, morbid/ or pediatric obesity/ | 0 |
| 11 | overweight/ or exp obesity/ | 0 |
| 12 | Body Mass Index/ or Obesity Management/ or Weight Loss/ | 0 |
| 13 | (obese or obesity or overweight).mp. [mp=title, abstract, original title, name of substance word, subject heading word, keyword heading word, protocol supplementary concept word, rare disease supplementary concept word, unique identifier, synonyms] | 6099 |
| 14 | (weight adj3 (gain or maintenance or maintain* or loss or losing or manag* or reduc*)).mp. | 2689 |
| 15 | (BMI or body mass index or BMI z?score or BMI z-score or BMI percentile).mp. [mp=title, abstract, original title, name of substance word, subject heading word, keyword heading word, protocol supplementary concept word, rare disease supplementary concept word, unique identifier, synonyms] | 5051 |
| 16 | 10 or 11 or 12 or 13 or 14 or 15 | 10840 |
| 17 | 9 and 16 | 129 |
| 18 | limit 17 to (yr="2005 -Current" and ("all child (0 to 18 years)" or "child (6 to 12 years)" or "adolescent (13 to 18 years)" or "young adult (19 to 24 years)") and (adaptive clinical trial or clinical study or clinical trial, all or clinical trial, phase i or clinical trial, phase ii or clinical trial, phase iii or clinical trial, phase iv or clinical trial protocol or clinical trial protocols as topic or clinical trial or controlled clinical trial or meta analysis or randomized controlled trial or "systematic review" or systematic reviews as topic)) [Limit not valid; records were retained] | 0 |

**Table S3.** Full electronic search strategy for Embase (18/01/2019)

| **#** | **Search Statement** | **Results** |
| --- | --- | --- |
| 1 | Text Messaging/ | 3874 |
| 2 | telemedicine/ or cell phone/ | 32555 |
| 3 | SMARTPHONE/ | 7397 |
| 4 | Reminder Systems/ | 2179 |
| 5 | ((mobile or cell*) adj phone*).mp. | 18417 |
| 6 | (short messag* service* or SMS).mp. [mp=title, abstract, original title, name of substance word, subject heading word, keyword heading word, protocol supplementary concept word, rare disease supplementary concept word, unique identifier, synonyms] | 7704 |
| 7 | (texting or text messag* or txt or messaging).mp. [mp=title, abstract, original title, name of substance word, subject heading word, keyword heading word, protocol supplementary concept word, rare disease supplementary concept word, unique identifier, synonyms] | 9850 |
| 8 | (mobile health or mhealth or m-health or ehealth or e-health or electronic health).mp. [mp=title, abstract, original title, name of substance word, subject heading word, keyword heading word, protocol supplementary concept word, rare disease supplementary concept word, unique identifier, synonyms] | 31160 |
| 9 | 1 or 2 or 3 or 4 or 5 or 6 or 7 or 8 | 83872 |
| 10 | child nutrition disorders/ or overnutrition/ or obesity/ or obesity, abdominal/ or obesity, metabolically benign/ or obesity, morbid/ or pediatric obesity/ | 408387 |
| 11 | overweight/ or exp obesity/ | 470448 |
| 12 | Body Mass Index/ or Obesity Management/ or Weight Loss/ | 321677 |
| 13 | (obese or obesity or overweight).mp. [mp=title, abstract, original title, name of substance word, subject heading word, keyword heading word, protocol supplementary concept word, rare disease supplementary concept word, unique identifier, synonyms] | 5246450 |
| 14 | (weight adj3 (gain or maintenance or maintain* or loss or losing or manag* or reduc*)).mp. | 348897 |
| 15 | (BMI or body mass index or BMI z?score or BMI z-score or BMI percentile).mp. [mp=title, abstract, original title, name of substance word, subject heading word, keyword heading word, protocol supplementary concept word, rare disease supplementary concept word, unique identifier, synonyms] | 387728 |
| 16 | 10 or 11 or 12 or 13 or 14 or 15 | 1084786 |
| 17 | 9 and 16 | 3986 |
| 18 | limit 17 to (yr="2005 -Current" and ("all child (0 to 18 years)" or "child (6 to 12 years)" or "adolescent (13 to 18 years)" or "young adult (19 to 24 years)") and (adaptive clinical trial or clinical study or clinical trial, all or clinical trial, phase i or clinical trial, phase ii or clinical trial, phase iii or clinical trial, phase iv or clinical trial protocol or clinical trial protocols as topic or clinical trial or controlled clinical trial or meta analysis or randomized controlled trial or "systematic review" or systematic reviews as topic)) [Limit not valid; records were retained] | 927 |

**Table S4.** Full electronic search strategy for CINAHL (21/01/2019)

| **#** | **Search Statement** | **Results** |
| --- | --- | --- |
| 1 | (MH "Text Messaging+") | 1979 |
| 2 | (MH "Telemedicine+") | 10562 |
| 3 | (MH "Cellular Phone+") | 4952 |
| 4 | (MH "Smartphone") | 1781 |
| 5 | (MH "Reminder Systems") | 2417 |
| 6 | "mobile phone*" | 2536 |
| 7 | "cell phone*" | 1198 |
| 8 | "short messag*" | 504 |
| 9 | "short messag* service" | 436 |
| 10 | "sms" | 1098 |
| 11 | "texting" | 549 |
| 12 | "text messag*" | 3073 |
| 13 | "txt" | 110 |
| 14 | "messaging" | 4214 |
| 15 | "mobile health" | 2766 |
| 16 | "mhealth" | 1059 |
| 17 | "m-health" | 134 |
| 18 | "ehealth" | 1586 |
| 19 | "e-health" | 1129 |
| 20 | "electronic health" | 24459 |
| 21 | S1 OR S2 OR S3 OR S4 OR S5 OR S6 OR S7 OR S8 OR S9 OR S10 OR S11 OR S12 OR S13 OR S14 OR S15 OR S16 OR S17 OR S18 OR S19 OR S20 | 50928 |
| 22 | (MH "Child Nutrition Disorders+") | 1517 |
| 23 | "overnutrition" | 269 |
| 24 | (MH "Obesity+") | 82547 |
| 25 | (MH "Obesity, Morbid") | 4107 |
| 26 | (MH "Pediatric Obesity") | 12051 |
| 27 | (MH "Body Mass Index") | 70353 |
| 28 | "Obesity Management" | 404 |
| 29 | (MH "Weight Loss+") | 19652 |
| 30 | "obese" | 31993 |
| 31 | "obesity" | 103689 |
| 32 | "overweight" | 24783 |
| 33 | (MH "Weight Gain+") | 89109 |
| 34 | "weight maintenance" OR (MH "Weight Control") | 7283 |
| 35 | "weight maintain" | 1 |
| 36 | "weight loss" | 29,634 |
| 37 | "weight losing" | 47 |
| 38 | "weight manag*" | 3310 |
| 39 | "weight reduc*" | 4033 |
| 40 | "bmi" | 38,391 |
| 41 | "body mass index" | 94869 |
| 42 | "BMI z-score" | 936 |
| 43 | "BMI z score" | 936 |
| 44 | "BMI percentile" | 431 |
| 45 | S22 OR S23 OR S24 OR S25 OR S26 OR S27 OR S28 OR S29 OR S30 OR S31 OR S32 OR S33 OR S34 OR S35 OR S36 OR S37 OR S38 OR S39 OR S40 OR S41 OR S42 OR S43 OR S44 | 203347 |
| 46 | S21 AND S45 | 1371 |
| 47 | S21 AND S45  Limiters - Published Date: 20050101-20191231 | 1353 |
| 48 | S21 AND S45  Limiters - Published Date: 20050101-20191231  Narrow by SubjectAge: - all child | 387 |
| 49 | S21 AND S45  Limiters - Published Date: 20050101-20191231  Narrow by SubjectAge: - adolescent: 13-18 years | 266 |
| 50 | S21 AND S45  Limiters - Published Date: 20050101-20191231  Narrow by SubjectAge: - child: 6-12 years | 208 |
| 51 | S48 OR S49 OR S50 | 387 |

**Table S5.** Full electronic search strategy for AMED (18/01/2019)

| **#** | **Search Statement** | **Results** |
| --- | --- | --- |
| 1 | Text Messaging/ | 0 |
| 2 | telemedicine/ or cell phone/ | 769 |
| 3 | SMARTPHONE/ | 0 |
| 4 | Reminder Systems/ | 0 |
| 5 | ((mobile or cell*) adj phone*).mp. | 125 |
| 6 | (short messag* service* or SMS).mp. [mp=title, abstract, original title, name of substance word, subject heading word, keyword heading word, protocol supplementary concept word, rare disease supplementary concept word, unique identifier, synonyms] | 58 |
| 7 | (texting or text messag* or txt or messaging).mp. [mp=title, abstract, original title, name of substance word, subject heading word, keyword heading word, protocol supplementary concept word, rare disease supplementary concept word, unique identifier, synonyms] | 93 |
| 8 | (mobile health or mhealth or m-health or ehealth or e-health or electronic health).mp. [mp=title, abstract, original title, name of substance word, subject heading word, keyword heading word, protocol supplementary concept word, rare disease supplementary concept word, unique identifier, synonyms] | 119 |
| 9 | 1 or 2 or 3 or 4 or 5 or 6 or 7 or 8 | 1028 |
| 10 | child nutrition disorders/ or overnutrition/ or obesity/ or obesity, abdominal/ or obesity, metabolically benign/ or obesity, morbid/ or pediatric obesity/ | 1825 |
| 11 | overweight/ or exp obesity/ | 1889 |
| 12 | Body Mass Index/ or Obesity Management/ or Weight Loss/ | 826 |
| 13 | (obese or obesity or overweight).mp. [mp=title, abstract, original title, name of substance word, subject heading word, keyword heading word, protocol supplementary concept word, rare disease supplementary concept word, unique identifier, synonyms] | 2671 |
| 14 | (weight adj3 (gain or maintenance or maintain* or loss or losing or manag* or reduc*)).mp. | 1591 |
| 15 | (BMI or body mass index or BMI z?score or BMI z-score or BMI percentile).mp. [mp=title, abstract, original title, name of substance word, subject heading word, keyword heading word, protocol supplementary concept word, rare disease supplementary concept word, unique identifier, synonyms] | 2059 |
| 16 | 10 or 11 or 12 or 13 or 14 or 15 | 5033 |
| 17 | 9 and 16 | 16 |

**Table S6.** Full electronic search strategy for Cochrane Central Register of Controlled Trials (21/01/2019)

| **#** | **Search Statement** | **Results** |
| --- | --- | --- |
| 1 | Text Messaging/ | 574 |
| 2 | telemedicine/ or cell phone/ | 2026 |
| 3 | SMARTPHONE/ | 190 |
| 4 | Reminder Systems/ | 804 |
| 5 | ((mobile or cell*) adj phone*).mp. | 2018 |
| 6 | (short messag* service* or SMS).mp. [mp=title, abstract, original title, name of substance word, subject heading word, keyword heading word, protocol supplementary concept word, rare disease supplementary concept word, unique identifier, synonyms] | 1216 |
| 7 | (texting or text messag* or txt or messaging).mp. [mp=title, abstract, original title, name of substance word, subject heading word, keyword heading word, protocol supplementary concept word, rare disease supplementary concept word, unique identifier, synonyms] | 2709 |
| 8 | (mobile health or mhealth or m-health or ehealth or e-health or electronic health).mp. [mp=title, abstract, original title, name of substance word, subject heading word, keyword heading word, protocol supplementary concept word, rare disease supplementary concept word, unique identifier, synonyms] | 2475 |
| 9 | 1 or 2 or 3 or 4 or 5 or 6 or 7 or 8 | 8339 |
| 10 | child nutrition disorders/ or overnutrition/ or obesity/ or obesity, abdominal/ or obesity, metabolically benign/ or obesity, morbid/ or pediatric obesity/ | 11618 |
| 11 | overweight/ or exp obesity/ | 13570 |
| 12 | Body Mass Index/ or Obesity Management/ or Weight Loss/ | 12841 |
| 13 | (obese or obesity or overweight).mp. [mp=title, abstract, original title, name of substance word, subject heading word, keyword heading word, protocol supplementary concept word, rare disease supplementary concept word, unique identifier, synonyms] | 32847 |
| 14 | (weight adj3 (gain or maintenance or maintain* or loss or losing or manag* or reduc*)).mp. | 27466 |
| 15 | (BMI or body mass index or BMI z?score or BMI z-score or BMI percentile).mp. [mp=title, abstract, original title, name of substance word, subject heading word, keyword heading word, protocol supplementary concept word, rare disease supplementary concept word, unique identifier, synonyms] | 38620 |
| 16 | 10 or 11 or 12 or 13 or 14 or 15 | 69324 |
| 17 | 9 and 16 | 877 |
| 18 | limit 17 to (yr="2005 -Current" and ("all child (0 to 18 years)" or "child (6 to 12 years)" or "adolescent (13 to 18 years)" or "young adult (19 to 24 years)") and (adaptive clinical trial or clinical study or clinical trial, all or clinical trial, phase i or clinical trial, phase ii or clinical trial, phase iii or clinical trial, phase iv or clinical trial protocol or clinical trial protocols as topic or clinical trial or controlled clinical trial or meta analysis or randomized controlled trial or "systematic review" or systematic reviews as topic)) [Limit not valid; records were retained] | 378 |

**Table S7.** Full electronic search strategy for Cochrane (21/01/2019)

| **#** | **Search Statement** | **Results** |
| --- | --- | --- |
| 1 | Text Messaging/ | 0 |
| 2 | telemedicine/ or cell phone/ | 0 |
| 3 | SMARTPHONE/ | 0 |
| 4 | Reminder Systems/ | 0 |
| 5 | ((mobile or cell*) adj phone*).mp. | 147 |
| 6 | (short messag* service* or SMS).mp. [mp=title, abstract, original title, name of substance word, subject heading word, keyword heading word, protocol supplementary concept word, rare disease supplementary concept word, unique identifier, synonyms] | 93 |
| 7 | (texting or text messag* or txt or messaging).mp. [mp=title, abstract, original title, name of substance word, subject heading word, keyword heading word, protocol supplementary concept word, rare disease supplementary concept word, unique identifier, synonyms] | 146 |
| 8 | (mobile health or mhealth or m-health or ehealth or e-health or electronic health).mp. [mp=title, abstract, original title, name of substance word, subject heading word, keyword heading word, protocol supplementary concept word, rare disease supplementary concept word, unique identifier, synonyms] | 126 |
| 9 | 1 or 2 or 3 or 4 or 5 or 6 or 7 or 8 | 293 |
| 10 | child nutrition disorders/ or overnutrition/ or obesity/ or obesity, abdominal/ or obesity, metabolically benign/ or obesity, morbid/ or pediatric obesity/ | 0 |
| 11 | overweight/ or exp obesity/ | 0 |
| 12 | Body Mass Index/ or Obesity Management/ or Weight Loss/ | 0 |
| 13 | (obese or obesity or overweight).mp. [mp=title, abstract, original title, name of substance word, subject heading word, keyword heading word, protocol supplementary concept word, rare disease supplementary concept word, unique identifier, synonyms] | 955 |
| 14 | (weight adj3 (gain or maintenance or maintain* or loss or losing or manag* or reduc*)).mp. | 1452 |
| 15 | (BMI or body mass index or BMI z?score or BMI z-score or BMI percentile).mp. [mp=title, abstract, original title, name of substance word, subject heading word, keyword heading word, protocol supplementary concept word, rare disease supplementary concept word, unique identifier, synonyms] | 820 |
| 16 | 10 or 11 or 12 or 13 or 14 or 15 | 2291 |
| 17 | 9 and 16 | 88 |
| 18 | limit 17 to (yr="2005 -Current" and ("all child (0 to 18 years)" or "child (6 to 12 years)" or "adolescent (13 to 18 years)" or "young adult (19 to 24 years)") and (adaptive clinical trial or clinical study or clinical trial, all or clinical trial, phase i or clinical trial, phase ii or clinical trial, phase iii or clinical trial, phase iv or clinical trial protocol or clinical trial protocols as topic or clinical trial or controlled clinical trial or meta analysis or randomized controlled trial or "systematic review" or systematic reviews as topic)) [Limit not valid; records were retained] | 73 |

**Table S8.** Full electronic search strategy for Informit (21/01/2019)

(((Text messag*) OR telemedicine OR (cellular phone) OR smartphone OR (reminder system) OR (mobile phone) OR (cellular phone) OR (short messag*) OR (short messag* service) OR sms OR texting OR (text messag*) OR Txt OR Messaging OR mobile health OR mhealth OR m-health OR ehealth OR e-health OR (electronic health))) AND (((Obesity Management) obese OR obesity OR overweight OR (weight maintenance) OR (weight maintain) OR (weight loss) OR (weight losing) OR (weight manag*) OR (weight reduc*) OR bmi OR (body mass index) OR (BMI z-score) OR (BMI z score) OR (BMI percentile))) AND ((child OR children OR adolescent OR adolescence OR (young adult) OR (young person) OR teenager OR teen OR (early life)))

**Table S9.** Full electronic search strategy for Scopus (21/01/2019)

( TITLE-ABS-KEY ( ( "Text messag*" ) OR telemedicine OR ( "cellular phone" ) OR smartphone OR ( "reminder system" ) OR ( "mobile phone" ) OR ( "cellular phone" ) OR ( "short messag*" ) OR ( "short messag* service" ) OR sms OR texting OR ( "text messag*" ) OR txt OR messaging OR ( "mobile health" ) OR mhealth OR ( "m-health" ) OR ehealth OR ( "e-health" ) OR ( "electronic health" ) ) ) AND ( TITLE-ABS-KEY ( ( "Obesity Management" ) obese OR obesity OR overweight OR ( "weight maintenance" ) OR ( "weight maintain" ) OR ( "weight loss" ) OR ( "weight losing" ) OR ( "weight manag*" ) OR ( "weight reduc*" ) OR bmi OR ( "body mass index" ) OR ( "BMI z-score" ) OR ( "BMI z score" ) OR ( "BMI percentile" ) ) )

**Table S10.** Full electronic search strategy for Web of Science (21/01/2019)

((((TS= ((Text messag*) OR telemedicine OR (cellular phone) OR smartphone OR (reminder system) OR (mobile phone) OR (cellular phone) OR (short messag*) OR (short messag* service) OR sms OR texting OR (text messag*) OR Txt OR Messaging OR mobile health OR mhealth OR m-health OR ehealth OR e-health OR (electronic health))) OR (TI= ((Text messag*) OR telemedicine OR (cellular phone) OR smartphone OR (reminder system) OR (mobile phone) OR (cellular phone) OR (short messag*) OR (short messag* service) OR sms OR texting OR (text messag*) OR Txt OR Messaging OR mobile health OR mhealth OR m-health OR ehealth OR e-health OR (electronic health)))) AND ((TS= ((Obesity Management) obese OR obesity OR overweight OR (weight maintenance) OR (weight maintain) OR (weight loss) OR (weight losing) OR (weight manag*) OR (weight reduc*) OR bmi OR (body mass index) OR (BMI z-score) OR (BMI z score) OR (BMI percentile))) OR (TI= ((Obesity Management) obese OR obesity OR overweight OR (weight maintenance) OR (weight maintain) OR (weight loss) OR (weight losing) OR (weight manag*) OR (weight reduc*) OR bmi OR (body mass index) OR (BMI z-score) OR (BMI z score) OR (BMI percentile)))) AND ((TS= (child OR children OR adolescent OR adolescence OR (young adult) OR (young person) OR teenager OR teen OR (early life))) OR (TI= (child OR children OR adolescent OR adolescence OR (young adult) OR (young person) OR teenager OR teen OR (early life)))))) AND DOCUMENT TYPES: (Article OR Abstract of Published Item OR Review)

Refined by: WEB OF SCIENCE CATEGORIES: ( PUBLIC ENVIRONMENTAL OCCUPATIONAL HEALTH OR NUTRITION DIETETICS OR PEDIATRICS OR HEALTH CARE SCIENCES SERVICES OR MEDICINE GENERAL INTERNAL OR ENDOCRINOLOGY METABOLISM OR MEDICAL INFORMATICS OR NURSING OR HEALTH POLICY SERVICES OR PSYCHOLOGY DEVELOPMENTAL OR SPORT SCIENCES OR MEDICINE RESEARCH EXPERIMENTAL OR BEHAVIORAL SCIENCES OR PHYSIOLOGY OR COMMUNICATION OR EDUCATION EDUCATIONAL RESEARCH OR MULTIDISCIPLINARY SCIENCES OR TELECOMMUNICATIONS OR INFORMATION SCIENCE LIBRARY SCIENCE OR PRIMARY HEALTH CARE OR FAMILY STUDIES OR CARDIAC CARDIOVASCULAR SYSTEMS )

Indexes=SCI-EXPANDED, SSCI, A&HCI, CPCI-S, CPCI-SSH, ESCI, CCR-EXPANDED, IC Timespan=2005-2019
