# Supplementary material for: Effectiveness of Text Message Interventions for Weight Management in Adolescents: Systematic Review
Source: JMIR Mhealth Uhealth. 2020 May 26;8(5):e15849. doi: 10.2196/15849 (PMC7284408; doi:10.2196/15849)
Supplement: Multimedia Appendix 3 [file mhealth_v8i5e15849_app3.docx]

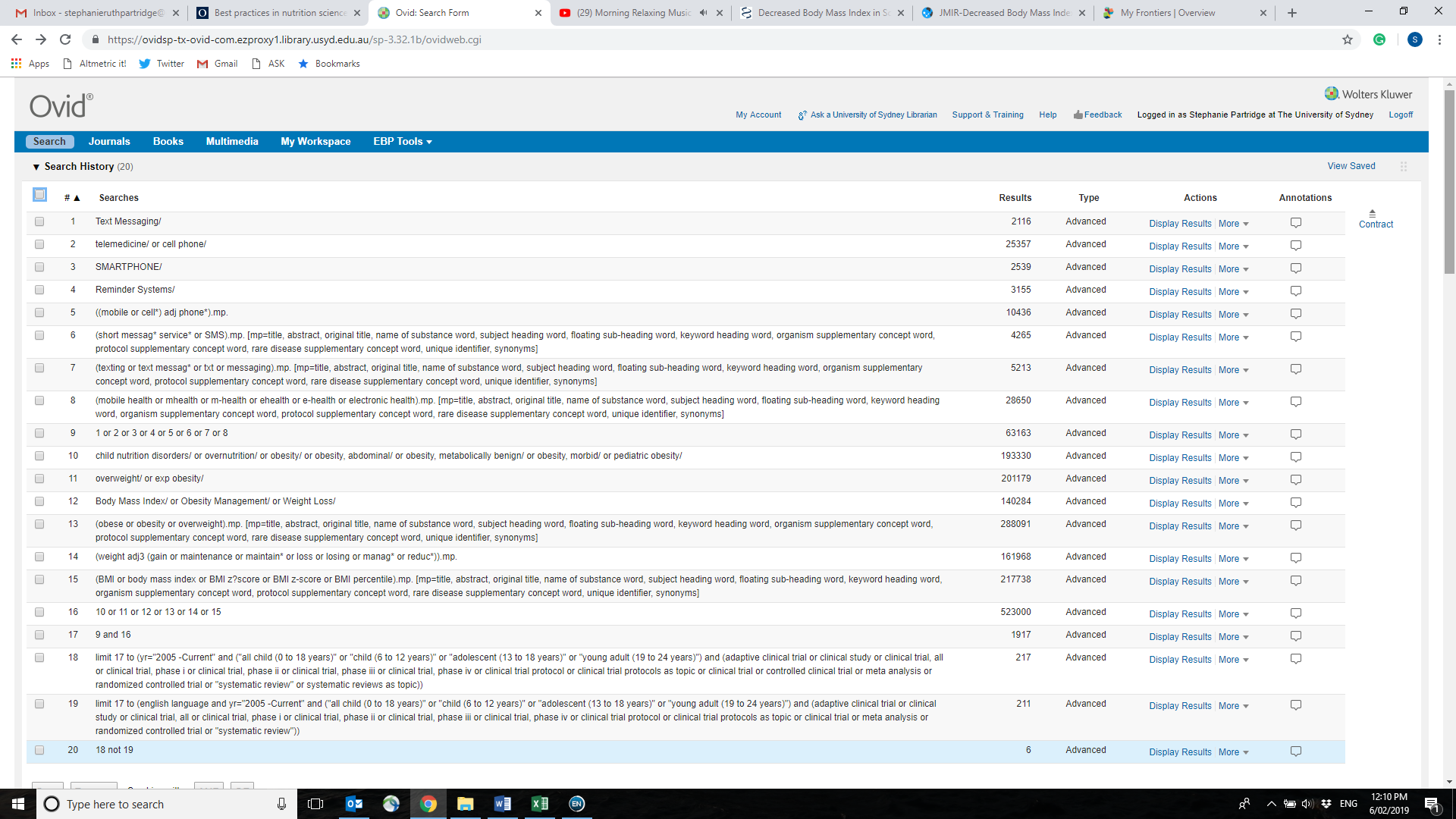


**Figure S1.** Screenshot of full electronic search strategy for Medline (06/02/2019)


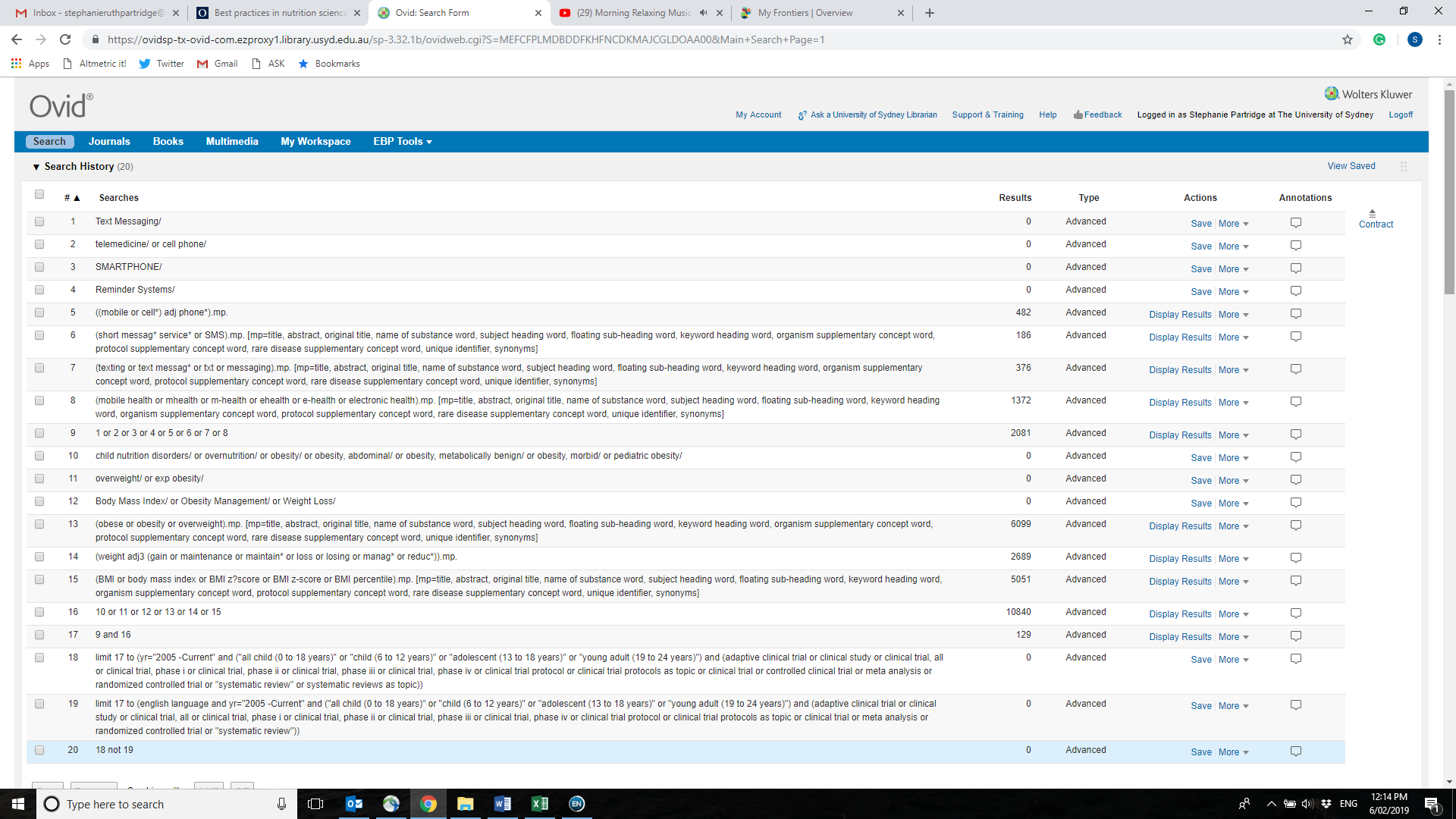


**Figure S2.** Screenshot of full electronic search strategy for Pre-Medline (06/02/2019)


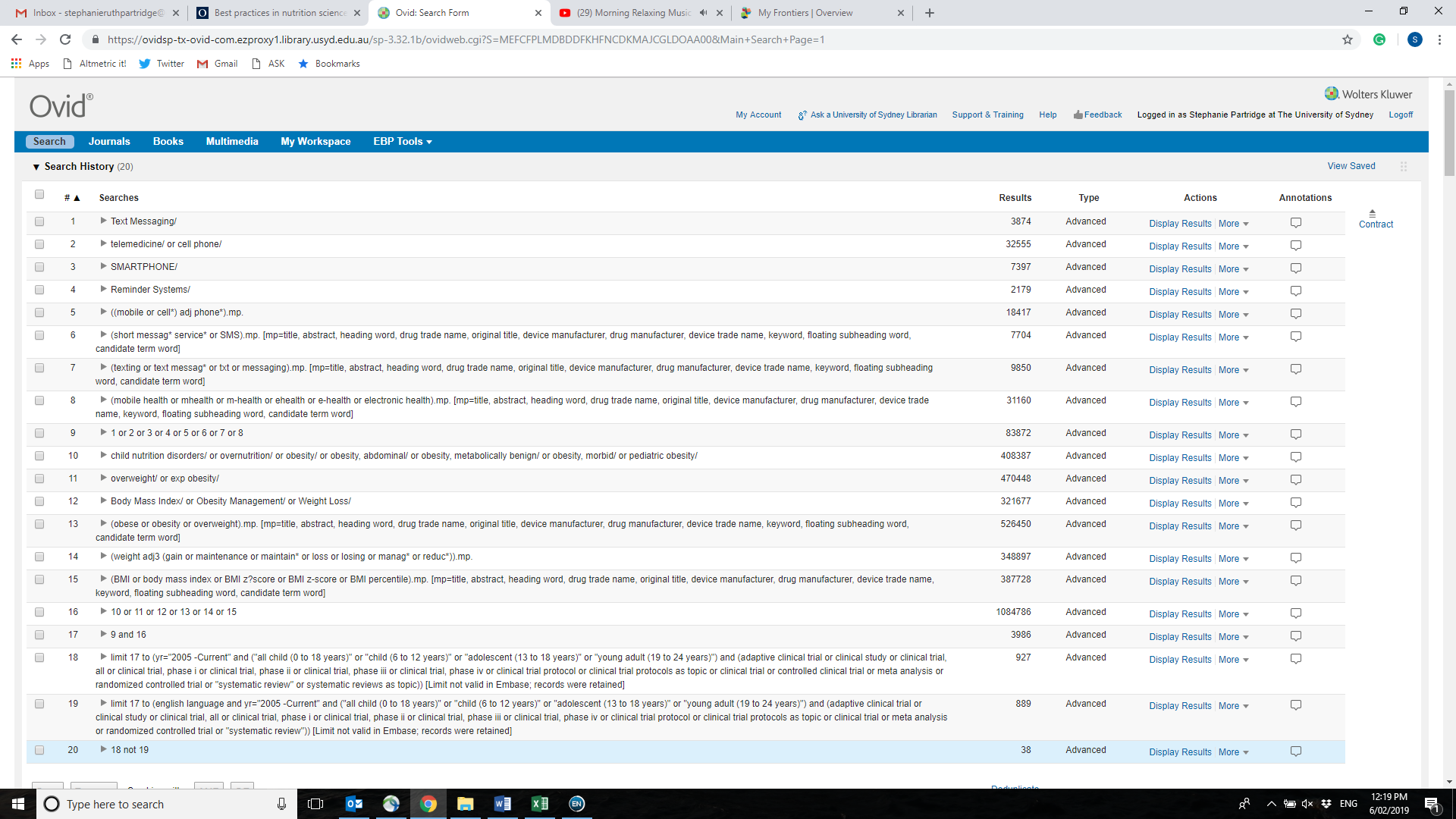


**Figure S3.** Screenshot of full electronic search strategy for Embase (06/02/2019)

**
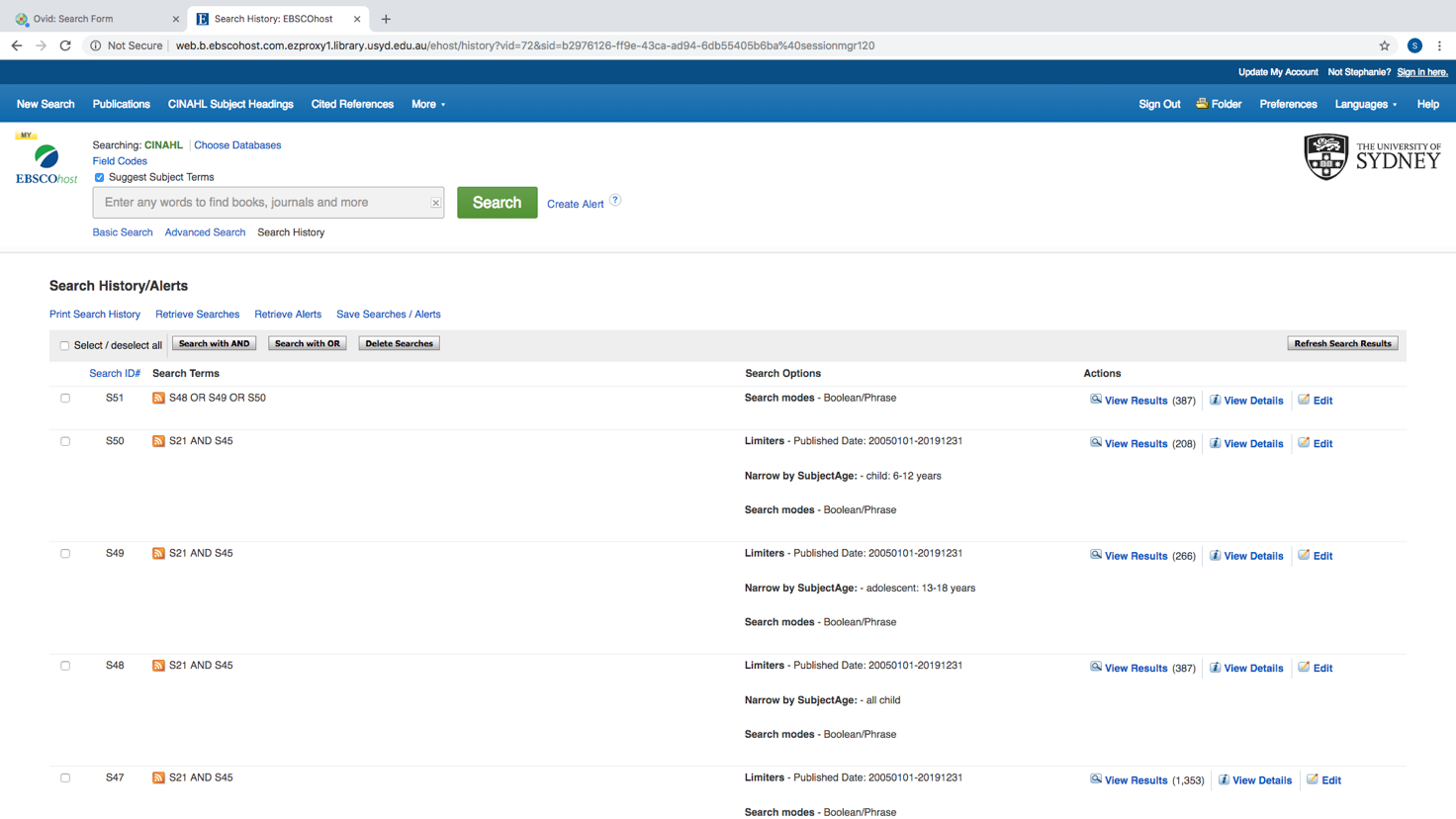
**

**
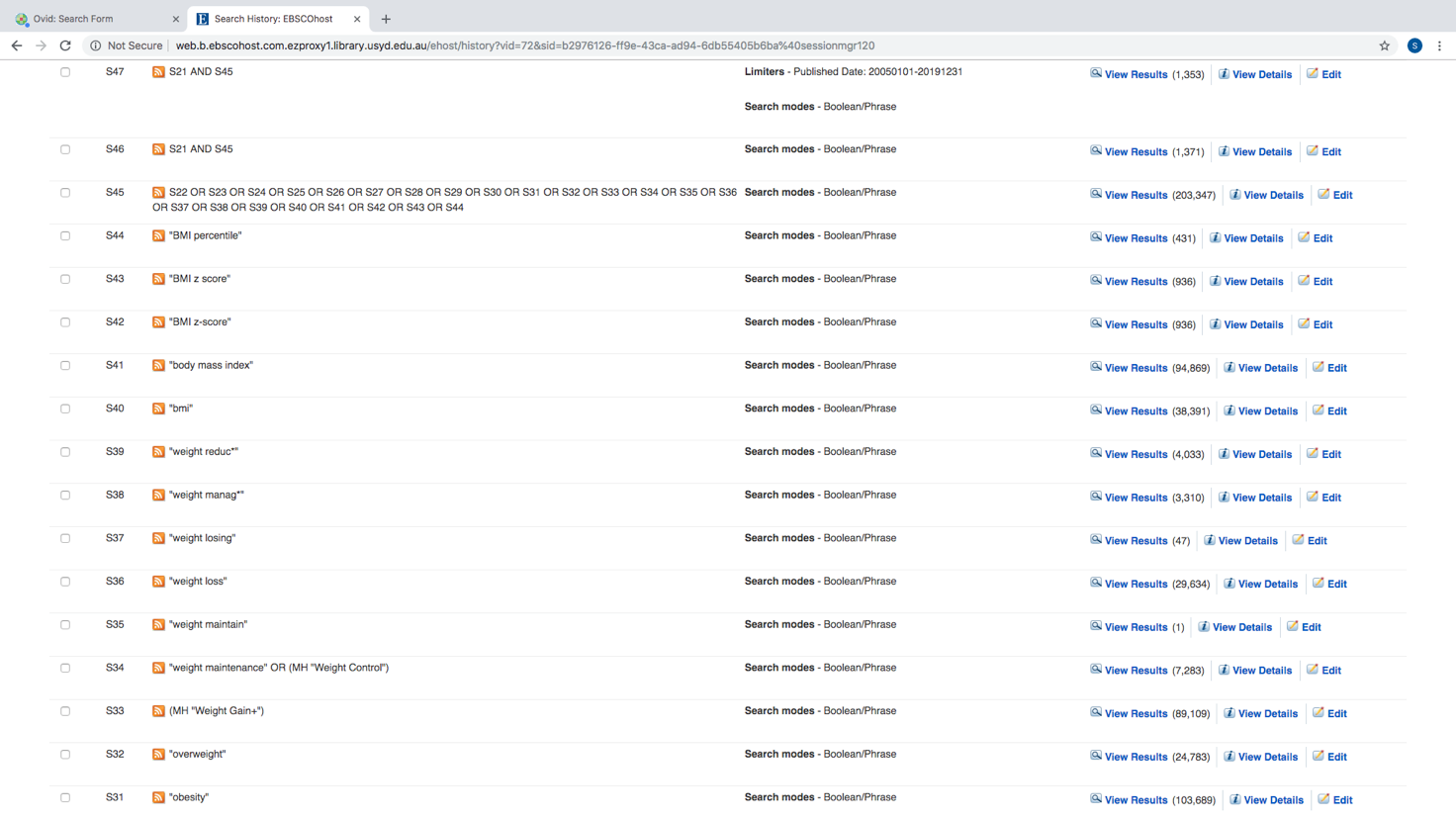
**

**
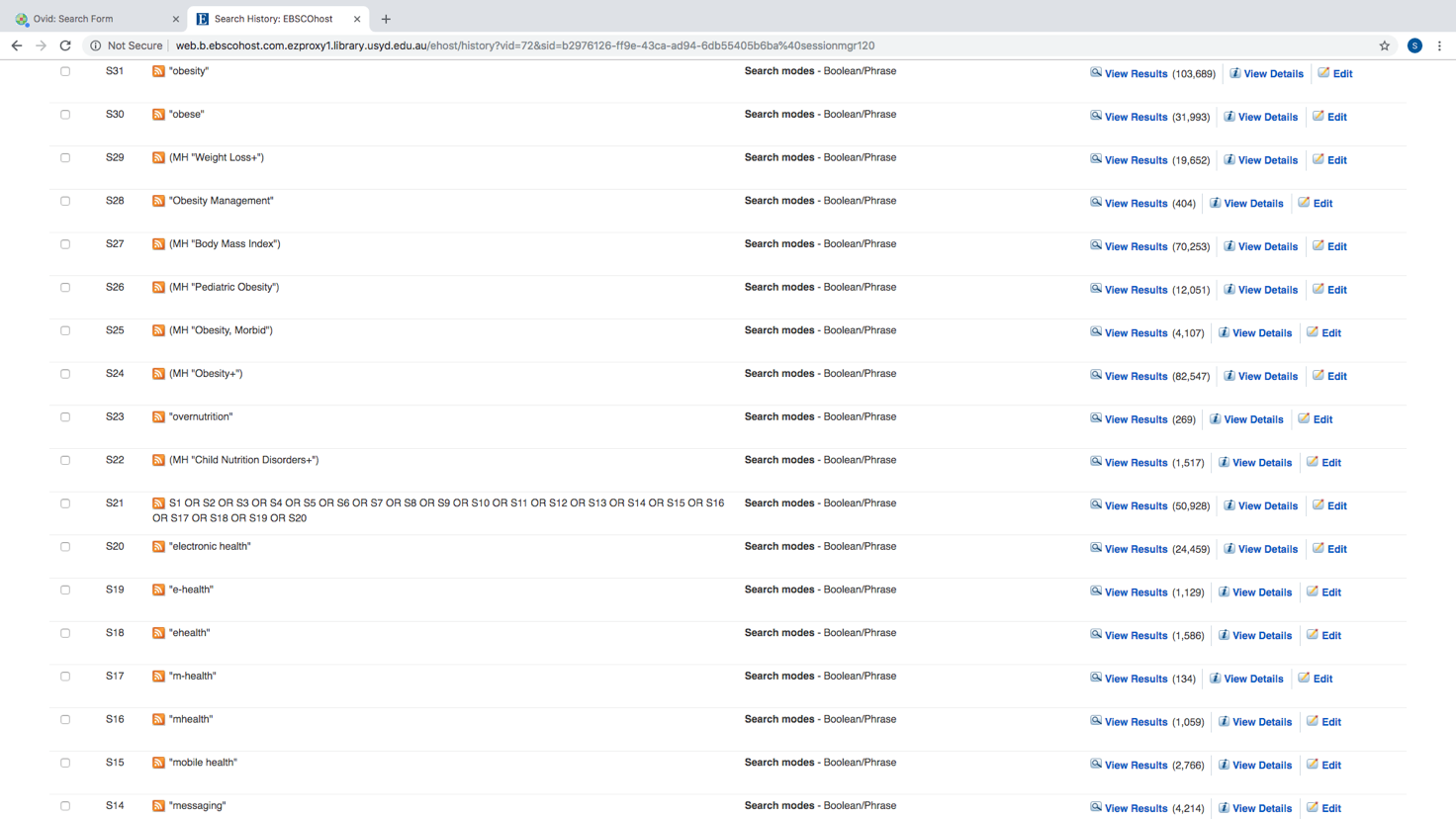
**

**
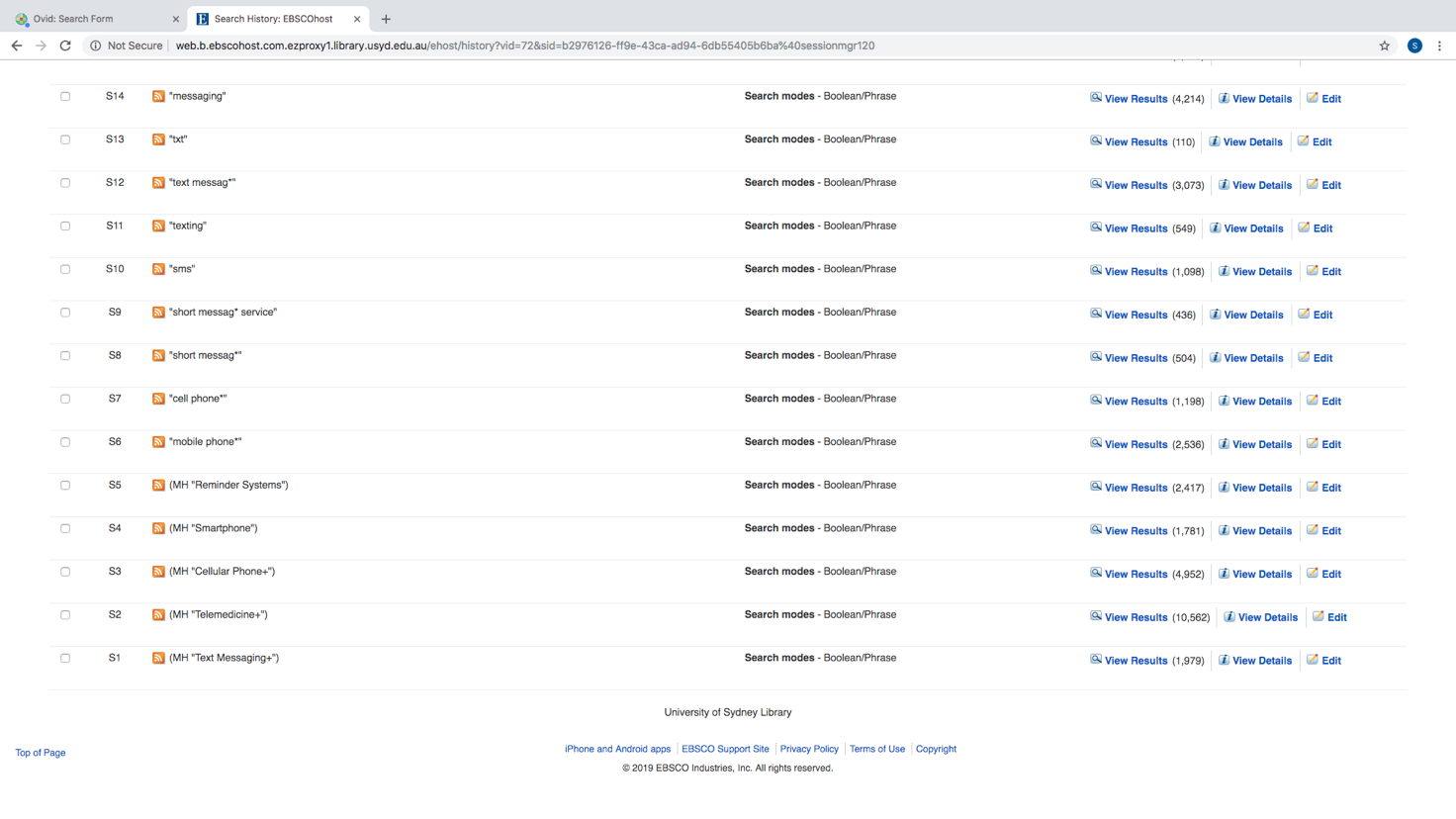
**

**Figure S4.** Screenshot of full electronic search strategy for CINAHL (18/01/2019)

**
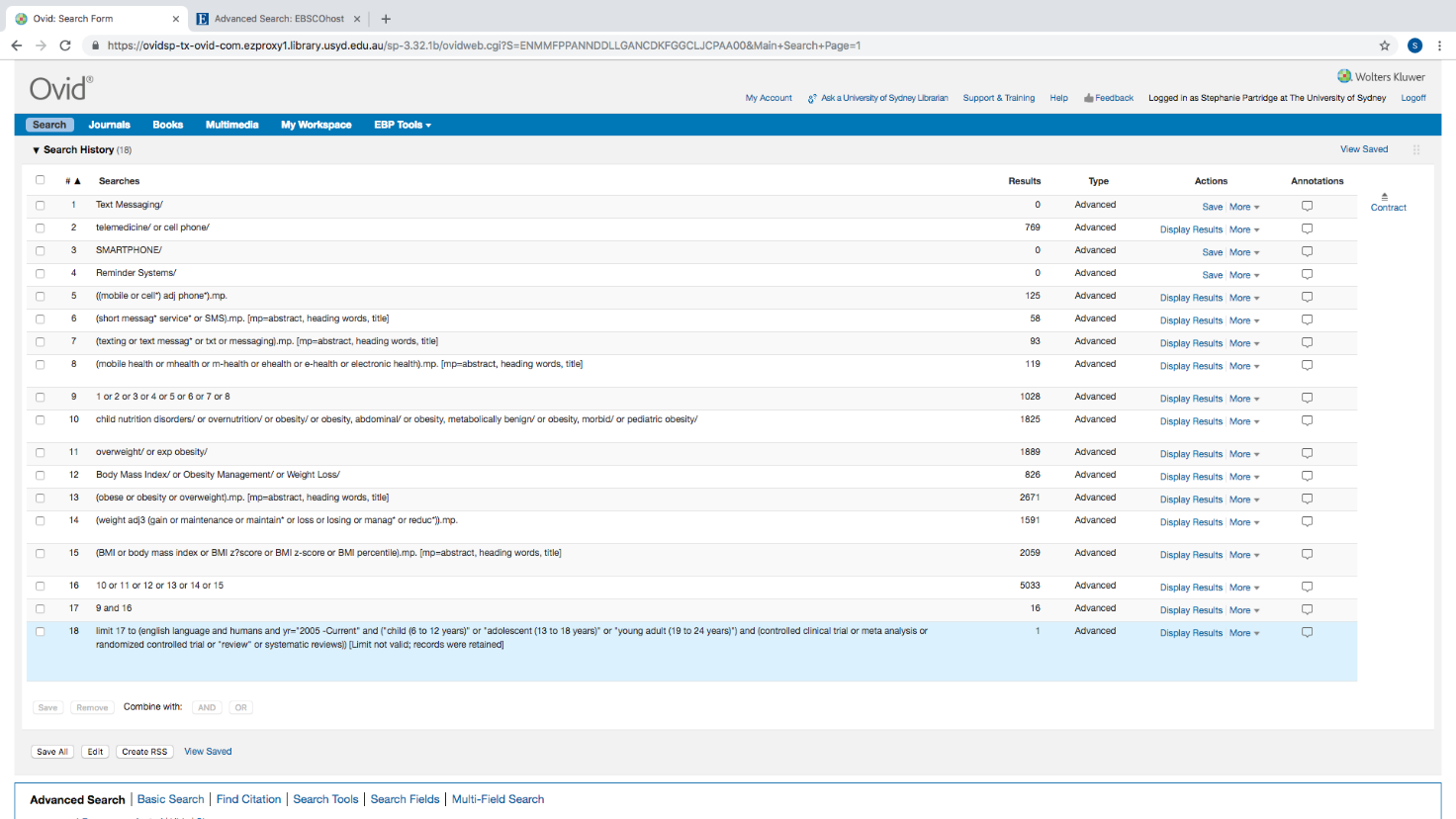
**

**Figure S5.** Screenshot of full electronic search strategy for AMED (18/01/2019)


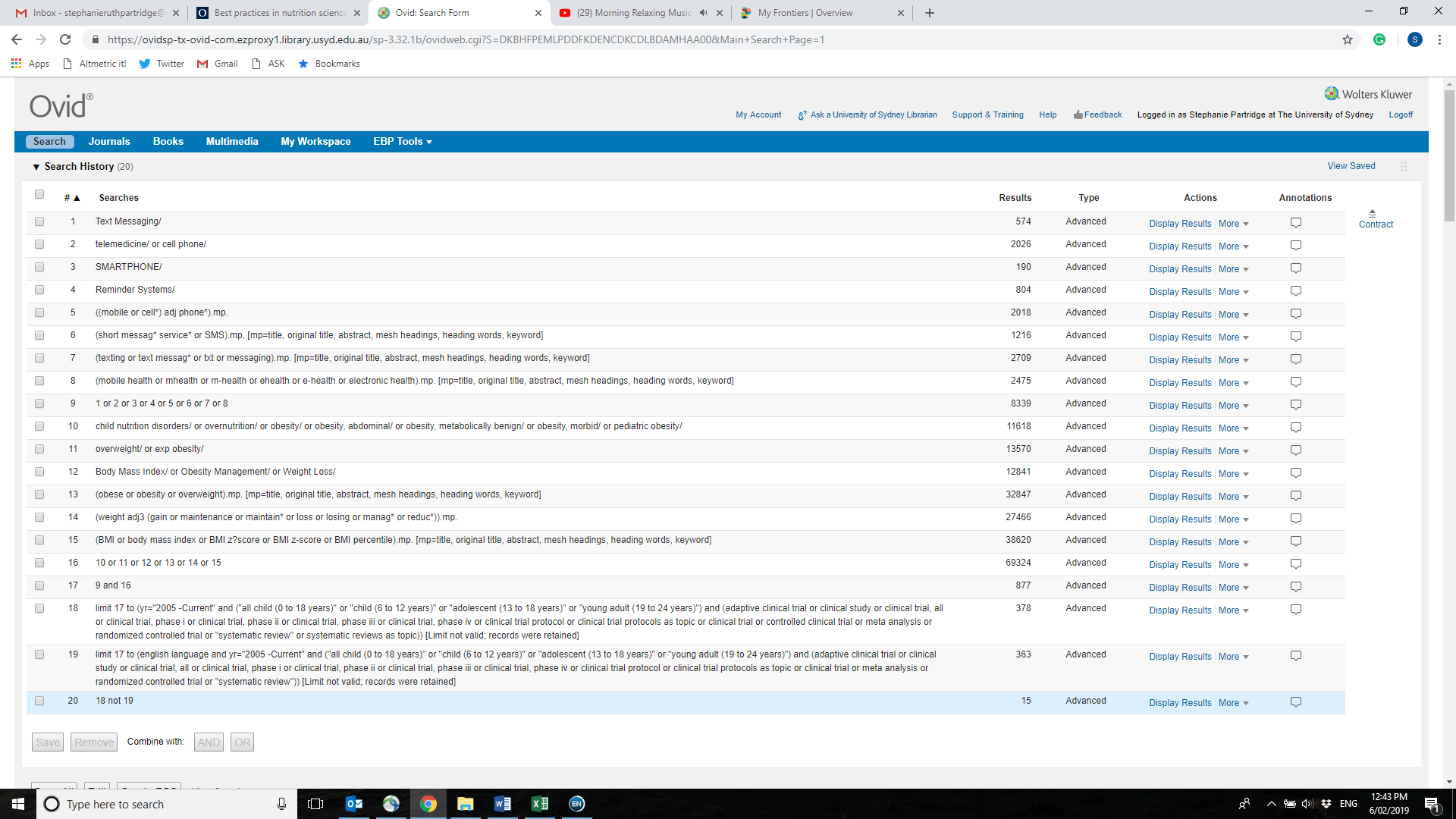


**Figure S6.** Screenshot of full electronic search strategy for Cochrane Central Register of Controlled (05/02/2019)


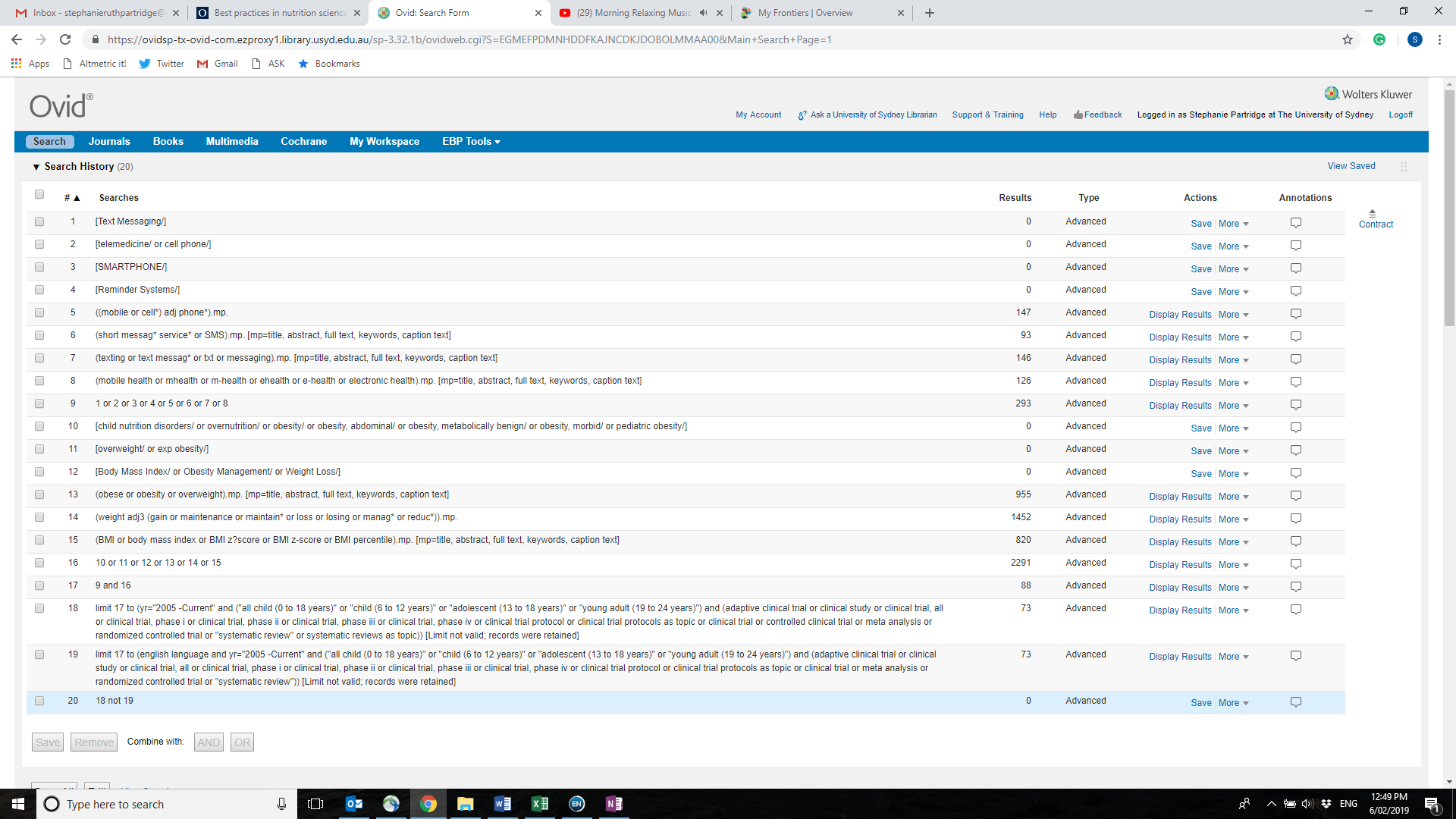


**Figure S7.** Screenshot of full electronic search strategy for Cochrane (05/02/2019)

**
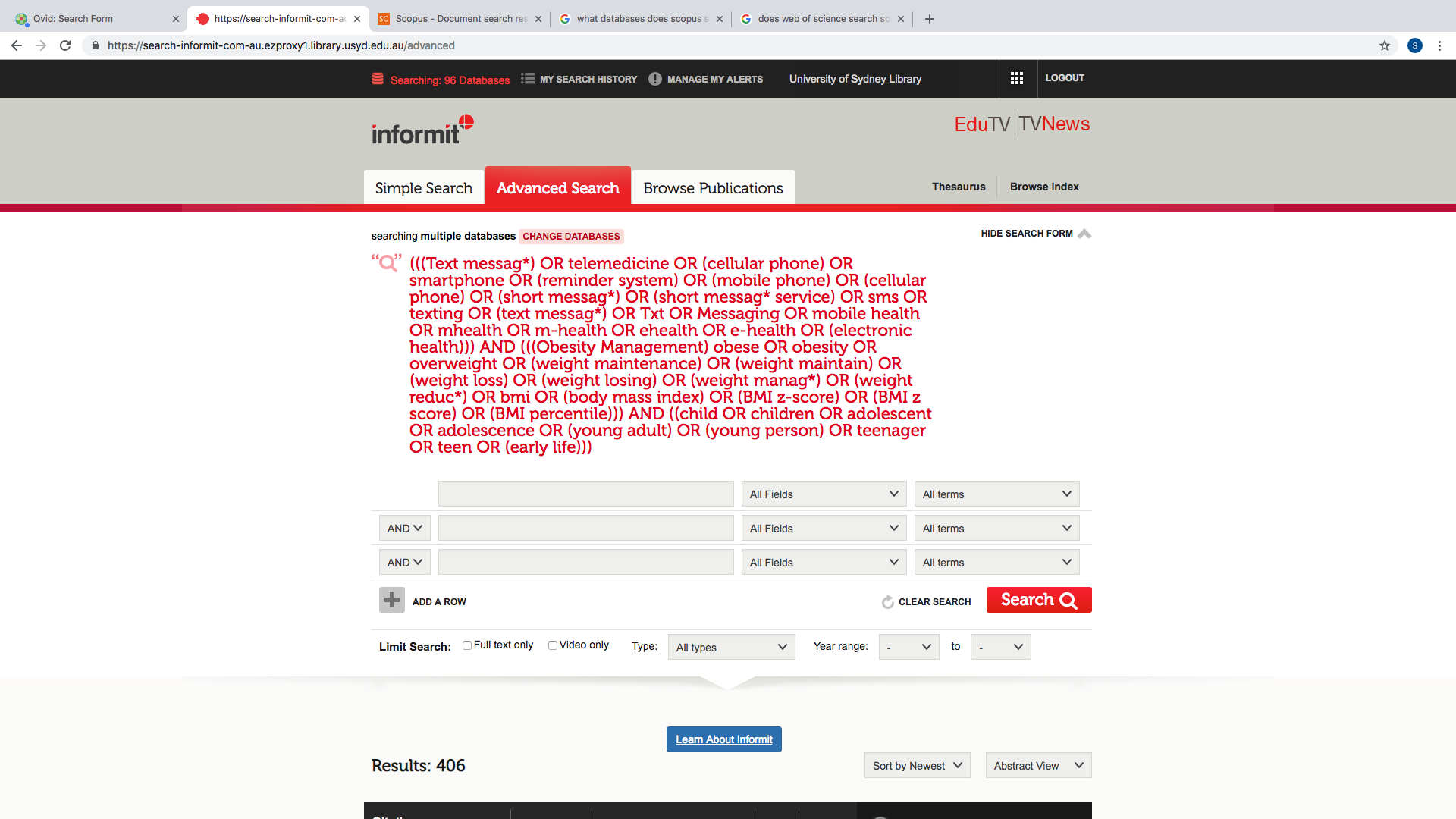
**

**Figure S8.** Screenshot of full electronic search strategy for Informit (21/01/2019)

**
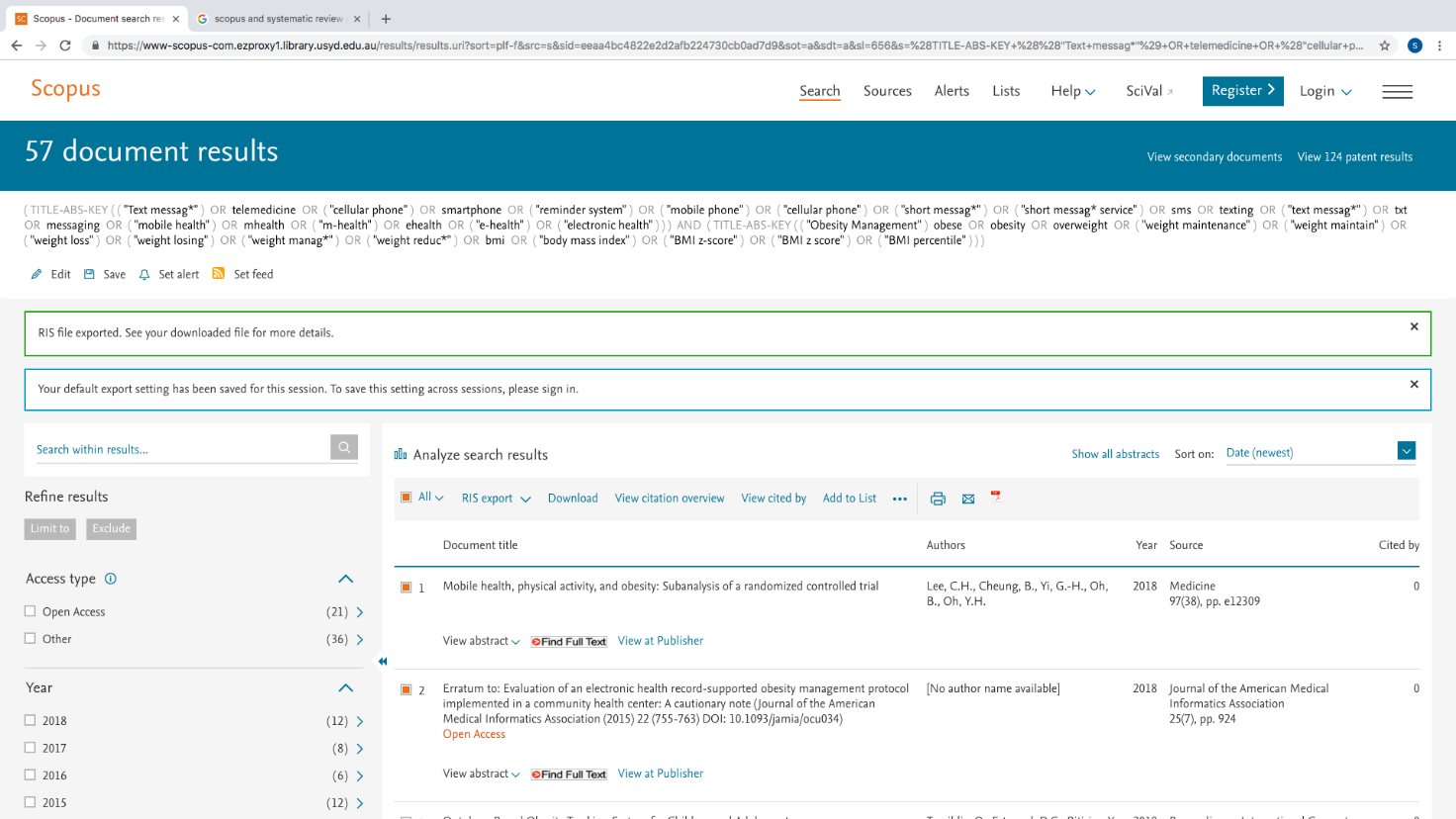
**

**Figure S9.** Screenshot of full electronic search strategy for Scopus (21/01/2019)


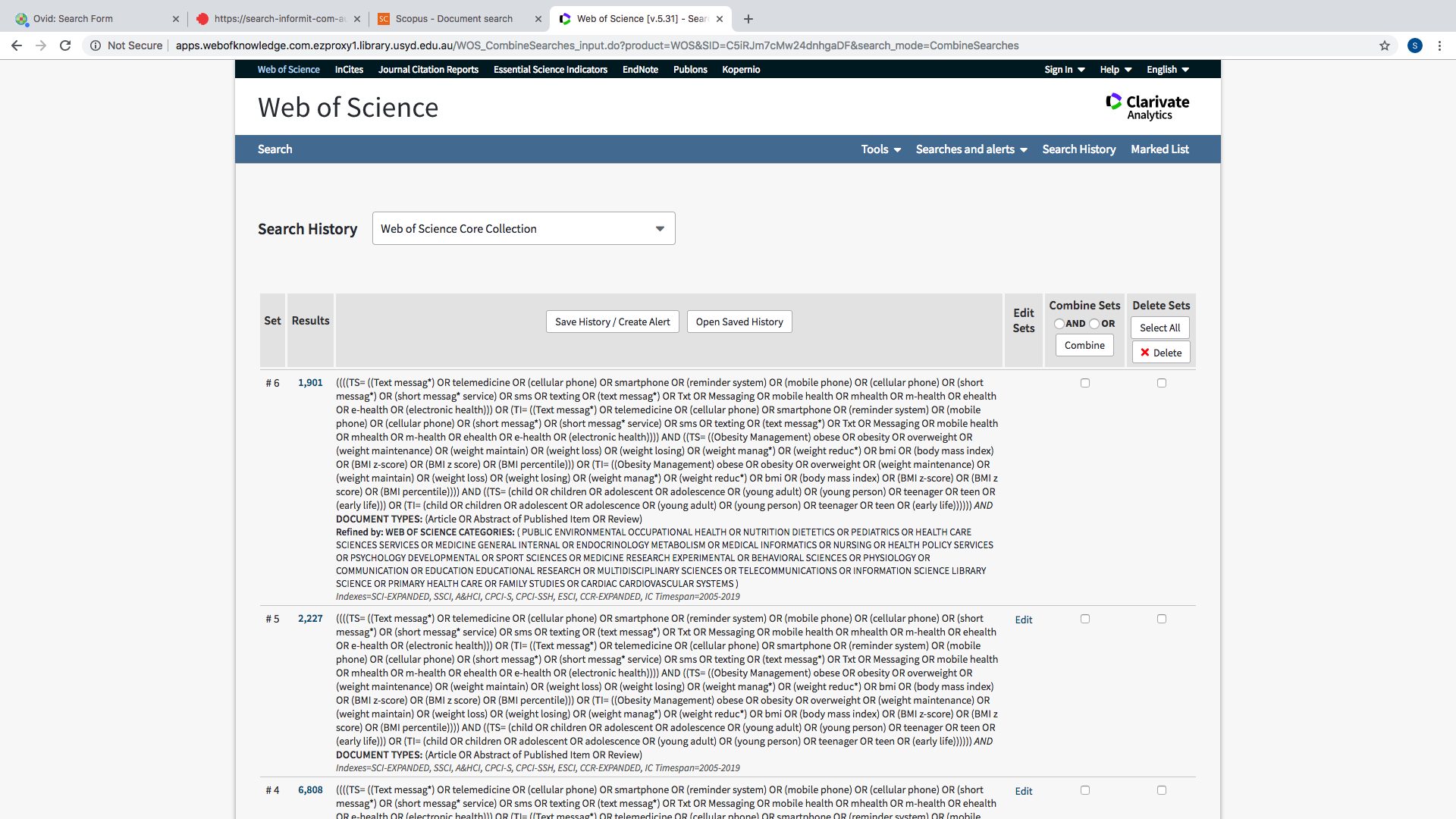


**Figure S10.** Screenshot of full electronic search strategy for Web of Science (21/01/2019)
