# Supplementary material for: Effectiveness of Text Message Interventions for Weight Management in Adolescents: Systematic Review
Source: JMIR Mhealth Uhealth. 2020 May 26;8(5):e15849. doi: 10.2196/15849 (PMC7284408; doi:10.2196/15849)
Supplement: Multimedia Appendix 4 [file mhealth_v8i5e15849_app4.docx]

**Table S11.** Full text excluded and reason for exclusion (n=201)

| **#** | **Reason for exclusion** | **Citation** |
| --- | --- | --- |
| 1 | Abstract only | Buchter D, Kowatsch T, Tanner A, Vural S, Laimbacher J, Mudespacher A, et al. Treatment of adolescents with under-or overweight by stress regulation exercises: An SMS-supported randomized controlled study*. Obesity Facts. 2016;1):211-2. |
| 2 | Abstract only | Heldt K, Buchter DJ, Brogle B, Shih CI, Ruegger D, Filler A, et al. Telemedicine Therapy for Overweight Adolescents: First Results of a Novel Smartphone App Intervention using a Behavioural Health Platform. Obesity Facts. 2018;11 (Supplement 1):214-5. |
| 3 | Abstract only | Silva C, Saint-Maurice P, Goncalves S, Antunes H, Conceicao E, MacHado P. The use of a text-messaging program during weight loss treatment: Baseline results regarding fruits and vegetables intake, screen time and physical activity in obese children and adolescents. Obesity Facts. 2017;10 (Supplement 1):255-6. |
| 4 | Not a text message intervention | Ashton LM, Morgan PJ, Hutchesson MJ, Rollo ME, Collins CE. Feasibility and preliminary efficacy of the 'HEYMAN' healthy lifestyle program for young men: a pilot randomised controlled trial. Nutr J. 2017;16(1):2. |
| 5 | Not a text message intervention | Avis JLS, Cave AL, Donaldson S, Ellendt C, Holt NL, Jelinski S, et al. Working With Parents to Prevent Childhood Obesity: Protocol for a Primary Care-Based eHealth Study. Jmir Research Protocols. 2015;4(1). |
| 6 | Not a text message intervention | Avis JLS, Holt NL, Maximova K, van Mierlo T, Fournier R, Padwal R, et al. The Development and Refinement of an e-Health Screening, Brief Intervention, and Referral to Treatment for Parents to Prevent Childhood Obesity in Primary Care. Telemedicine and E-Health. 2016;22(5):385-94. |
| 7 | Not a text message intervention | Bakhoya M, Ling J, Pfeiffer KA, Robbins LB. Evaluating Mailed Motivational, Individually Tailored Postcard Boosters for Promoting Girls' Postintervention Moderate-to-Vigorous Physical Activity. Nurs Res. 2016;65(5):415-20. |
| 8 | Not a text message intervention | Batch BC, Tyson C, Bagwell J, Corsino L, Intille S, Lin PH, et al. Weight loss intervention for young adults using mobile technology: design and rationale of a randomized controlled trial - Cell Phone Intervention for You (CITY).[Erratum appears in Contemp Clin Trials. 2014 Nov;39(2):351]. Contemp Clin Trials. 2014;37(2):333-41. |
| 9 | Not a text message intervention | Bell BM, Martinez L, Gotsis M, Lane HC, Davis JN, Antunez-Castillo L, et al. Virtual Sprouts: A Virtual Gardening Pilot Intervention Increases Self-Efficacy to Cook and Eat Fruits and Vegetables in Minority Youth. Games for Health Journal. 2018;7(2):127-35. |
| 10 | Not a text message intervention | Bell SL, Audrey S, Cooper AR, Noble S, Campbell R. Lessons from a peer-led obesity prevention programme in English schools. Health Promot Internation. 2017;32(2):250-9. |
| 11 | Not a text message intervention | Bervoets J, Jonkman LM, Mulkens S, de Vries H, Kok G. Enhancing Executive Functions Among Dutch Elementary School Children Using the Train Your Mind Program: Protocol for a Cluster Randomized Trial. Jmir Research Protocols. 2018;7(6). |
| 12 | Not a text message intervention | Bohlin A, Hagman E, Klaesson S, Danielsson P. Childhood obesity treatment: telephone coaching is as good as usual care in maintaining weight loss - a randomized controlled trial. Clin. 2017;7(4):199-205. |
| 13 | Not a text message intervention | Clarke P, Evans S. Fighting Obesity Among Low-Income 9-14 Year Olds: A Home-Based Intervention Using Mobile Phones to Deliver Customized Nutrition Outreach. J Nutr Educ Behav. 2015;47:S104-S. |
| 14 | Not a text message intervention | Clarke P, Evans SH. Fighting Obesity among Low-Income 9-14 Year Olds: A Home-based Intervention Using Mobile Phones to Deliver Customized Nutrition Outreach. J Nutr Educ Behav. 2013;45(4 Supplement):S79-S. |
| 15 | Not a text message intervention | Davis AM, James RL, Boles RE, Goetz JR, Belmont J, Malone B. The use of TeleMedicine in the treatment of paediatric obesity: feasibility and acceptability. Matern Child Nutr. 2011;7(1):71-9. |
| 16 | Not a text message intervention | Davis AM, Sampilo M, Gallagher KS, Dean K, Saroja MB, Yu Q, et al. Treating rural paediatric obesity through telemedicine vs. telephone: Outcomes from a cluster randomized controlled trial. J Telemed Telecare. 2016;22(2):86-95. |
| 17 | Not a text message intervention | Davis AM, Sampilo M, Gallagher KS, Landrum Y, Malone B. Treating rural pediatric obesity through telemedicine: outcomes from a small randomized controlled trial. J Pediatr Psychol. 2013;38(9):932-43. |
| 18 | Not a text message intervention | de Souza RAG, Mediano MFF, Souza AD, Sichieri R. Reducing the use of sugar in public schools: a randomized cluster trial. Rev Saude Publica. 2013;47(4):666-74. |
| 19 | Not a text message intervention | de Visser R, Sylvester R, Rogers R, Kline-Rogers E, DuRussel-Weston J, Eagle KA, et al. Changes in School Health Program Improve Middle School Students' Behaviors. Am J Health Behav. 2016;40(5):568-77. |
| 20 | Not a text message intervention | Flattum C, Friend S, Story M, Neumark-Sztainer D. Evaluation of an Individualized Counseling Approach as Part of a Multicomponent School-Based Program to Prevent Weight-Related Problems among Adolescent Girls. Journal of the American Dietetic Association. 2011;111(8):1218-23. |
| 21 | Not a text message intervention | Fleischman A, Hourigan SE, Lyon HN, Landry MG, Reynolds J, Steltz SK, et al. Creating an integrated care model for childhood obesity: a randomized pilot study utilizing telehealth in a community primary care setting. Clin. 2016;6(6):380-8. |
| 22 | Not a text message intervention | Frenn M, Malin S, Brown RL, Greer Y, Fox J, Greer J, et al. Changing the tide: an Internet/video exercise and low-fat diet intervention with middle-school students. Applied Nursing Research. 2005;18(1):13-21. |
| 23 | Not a text message intervention | Gallagher KS, Davis AM, Malone B, Landrum Y, Black W. Treating rural pediatric obesity through telemedicine: baseline data from a randomized controlled trial. J Pediatr Psychol. 2011;36(6):687-95. |
| 24 | Not a text message intervention | Garde A, Umedaly A, Abulnaga SM, Robertson L, Junker A, Chanoine JP, et al. Assessment of a Mobile Game ("MobileKids Monster Manor") to Promote Physical Activity Among Children. Games for Health Journal. 2015;4(2):149-58. |
| 25 | Not a text message intervention | Gillison F, Standage M, Verplanken B. A cluster randomised controlled trial of an intervention to promote healthy lifestyle habits to school leavers: study rationale, design, and methods. BMC Public Health. 2014;14. |
| 26 | Not a text message intervention | Hingle MD, Turner T, Kutob R, Merchant N, Roe DJ, Stump C, et al. The EPIC Kids Study: a randomized family-focused YMCA-based intervention to prevent type 2 diabetes in at-risk youth. BMC Public Health. 2015;15. |
| 27 | Not a text message intervention | Mackey E, Schweitzer A, Hurtado ME, Hathway J, DiPietro L, Lei KY, et al. The Feasibility of an E-mail-Delivered Intervention to Improve Nutrition and Physical Activity Behaviors in African American College Students. Journal of American College Health. 2015;63(2):109-17. |
| 28 | Not a text message intervention | O'Malley G, Clarke M, Burls A, Murphy S, Murphy N, Perry IJ. A smartphone intervention for adolescent obesity: study protocol for a randomised controlled non-inferiority trial. Trials. 2014;15:43. |
| 29 | Not a text message intervention | Steele RG, Jensen CD, Gayes LA, Leibold HC. Medium Is the Message: Moderate Parental Control of Feeding Correlates With Improved Weight Outcome in a Pediatric Obesity Intervention. J Pediatr Psychol. 2014;39(7):708-17. |
| 30 | Not a text message intervention | Wong JMW, Ebbeling CB, Robinson L, Feldman HA, Ludwig DS. Effects of Advice to Drink 8 Cups of Water per Day in Adolescents With Overweight or Obesity: A Randomized Clinical Trial. Jama, Pediatr. 2017;171(5):e170012. |
| 31 | Not a text message intervention | 전민경, 하주영. BodyThink 프로그램을 적용한 스마트폰 앱의 여자 청소년 비만관리 효과...Effect of Smartphone Apps Applying BodyThink Program on Obesity in Adolescent Girls. Journal of Korean Academy of Nursing. 2016;46(3):390-9. |
| 32 | Protocol, formative or process research | Bailey J, Davies C, McCrossin T, Kiernan M, Skinner R, Steinbeck K, et al. Fit4YAMs: Structuring a Lifestyle Intervention for Rural Overweight and Obese Young Adult Males Using Participatory Design. J Adolesc Health. 2018;62:S65-S71. |
| 33 | Protocol, formative or process research | Barrett N, Colon-Ramos U, Elkins A, Rivera I, Evans WD, Edberg M. Formative Research to Design a Promotional Campaign to Increase Drinking Water among Central American Latino Youth in an Urban Area. J Health Commun. 2017;22(6):459-68. |
| 34 | Protocol, formative or process research | Biggs BK, Lebow J, Smith CM, Harper KL, Patten CA, Sim LA, et al. Adolescents' Preferences for Social Support for Healthful Eating and Physical Activity. Journal of Developmental and Behavioral Pediatrics. 2014;35(8):494-509. |
| 35 | Protocol, formative or process research | Burrows T, Hutchesson M, Li Kheng C, Rollo M, Collins C, Skinner G. Nutrition Interventions for Prevention and Management of Childhood Obesity: What Do Parents Want from an eHealth Program? Nutrients. 2015;7(12):10469-79. |
| 36 | Protocol, formative or process research | Callender C, Thompson D. Text Messaging Based Obesity Prevention Program for Parents of Pre-Adolescent African American Girls. Children-Basel. 2017;4(12). |
| 37 | Protocol, formative or process research | Campbell R, Rawlins E, Wells S, Kipping RR, Chittleborough CR, Peters TJ, et al. Intervention fidelity in a school-based diet and physical activity intervention in the UK: Active for Life Year 5. International Journal of Behavioral Nutrition and Physical Activity. 2015;12. |
| 38 | Protocol, formative or process research | Collins JL, Champion JD. Assessment of Mobile Device and SMS Use for Diet and Exercise Information Among Rural Mexican-American Adolescents. Journal of Pediatric Nursing-Nursing Care of Children & Families. 2014;29(6):493-502. |
| 39 | Protocol, formative or process research | Crane MM, LaRose JG, Espeland MA, Wing RR, Tate DF. Recruitment of young adults for weight gain prevention: randomized comparison of direct mail strategies. Trials. 2016;17(1):282. |
| 40 | Protocol, formative or process research | Epton T, Norman P, Sheeran P, Harris PR, Webb TL, Ciravegna F, et al. A theory-based online health behavior intervention for new university students: study protocol. BMC Public Health. 2013;13. |
| 41 | Protocol, formative or process research | Fjeldsoe B, Phongsavan P, Bauman A, Goode A, Maher G, Eakin E. 'Get Healthy, Stay Healthy': protocol for evaluation of a lifestyle intervention delivered by text-message following the Get Healthy Information and Coaching Service. BMC Public Health. 2014;14:112. |
| 42 | Protocol, formative or process research | Funderburk K, Struempler B, Parmer SM, Griffin JB. Body Quest Parent: A Text Message Parent Education Intervention to Supplement a School-Based Obesity Prevention Initiative for Third-Graders. J Nutr Educ Behav. 2018;50(4):415-7.e1. |
| 43 | Protocol, formative or process research | Gabrielli S, Dianti M, Maimone R, Betta M, Filippi L, Ghezzi M, et al. Design of a Mobile App for Nutrition Education (TreC-LifeStyle) and Formative Evaluation With Families of Overweight Children. Jmir Mhealth and Uhealth. 2017;5(4). |
| 44 | Protocol, formative or process research | Grp HS. HEALTHY study rationale, design and methods: moderating risk of type 2 diabetes in multi-ethnic middle school students. Int J Obes (Lond). 2009;33:S4-S20. |
| 45 | Protocol, formative or process research | Hingle M, Nichter M, Medeiros M, Grace S. Texting for Health: The Use of Participatory Methods to Develop Healthy Lifestyle Messages for Teens. Journal of Nutrition Education and Behavior. 2013;45(1):12-9. |
| 46 | Protocol, formative or process research | Kim KK, Logan HC, Young E, Sabee CM. Youth-centered design and usage results of the iN Touch mobile self-management program for overweight/obesity. Personal and Ubiquitous Computing. 2015;19(1):59-68. |
| 47 | Protocol, formative or process research | Knoblock-Hahn AL, Wray R, LeRouge CM. Perceptions of Adolescents with Overweight and Obesity for the Development of User-Centered Design Self-Management Tools within the Context of the Chronic Care Model: A Qualitative Study. Journal of the Academy of Nutrition and Dietetics. 2016;116(6):957-67. |
| 48 | Protocol, formative or process research | L'Allemand D, Shih CH, Heldt K, Buchter D, Brogle B, Ruegger D, et al. Design and interim evaluation of a smartphone app for overweight adolescents using a behavioural health intervention platform. Obesity Reviews. 2018;19 (Supplement 1):102. |
| 49 | Protocol, formative or process research | Lee JE, Lee DE, Kim K, Shim JE, Sung E, Kang JN, et al. Development of tailored nutrition information messages based on the transtheoretical model for smartphone application of an obesity prevention and management program for elementary-school students. Nutrition Research and Practice. 2017;11(3):247-56. |
| 50 | Protocol, formative or process research | LeRouge C, Dickhut K, Lisetti C, Sangameswaran S, Malasanos T. Engaging adolescents in a computer-based weight management program: avatars and virtual coaches could help. J Am Med Inform Assoc. 2016;23(1):19-28. |
| 51 | Protocol, formative or process research | Livingood WC, Monticalvo D, Bernhardt JM, Wells KT, Harris T, Kee K, et al. Engaging Adolescents Through Participatory and Qualitative Research Methods to Develop a Digital Communication Intervention to Reduce Adolescent Obesity. Health Education & Behavior. 2017;44(4):570-80. |
| 52 | Protocol, formative or process research | Mojica CM, Parra-Medina D, Yin Z, Akopian D, Esparza LA. Assessing media access and use among Latina adolescents to inform development of a physical activity promotion intervention incorporating text messaging. Health Promot Pract. 2014;15(4):548-55. |
| 53 | Protocol, formative or process research | Nguyen B, Shrewsbury V, Lau C, O'Connor J, Steinbeck K, Hill A, et al. Adolescent and parent views of an adolescent weight management program: Lessons from the Loozit randomised controlled trial. Obesity Research and Clinical Practice. 2012;1):56. |
| 54 | Protocol, formative or process research | Nguyen B, Shrewsbury V, O'Connor J, Steinbeck K, Hill A, Shah S, et al. Two-year outcomes of an extended adolescent weight-loss maintenance intervention involving novel additional therapeutic contact: The Loozit randomised controlled trial. Obesity Research and Clinical Practice. 2012;1):38-9. |
| 55 | Protocol, formative or process research | Nguyen B, Shrewsbury V, O'Connor J, Steinbeck K, Hill A, Shah S, et al. Two-year outcomes of an extended adolescent weight-loss maintenance intervention involving novel additional therapeutic contact: The LoozitR randomised controlled trial. Obesity Facts. 2012;1):188-9. |
| 56 | Protocol, formative or process research | Nguyen B, Shrewsbury VA, O'Connor J, Lau C, Steinbeck KS, Hill AJ, et al. A process evaluation of an adolescent weight management intervention: findings and recommendations. Health Promot Internation. 2015;30(2):201-12. |
| 57 | Protocol, formative or process research | O'Malley G, Dowdall G, Burls A, Perry IJ, Curran N. Exploring the Usability of a Mobile App for Adolescent Obesity Management. Jmir Mhealth and Uhealth. 2014;2(2). |
| 58 | Protocol, formative or process research | Park BK, Nahm E-S, Rogers VE, Choi M, Friedmann E, Wilson M, et al. A Facebook-Based Obesity Prevention Program for Korean American Adolescents: Usability Evaluation. Journal of Pediatric Healthcare. 2017;31(1):57-66. |
| 59 | Protocol, formative or process research | Partridge S, Singleton A, Cohen P, Allman-Farinelli M, Mihrshahi S, Chau J, et al. Development of a Bank of Text Messages Targeting Healthy Eating, Physical Activity, and Social Wellbeing In Adolescents At Risk of Obesity. Global Heart. 2018;13 (4):493. |
| 60 | Protocol, formative or process research | Pollard CM, Howat PA, Pratt IS, Boushey CJ, Delp EJ, Kerr DA. Preferred Tone of Nutrition Text Messages for Young Adults: Focus Group Testing. Jmir Mhealth and Uhealth. 2016;4(1):252-65. |
| 61 | Protocol, formative or process research | Poobalan AS, Aucott LS, Clarke A, Smith WCS. Physical activity attitudes, intentions and behaviour among 18-25 year olds: A mixed method study. BMC Public Health. 2012;12. |
| 62 | Protocol, formative or process research | Pope L, Garnett B, Dibble M. Lessons Learned Through the Implementation of an eHealth Physical Activity Gaming Intervention with High School Youth. Games for Health Journal. 2018;7(2):136-42. |
| 63 | Protocol, formative or process research | Price S, Ferisin S, Sharifi M, Steinberg D, Bennett G, Wolin KY, et al. Development and Implementation of an Interactive Text Messaging Campaign to Support Behavior Change in a Childhood Obesity Randomized Controlled Trial. J Health Commun. 2015;20(7):843-50. |
| 64 | Protocol, formative or process research | Reese JM, Joseph RP, Cherrington A, Allison J, Kim YI, Spear B, et al. Development of Participant-Informed Text Messages to Promote Physical Activity Among African American Women Attending College: A Qualitative Mixed-Methods Inquiry. Journal of Transcultural Nursing. 2017;28(3):236-42. |
| 65 | Protocol, formative or process research | Rouf A, Allman-Farinelli M. Messaging for Interventions Aiming to Improve Calcium Intake in Young Adults-A Mixed Methods Study. Nutrients. 2018;10(11). |
| 66 | Protocol, formative or process research | Saez L, Legrand K, Alleyrat C, Ramisasoa S, Langlois J, Muller L, et al. Using facilitator-receiver peer dyads matched according to socioeconomic status to promote behaviour change in overweight adolescents: a feasibility study. BMJ Open. 2018;8(6). |
| 67 | Protocol, formative or process research | Schnall R, Okoniewski A, Tiase V, Low A, Rodriguez M, Kaplan S. Using text messaging to assess adolescents' health information needs: an ecological momentary assessment. J Med Internet Res. 2013;15(3):e54-e. |
| 68 | Protocol, formative or process research | Shrewsbury VA, O'Connor J, Steinbeck KS, Stevenson K, Lee A, Hill AJ, et al. A randomised controlled trial of a community-based healthy lifestyle program for overweight and obese adolescents: the Loozit study protocol. BMC Public Health. 2009;9:119. |
| 69 | Protocol, formative or process research | Smith KL, Kerr DA, Fenner AA, Straker LM. Adolescents just do not know what they want: a qualitative study to describe obese adolescents' experiences of text messaging to support behavior change maintenance post intervention. J Med Internet Res. 2014;16(4):e103-e. |
| 70 | Protocol, formative or process research | Smith KL, Straker LM, Kerr DA, Smith AJ. Overweight adolescents eat what? And when? Analysis of consumption patterns to guide dietary message development for intervention. Journal of Human Nutrition and Dietetics. 2015;28:80-93. |
| 71 | Protocol, formative or process research | Smith KL, Straker LM, McManus A, Fenner AA. Barriers and enablers for participation in healthy lifestyle programs by adolescents who are overweight: a qualitative study of the opinions of adolescents, their parents and community stakeholders. BMC Pediatr. 2014;14. |
| 72 | Protocol, formative or process research | Sousa P, Fonseca H, Gaspar P, Gaspar F. Usability of an internet-based platform (Next.Step) for adolescent weight management. Jornal De Pediatria. 2015;91(1):68-74. |
| 73 | Protocol, formative or process research | Svensson A, Larsson C. A Mobile Phone App for Dietary Intake Assessment in Adolescents: An Evaluation Study. Jmir Mhealth and Uhealth. 2015;3(4):15-35. |
| 74 | Protocol, formative or process research | Tate DF, LaRose JG, Griffin LP, Erickson KE, Robichaud EF, Perdue L, et al. Recruitment of young adults into a randomized controlled trial of weight gain prevention: message development, methods, and cost. Trials. 2014;15:326. |
| 75 | Protocol, formative or process research | Tercyak KP, Abraham AA, Graham AL, Wilson LD, Walker LR, Tercyak KP, et al. Association of multiple behavioral risk factors with adolescents' willingness to engage in eHealth promotion. J Pediatr Psychol. 2009;34(5):457-69. |
| 76 | Protocol, formative or process research | Timpel P, Cesena FHY, da Silva Costa C, Soldatelli MD, Gois E, Castrillon E, et al. Efficacy of gamification-based smartphone application for weight loss in overweight and obese adolescents: study protocol for a phase II randomized controlled trial. Therapeutic Advances in Endocrinology and Metabolism. 2018;9(6):167-76. |
| 77 | Protocol, formative or process research | Ullmann G, Kedia SK, Homayouni R, Akkus C, Schmidt M, Klesges LM, et al. Memphis FitKids: implementing a mobile-friendly web-based application to enhance parents' participation in improving child health. BMC Public Health. 2018;18. |
| 78 | Protocol, formative or process research | Vangeepuram N, Williams N, Constable J, Waldman L, Lopez-Belin P, Phelps-Waldropt L, et al. TEEN HEED: Design of a clinical-community youth diabetes prevention intervention. Contemp Clin Trials. 2017;57:23-8. |
| 79 | Protocol, formative or process research | Wagner KA, Braun E, Armah SM, Horan D, Smith LG, Pike J, et al. Dietary Intervention for Glucose Tolerance In Teens (DIG IT): Protocol of a randomized controlled trial using health coaching to prevent youth-onset type 2 diabetes. Contemp Clin Trials. 2017;53:171-7. |
| 80 | Protocol, formative or process research | Wickham CA, Carbone ET. "Just Say It Like It Is!" Use of a Community-Based Participatory Approach to Develop a Technology-Driven Food Literacy Program for Adolescents. International Quarterly of Community Health Education. 2018;38(2):83-97. |
| 81 | Protocol, formative or process research | Willi SM, Hirst K, Jago R, Buse J, Kaufman F, El Ghormli L, et al. Cardiovascular risk factors in multi-ethnic middle school students: the HEALTHY primary prevention trial. Pediatr Obes. 2012;7(3):230-9. |
| 82 | Protocol, formative or process research | Woolford SJ, Barr KL, Derry HA, Jepson CM, Clark SJ, Strecher VJ, et al. OMG do not say LOL: obese adolescents' perspectives on the content of text messages to enhance weight loss efforts. Obesity (19307381). 2011;19(12):2382-7. |
| 83 | Protocol, formative or process research | Woolford SJ, Clark SJ, Strecher VJ, Resnicow K. Tailored mobile phone text messages as an adjunct to obesity treatment for adolescents. J Telemed Telecare. 2010;16(8):458-61. |
| 84 | Protocol, formative or process research | Woolford SJ, Khan S, Barr KLC, Clark SJ, Strecher VJ, Resnicow K. A picture may be worth a thousand texts: Obese adolescents' perspectives on a modified photovoice activity to aid weight loss. Child. 2012;8(3):230-6. |
| 85 | No control group | Callender C, Thompson D. Family TXT: Feasibility and Acceptability of a mHealth Obesity Prevention Program for Parents of Pre-Adolescent African American Girls. Children-Basel. 2018;5(6). |
| 86 | Not an obesity prevention or management intervention | Ahn SJ. Virtual Exemplars in Health Promotion Campaigns Heightening Perceived Risk and Involvement to Reduce Soft Drink Consumption in Young Adults. Journal of Media Psychology-Theories Methods and Applications. 2018;30(2):91-103. |
| 87 | Not an obesity prevention or management intervention | Bleakley A, Jordan AB, Hennessy M, Glanz K, Strasser A, Vaala S. Do Emotional Appeals in Public Service Advertisements Influence Adolescents' Intention to Reduce Consumption of Sugar-Sweetened Beverages? J Health Commun. 2015;20(8):938-48. |
| 88 | Not an obesity prevention or management intervention | Brown ON, O'Connor LE, Savaiano D. Mobile MyPlate: A Pilot Study Using Text Messaging to Provide Nutrition Education and Promote Better Dietary Choices in College Students. Journal of American College Health. 2014;62(5):320-7. |
| 89 | Not an obesity prevention or management intervention | Coa KI, Augustson E, Kaufman A. The impact of weight and weight-related perceptions on smoking status among young adults in a text-messaging cessation program. Nicotine and Tobacco Research. 2018;20(5):614-9. |
| 90 | Not an obesity prevention or management intervention | De Cock N, Van Lippevelde W, Vangeel J, Notebaert M, Beullens K, Eggermont S, et al. Feasibility and impact study of a reward-based mobile application to improve adolescents' snacking habits. Public Health Nutr. 2018;21(12):2329-44. |
| 91 | Not an obesity prevention or management intervention | Direito A, Jiang YN, Whittaker R, Maddison R. Apps for IMproving FITness and Increasing Physical Activity Among Young People: The AIMFIT Pragmatic Randomized Controlled Trial. J Med Internet Res. 2015;17(8). |
| 92 | Not an obesity prevention or management intervention | Direito A, Jiang YN, Whittaker R, Maddison R. Smartphone apps to improve fitness and increase physical activity among young people: protocol of the Apps for IMproving FITness (AIMFIT) randomized controlled trial. BMC Public Health. 2015;15. |
| 93 | Not an obesity prevention or management intervention | Fassnacht DB, Ali K, Silva C, Gonçalves S, Machado PPP. Use of Text Messaging Services to Promote Health Behaviors in Children. J Nutr Educ Behav. 2015;47(1):75-80. |
| 94 | Not an obesity prevention or management intervention | Filion AJ, Darlington G, Chaput JP, Ybarra M, Haines J. Examining the influence of a text message-based sleep and physical activity intervention among young adult smokers in the United States. BMC Public Health. 2015;15. |
| 95 | Not an obesity prevention or management intervention | Glowacki EM, Kirtz S, Wagner JH, Cance JD, Barrera D, Bernhardt JM. HealthyhornsTXT: A Text-Messaging Program to Promote College Student Health and Wellness. Health Promot Pract. 2018;19(6):844-55. |
| 96 | Not an obesity prevention or management intervention | Grutzmacher SK, Duru EB, Speirs KE, Worthington L, Munger AL, Lachenmayr LA. Using text messages to engage low-income parents in school-based nutrition education. Journal of Hunger & Environmental Nutrition. 2018;13(3):335-9. |
| 97 | Not an obesity prevention or management intervention | Lana A, del Valle MO, Lopez S, Faya-Ornia G, Lopez ML. Study protocol of a randomized controlled trial to improve cancer prevention behaviors in adolescents and adults using a web-based intervention supplemented with SMS. BMC Public Health. 2013;13:357. |
| 98 | Not an obesity prevention or management intervention | Lana A, Faya-Ornia G, Lopez ML. Impact of a web-based intervention supplemented with text messages to improve cancer prevention behaviors among adolescents: results from a randomized controlled trial. Prev Med. 2014;59:54-9. |
| 99 | Not an obesity prevention or management intervention | Leung MM, Green MC, Tate DF, Cai JW, Wyka K, Ammerman AS. Fight for Your Right to Fruit: Psychosocial Outcomes of a Manga Comic Promoting Fruit Consumption in Middle-School Youth. Health Communication. 2017;32(5):533-40. |
| 100 | Not an obesity prevention or management intervention | Liu S, Willoughby JF. Do Fitness Apps Need Text Reminders? An Experiment Testing Goal-Setting Text Message Reminders to Promote Self-Monitoring. J Health Commun. 2018;23(4):379-86. |
| 101 | Not an obesity prevention or management intervention | Lua PL, Wan Dali WPE, Shahril MR. Multimodal Nutrition Education Intervention: A Cluster Randomised Controlled Trial Study on Weight Gain and Physical Activity Pattern Among University Students in Terengganu, Malaysia. Malaysian Journal of Nutrition. 2013;19(3):339-52. |
| 102 | Not an obesity prevention or management intervention | Plummer P, Apple S, Dowd C, Keith E. Texting and walking: Effect of environmental setting and task prioritization on dual-task interference in healthy young adults. Gait & Posture. 2015;41(1):46-51. |
| 103 | Not an obesity prevention or management intervention | Podina IR, Fodor LA, Cosmoiu A, Boian R. An evidence-based gamified mHealth intervention for overweight young adults with maladaptive eating habits: Study protocol for a randomized controlled trial. Trials. 2017;18 (1) (no pagination)(592). |
| 104 | Not an obesity prevention or management intervention | Schweitzer AL, Ross JT, Klein CJ, Lei KY, Mackey ER. An Electronic Wellness Program to Improve Diet and Exercise in College Students: A Pilot Study. Jmir Research Protocols. 2016;5(1). |
| 105 | Not an obesity prevention or management intervention | Silva C, Fassnacht DB, Ali K, Gonçalves S, Conceição E, Vaz A, et al. Promoting health behaviour in Portuguese children via Short Message Service: The efficacy of a text-messaging programme. Journal of Health Psychology. 2015;20(6):806-15. |
| 106 | Not an obesity prevention or management intervention | Trude ACB, Surkan PJ, Cheskin LJ, Gittelsohn J. A multilevel, multicomponent childhood obesity prevention group-randomized controlled trial improves healthier food purchasing and reduces sweet-snack consumption among low-income African-American youth. Nutr J. 2018;17 (1) (no pagination)(96). |
| 107 | Not an obesity prevention or management intervention | Vilchis-Gil J, Klünder-Klünder M, Duque X, Flores-Huerta S. Decreased Body Mass Index in Schoolchildren After Yearlong Information Sessions With Parents Reinforced With Web and Mobile Phone Resources: Community Trial. J Med Internet Res. 2016;18(6):e174-e. |
| 108 | Not an obesity prevention or management intervention | Wang ML, Lemon SC, Clausen K, Whyte J, Rosal MC. Design and methods for a community-based intervention to reduce sugar-sweetened beverage consumption among youth: H(2)GO! study. BMC Public Health. 2016;16. |
| 109 | Includes populations up to 35 years | Allman-Farinelli M, Partridge SR, McGeechan K, Balestracci K, Hebden L, Wong A, et al. A Mobile Health Lifestyle Program for Prevention of Weight Gain in Young Adults (TXT2BFiT): Nine-Month Outcomes of a Randomized Controlled Trial. Jmir Mhealth and Uhealth. 2016;4(2):408-19. |
| 110 | Includes populations up to 35 years | Godino JG, Merchant G, Norman GJ, Donohue MC, Marshall SJ, Fowler JH, et al. Using social and mobile tools for weight loss in overweight and obese young adults (Project SMART): a 2 year, parallel-group, randomised, controlled trial. Lancet Diabetes Endocrinol. 2016;4(9):747-55. |
| 111 | Includes populations up to 35 years | Hebden L, Balestracci K, McGeechan K, Denney-Wilson E, Harris M, Bauman A, et al. 'TXT2BFiT' a mobile phone-based healthy lifestyle program for preventing unhealthy weight gain in young adults: study protocol for a randomized controlled trial. Trials. 2013;14:75. |
| 112 | Includes populations up to 35 years | Hebden L, Cook A, van der Ploeg HP, King L, Bauman A, Allman-Farinelli M. A mobile health intervention for weight management among young adults: a pilot randomised controlled trial. J Hum Nutr Diet. 2014;27(4):322-32. |
| 113 | Includes populations up to 35 years | Hutchesson MJ, Callister R, Morgan PJ, Pranata I, Clarke ED, Skinner G, et al. A Targeted and Tailored eHealth Weight Loss Program for Young Women: The Be Positive Be Healthe Randomized Controlled Trial. Healthcare. 2018;6(2). |
| 114 | Includes populations up to 35 years | Hutchesson MJ, Morgan PJ, Callister R, Pranata I, Skinner G, Collins CE. Be Positive Be Healthe: Development and Implementation of a Targeted e-Health Weight Loss Program for Young Women. Telemedicine and E-Health. 2016;22(6):519-28. |
| 115 | Includes populations up to 35 years | Jakicic JM, Davis KK, Rogers RJ, King WC, Marcus MD, Helsel D, et al. Effect of Wearable Technology Combined With a Lifestyle Intervention on Long-term Weight Loss: The IDEA Randomized Clinical Trial.[Erratum appears in JAMA. 2016 Oct 11;316(14 ):1498; PMID: 27658229]. Jama. 2016;316(11):1161-71. |
| 116 | Includes populations up to 35 years | Kerr DA, Harray AJ, Pollard CM, Dhaliwal SS, Delp EJ, Howat PA, et al. The connecting health and technology study: a 6-month randomized controlled trial to improve nutrition behaviours using a mobile food record and text messaging support in young adults. Int. 2016;13:52. |
| 117 | Includes populations up to 35 years | Lin PH, Grambow S, Intille S, Gallis JA, Lazenka T, Bosworth H, et al. The Association Between Engagement and Weight Loss Through Personal Coaching and Cell Phone Interventions in Young Adults: Randomized Controlled Trial. Jmir Mhealth and Uhealth. 2018;6(10). |
| 118 | Includes populations up to 35 years | Lin PH, Intille S, Bennett G, Bosworth HB, Corsino L, Voils C, et al. Adaptive intervention design in mobile health: Intervention design and development in the Cell Phone Intervention for You trial. Clin. 2015;12(6):634-45. |
| 119 | Includes populations up to 35 years | Merchant G, Weibel N, Patrick K, Fowler JH, Norman GJ, Gupta A, et al. Click "like" to change your behavior: a mixed methods study of college students' exposure to and engagement with Facebook content designed for weight loss. J Med Internet Res. 2014;16(6):e158. |
| 120 | Includes populations up to 35 years | Merchant G, Weibel N, Pina L, Griswold WG, Fowler JH, Ayala GX, et al. Face-to-Face and Online Networks: College Students' Experiences in a Weight-Loss Trial. J Health Commun. 2017;22(1):75-83. |
| 121 | Includes populations up to 35 years | Napolitano MA, Hayes S, Bennett GG, Ives AK, Foster GD. Using Facebook and text messaging to deliver a weight loss program to college students. Obesity (Silver Spring). 2013;21(1):25-31. |
| 122 | Includes populations up to 35 years | Napolitano MA, Whiteley JA, Mavredes MN, Faro J, DiPietro L, Hayman LL, et al. Using social media to deliver weight loss programming to young adults: Design and rationale for the Healthy Body Healthy U (HBHU) trial. Contemp Clin Trials. 2017;60:1-13. |
| 123 | Includes populations up to 35 years | Partridge SR, McGeechan K, Hebden L, Balestracci K, Wong ATY, Denney-Wilson E, et al. Effectiveness of a mHealth Lifestyle Program With Telephone Support (TXT2BFiT) to Prevent Unhealthy Weight Gain in Young Adults: Randomized Controlled Trial. Jmir Mhealth and Uhealth. 2015;3(2). |
| 124 | Includes populations up to 35 years | Patrick K, Marshall SJ, Davila EP, Kolodziejczyk JK, Fowler JH, Calfas KJ, et al. Design and implementation of a randomized controlled social and mobile weight loss trial for young adults (project SMART). Contemp Clin Trials. 2014;37(1):10-8. |
| 125 | Includes populations up to 35 years | Svetkey LP, Batch BC, Lin PH, Intille SS, Corsino L, Tyson CC, et al. Cell phone intervention for you (CITY): A randomized, controlled trial of behavioral weight loss intervention for young adults using mobile technology. Obesity (Silver Spring). 2015;23(11):2133-41. |
| 126 | Not a healthy adolescent population | Abel ML, Lee K, Loglisci R, Righter A, Hipper TJ, Cheskin LJ. Consumer understanding of calorie labeling: a healthy monday e-mail and text message intervention. Health Promot Pract. 2015;16(2):236-43. |
| 127 | Not a healthy adolescent population | Ahn A, Choi J. A one-way text messaging intervention for obesity. Journal of telemedicine and telecare. 2016;22(3):148-52. |
| 128 | Not a healthy adolescent population | Ahrendt AD, Kattelmann KK, Rector TS, Maddox DA. The effectiveness of telemedicine for weight management in the move! program. Journal of Rural Health. 2014;30(1):113-9. |
| 129 | Not a healthy adolescent population | Armstrong S, Mendelsohn A, Bennett G, Taveras EM, Kimberg A, Kemper AR. Texting Motivational Interviewing: A Randomized Controlled Trial of Motivational Interviewing Text Messages Designed to Augment Childhood Obesity Treatment. Childhood obesity (Print). 2018;14(1):4-10. |
| 130 | Not a healthy adolescent population | Babic MJ, Morgan PJ, Plotnikoff RC, Lonsdale C, Eather N, Skinner G, et al. Rationale and study protocol for 'Switch-off 4 Healthy Minds' (S4HM): a cluster randomized controlled trial to reduce recreational screen time in adolescents. Contemp Clin Trials. 2015;40:150-8. |
| 131 | Not a healthy adolescent population | Babic MJ, Smith JJ, Morgan PJ, Lonsdale C, Plotnikoff RC, Eather N, et al. Intervention to reduce recreational screen-time in adolescents: Outcomes and mediators from the 'Switch-Off 4 Healthy Minds' (S4HM) cluster randomized controlled trial. Prev Med. 2016;91:50-7. |
| 132 | Not a healthy adolescent population | Bannon K, Schwartz MB. Impact of nutrition messages on children's food choice: Pilot study. Appetite. 2006;46(2):124-9. |
| 133 | Not a healthy adolescent population | Bauer S, de Niet J, Timman R, Kordy H, Bauer S, de Niet J, et al. Enhancement of care through self-monitoring and tailored feedback via text messaging and their use in the treatment of childhood overweight. Patient Education & Counseling. 2010;79(3):315-9. |
| 134 | Not a healthy adolescent population | Berli C, Stadler G, Inauen J, Scholz U. Action control in dyads: A randomized controlled trial to promote physical activity in everyday life. Social Science and Medicine. 2016;163:89-97. |
| 135 | Not a healthy adolescent population | Bouhaidar CM, Deshazo JP, Puri P, Gray P, Robins JLW, Salyer J. Text Messaging as Adjunct to Community-Based Weight Management Program. CIN: Computers, Informatics, Nursing. 2013;31(10):469-76. |
| 136 | Not a healthy adolescent population | Broekhuizen K, van Poppel MN, Koppes LL, Brug J, van Mechelen W. A tailored lifestyle intervention to reduce the cardiovascular disease risk of individuals with Familial Hypercholesterolemia (FH): design of the PRO-FIT randomised controlled trial. BMC Public Health. 2010;10:69. |
| 137 | Not a healthy adolescent population | Clark DO, Srinivas P, Bodke K, Keith N, Hood S, Tu W. Addressing people and place microenvironments in weight loss disparities (APP-Me): Design of a randomized controlled trial testing timely messages for weight loss behavior in low income Black and White Women. Contemp Clin Trials. 2018;67:74-80. |
| 138 | Not a healthy adolescent population | Collins CE, Dewar DL, Schumacher TL, Finn T, Morgan PJ, Lubans DR. 12 Month changes in dietary intake of adolescent girls attending schools in low-income communities following the NEAT Girls cluster randomized controlled trial. Appetite. 2014;73:147-55. |
| 139 | Not a healthy adolescent population | de Niet J, Timman R, Bauer S, van den Akker E, Buijks H, de Klerk C, et al. The effect of a short message service maintenance treatment on body mass index and psychological well-being in overweight and obese children: a randomized controlled trial. Pediatr Obes. 2012;7(3):205-19. |
| 140 | Not a healthy adolescent population | de Niet J, Timman R, Bauer S, van den Akker E, de Klerk C, Kordy H, et al. Short message service reduces dropout in childhood obesity treatment: a randomized controlled trial. Health Psychol. 2012;31(6):797-805. |
| 141 | Not a healthy adolescent population | Dewar DL, Morgan PJ, Plotnikoff RC, Okely AD, Batterham M, Lubans DR. Exploring changes in physical activity, sedentary behaviors and hypothesized mediators in the NEAT girls group randomized controlled trial. J Sci Med Sport. 2014;17(1):39-46. |
| 142 | Not a healthy adolescent population | Ermetici F, Zelaschi RF, Briganti S, Dozio E, Gaeta M, Ambrogi F, et al. Association between a school-based intervention and adiposity outcomes in adolescents: The Italian "EAT" project. Obesity (19307381). 2016;24(3):687-95. |
| 143 | Not a healthy adolescent population | Evans WD, Christoffel KK, Necheles J, Becker AB, Snider J. Outcomes of the 5-4-3-2-1 Go! Childhood obesity community trial. Am J Health Behav. 2011;35(2):189-98. |
| 144 | Not a healthy adolescent population | Evans WD, Wallace J, Snider J. The 5-4-3-2-1 Go! Brand to Promote Nutrition and Physical Activity: A Case of Positive Behavior Change but Negative Change in Beliefs. J Health Commun. 2015;20(5):512-20. |
| 145 | Not a healthy adolescent population | Hennink-Kaminski H, Vaughn AE, Hales D, Moore RH, Luecking CT, Ward DS. Parent and child care provider partnerships: Protocol for the Healthy Me, Healthy We (HMHW) cluster randomized control trial. Contemp Clin Trials. 2018;64:49-57. |
| 146 | Not a healthy adolescent population | Hopkins LC, Webster A, Sharn A, Gunther C. Camp NERF: Caregiver outcomes from a theory-based nutrition education recreation and fitness program aimed at preventing unhealthy weight gain in underserved children during summer months. FASEB Journal Conference: Experimental Biology. 2017;31(1 Supplement 1). |
| 147 | Not a healthy adolescent population | Lubans D, Dewar D, Morgan P, Plotnikoff R, Okely A, Collins C, et al. Two-year outcomes from the NEAT Girls obesity prevention cluster randomized controlled trial. Journal of Science and Medicine in Sport. 2013;1):e34. |
| 148 | Not a healthy adolescent population | Lubans D, Morgan P, Okely A, Dewar D, Collins C, Batterham M, et al. Preventing obesity among adolescent girls in lowincome secondary schools: One-year outcomes of the NEAT Girls cluster randomized controlled trial. Obesity Research and Clinical Practice. 2012;1):39. |
| 149 | Not a healthy adolescent population | Lubans D, Morgan P, Okely A, Dewar D, Collins C, Batterham M, et al. Preventing obesity among adolescent girls: Outcomes of the nutrition and enjoyable activity for teen girls cluster randomized controlled trial. Journal of Science and Medicine in Sport. 2012;15 (SUPPL.1):S332. |
| 150 | Not a healthy adolescent population | Lubans DR, Morgan PJ, Dewar D, Collins CE, Plotnikoff RC, Okely AD, et al. The Nutrition and Enjoyable Activity for Teen Girls (NEAT girls) randomized controlled trial for adolescent girls from disadvantaged secondary schools: rationale, study protocol, and baseline results. BMC Public Health. 2010;10:652. |
| 151 | Not a healthy adolescent population | Lubans DR, Morgan PJ, Okely AD, Dewar D, Collins CE, Batterham M, et al. Preventing obesity among adolescent girls: One-year outcomes of the nutrition and enjoyable activity for teen girls (NEAT Girls) cluster randomized controlled trial. Archives of Pediatrics and Adolescent Medicine. 2012;166(9):821-7. |
| 152 | Not a healthy adolescent population | Lubans DR, Smith JJ, Peralta LR, Plotnikoff RC, Okely AD, Salmon J, et al. A school-based intervention incorporating smartphone technology to improve health-related fitness among adolescents: rationale and study protocol for the NEAT and ATLAS 2.0 cluster randomised controlled trial and dissemination study. BMJ Open. 2016;6(6):e010448. |
| 153 | Not a healthy adolescent population | Lubans DR, Smith JJ, Plotnikoff RC, Dally KA, Okely AD, Salmon J, et al. Assessing the sustained impact of a school-based obesity prevention program for adolescent boys: The ATLAS cluster randomized controlled trial. International Journal of Behavioral Nutrition and Physical Activity. 2016;13 (1) (no pagination)(92). |
| 154 | Not a healthy adolescent population | Marcus MD, Hirst K, Kaufman F, Foster GD, Baranowski T. Lessons learned from the HEALTHY primary prevention trial of risk factors for type 2 diabetes in middle school youth. Curr Diab Rep. 2013;13(1):63-71. |
| 155 | Not a healthy adolescent population | Nollen NL, Mayo MS, Carlson SE, Rapoff MA, Goggin KJ, Ellerbeck EF. Mobile Technology for Obesity Prevention A Randomized Pilot Study in Racial- and Ethnic-Minority Girls. American Journal of Preventive Medicine. 2014;46(4):404-8. |
| 156 | Not a healthy adolescent population | Novotny R, Davis J, Butel J, Boushey CJ, Fialkowski MK, Nigg CR, et al. Effect of the Children's Healthy Living Program on Young Child Overweight, Obesity, and Acanthosis Nigricans in the US-Affiliated Pacific Region A Randomized Clinical Trial. Jama Network Open. 2018;1(6). |
| 157 | Not a healthy adolescent population | O'Brien LM, Palfai TP. Efficacy of a brief web-based intervention with and without SMS to enhance healthy eating behaviors among university students. Eating Behaviors. 2016;23:104-9. |
| 158 | Not a healthy adolescent population | Pellegrini CA, Duncan JM, Moller AC, Buscemi J, Sularz A, DeMott A, et al. A smartphone-supported weight loss program: design of the ENGAGED randomized controlled trial. BMC Public Health. 1041;12. |
| 159 | Not a healthy adolescent population | Pittman AF. EFFECT OF ACTIVITY TRACKERS AND TEXT MESSAGING ON EXERCISE, FITNESS, AND PHYSICAL ACTIVITY SELF-EFFICACY OF MIDDLE SCHOOL STUDENTS. Effect of Activity Trackers & Text Messaging on Exercise, Fitness & Physical Activity Self-Efficacy of Middle School Students. 2016:1-. |
| 160 | Not a healthy adolescent population | Rangelov N, Della Bella S, Marques-Vidal P, Suggs LS. Does additional support provided through e-mail or SMS in a Web-based Social Marketing program improve children's food consumption? A Randomized Controlled Trial. Nutr J. 2018;17. |
| 161 | Not a healthy adolescent population | Robbins LB, Pfeiffer KA, Vermeesch A, Resnicow K, You ZY, An L, et al. "Girls on the Move" intervention protocol for increasing physical activity among low-active underserved urban girls: a group randomized trial. BMC Public Health. 2013;13. |
| 162 | Not a healthy adolescent population | Shapiro JR, Bauer S, Hamer RM, Kordy H, Ward D, Bulik CM. Use of text messaging for monitoring sugar-sweetened beverages, physical activity, and screen time in children: a pilot study. J Nutr Educ Behav. 2008;40(6):385-91. |
| 163 | Not a healthy adolescent population | Smith JJ, Morgan PJ, Plotnikoff RC, Dally KA, Salmon J, Okely AD, et al. Rationale and study protocol for the 'Active Teen Leaders Avoiding Screen-time' (ATLAS) group randomized controlled trial: An obesity prevention intervention for adolescent boys from schools in low-income communities. Contemp Clin Trials. 2014;37(1):106-19. |
| 164 | Not a healthy adolescent population | Smith JJ, Morgan PJ, Plotnikoff RC, Dally KA, Salmon J, Okely AD, et al. Smart-phone obesity prevention trial for adolescent boys in low-income communities: the ATLAS RCT. Pediatrics. 2014;134(3):e723-31. |
| 165 | Not a healthy adolescent population | Stephens JD, Yager AM, Allen J. Smartphone Technology and Text Messaging for Weight Loss in Young Adults: A Randomized Controlled Trial. J Cardiovasc Nurs. 2017;32(1):39-46. |
| 166 | Not a healthy adolescent population | Stephens JD, Yager AM, Allen J. Smartphone Technology and Text Messaging for Weight Loss in Young Adults. J Cardiovasc Nurs. 2017;32(1):39-46. |
| 167 | Not a healthy adolescent population | Vilchis-Gil J, Klunder-Klunder M, Flores-Huerta S. Effect on the Metabolic Biomarkers in Schoolchildren After a Comprehensive Intervention Using Electronic Media and In-Person Sessions to Change Lifestyles: Community Trial. J Med Internet Res. 2018;20(2). |
| 168 | Not a healthy adolescent population | Wilson CJ, de la Haye K, Coveney J, Hughes DL, Hutchinson A, Miller C, et al. Protocol for a randomized controlled trial testing the impact of feedback on familial risk of chronic diseases on family-level intentions to participate in preventive lifestyle behaviors. BMC Public Health. 2016;16. |
| 169 | Not a healthy adolescent population | Yang HJ, Kang JH, Kim OH, Choi M, Oh M, Nam J, et al. Interventions for preventing childhood obesity with smartphones and wearable device: A protocol for a non-randomized controlled trial. International Journal of Environmental Research and Public Health. 2017;14 (2) (no pagination)(184). |
| 170 | Not an RCT | Adachi-Mejia AM, Edwards PM, Gilbert-Diamond D, Greenough GP, Olson AL. TXT Me I'm Only Sleeping: Adolescents With Mobile Phones in Their Bedroom. Family & Community Health. 2014;37(4):252-7. |
| 171 | Not an RCT | Adhikari PD, Parker LA, Binns HJ, Ariza AJ. Influence of Electronic Health Records and In-office Weight Management Support Resources on Childhood Obesity Care. Clin Pediatr (Phila). 2012;51(8):788-92. |
| 172 | Not an RCT | Arora T, Hussain S, Lam KBH, Yao GL, Thomas GN, Taheri S. Exploring the complex pathways among specific types of technology, self-reported sleep duration and body mass index in UK adolescents. Int J Obes (Lond). 2013;37(9):1254-60. |
| 173 | Not an RCT | Banos RM, Cebolla A, Zaragoza I, Botella C, Alcaniz M. Electronic PDA dietary and physical activity registers in a weight loss treatment program for children: A description of the etiobe personal digital assistant system. Journal of Cyber Therapy and Rehabilitation. 2009;2(3):235-41. |
| 174 | Not an RCT | Baranowski T, Frankel L. Let's get technical! gaming and technology for weight control and health promotion in children. Child. 2012;8(1):34-7. |
| 175 | Not an RCT | Baranowski T, Maddison R, Maloney A, Medina E, Simons M. Building a Better Mousetrap (Exergame) to Increase Youth Physical Activity. Games for Health Journal. 2014;3(2):72-8. |
| 176 | Not an RCT | Besenyi GM, Diehl P, Schooley B, Turner-McGrievy BM, Wilcox S, Stanis SAW, et al. Development and testing of mobile technology for community park improvements: validity and reliability of the eCPAT application with youth. Transl Behav Med. 2016;6(4):519-32. |
| 177 | Not an RCT | Besenyi GM, Schooley B, Turner-McGrievy GM, Wilcox S, Stanis SAW, Kaczynski AT. The Electronic Community Park Audit Tool (eCPAT): Exploring the Use of Mobile Technology for Youth Empowerment and Advocacy for Healthy Community Policy, Systems, and Environmental Change. Frontiers in Public Health. 2018;6. |
| 178 | Not an RCT | Blanson Henkemans OA, van der Boog PJ, Lindenberg J, van der Mast CA, Neerincx MA, Zwetsloot-Schonk BJ. An online lifestyle diary with a persuasive computer assistant providing feedback on self-management. Technology and health care. 2009;17(3):253-67. |
| 179 | Not an RCT | Bodner ME, Lyna P, Ostbye T, Bravender T, Alexander SC, Tulsky JA, et al. Accuracy and congruence of physician and adolescent patient weight-related discussions: Teen CHAT (Communicating health: Analyzing talk). Patient Education and Counseling. 2018;101(12):2105-10. |
| 180 | Not an RCT | Borgogna N, Lockhart G, Grenard JL, Barrett T, Shiffman S, Reynolds KD. Ecological Momentary Assessment of Urban Adolescents’ Technology Use and Cravings for Unhealthy Snacks and Drinks: Differences by Ethnicity and Sex. J Acad Nutr Diet. 2015;115(5):759-66. |
| 181 | Not an RCT | Bruselius-Jensen M, Hansen AKV, Danielsen D. Pedometers and participatory school-based health education - an exploratory study. Health Education (0965-4283). 2014;114(6):1-24. |
| 182 | Not an RCT | Buchter D, Kowatsch T, Brogle B, Dintheer-Ter-Velde A, Wiegand D, Pletikosa I, et al. Home support of obese children and adolescents by means of health information technology system: A pilot study for a psychosomatic therapy concept. Swiss Medical Weekly. 2014;203):17S. |
| 183 | Not an RCT | Cerin E, Barnett A, Baranowski T. Testing Theories of Dietary Behavior Change in Youth Using the Mediating Variable Model with Intervention Programs. Journal of Nutrition Education and Behavior. 2009;41(5):309-18. |
| 184 | Not an RCT | Coles N, Patel BP, Li P, Cordeiro K, Steinberg A, Zdravkovic A, et al. Breaking barriers: Adjunctive use of the Ontario Telemedicine Network (OTN) to reach adolescents with obesity living in remote locations. Journal of Telemedicine and Telecare. 2018. |
| 185 | Not an RCT | De Cock N, Vangeel J, Lachat C, Beullens K, Vervoort L, Goossens L, et al. Use of Fitness and Nutrition Apps: Associations With Body Mass Index, Snacking, and Drinking Habits in Adolescents. Jmir Mhealth and Uhealth. 2017;5(4). |
| 186 | Not an RCT | Diez-Canseco F, Boeren Y, Quispe R, Chiang ML, Miranda JJ. Engagement of Adolescents in a Health Communications Program to Prevent Noncommunicable Diseases: Multiplicadores Jovenes, Lima, Peru, 2011. Preventing Chronic Disease. 2015;12. |
| 187 | Not an RCT | Fernandez-Luque L, Singh M, Ofli F, Mejova YA, Weber I, Aupetit M, et al. Implementing 360degree Quantified Self for childhood obesity: feasibility study and experiences from a weight loss camp in Qatar.[Erratum appears in BMC Med Inform Decis Mak. 2017 May 12;17 (1):62; PMID: 28499380]. BMC Med Inf Decis Mak. 2017;17(1):37. |
| 188 | Not an RCT | Jensen CD, Aylward BS, Steele RG. Predictors of attendance in a practical clinical trial of two pediatric weight management interventions. Obesity (Silver Spring). 2012;20(11):2250-6. |
| 189 | Not an RCT | Jensen CD, Duncombe KM, Lott MA, Hunsaker SL, Duraccio KM, Woolford SJ. An Evaluation of a Smartphone-Assisted Behavioral Weight Control Intervention for Adolescents: Pilot Study. Jmir Mhealth and Uhealth. 2016;4(3). |
| 190 | Not an RCT | Kay MC, Burroughs J, Askew S, Bennett GG, Armstrong S, Steinberg DM. Digital Weight Loss Intervention for Parents of Children Being Treated for Obesity: A Prospective Cohort Feasibility Trial. J Med Internet Res. 2018;20(12). |
| 191 | Not an RCT | Kramer EN, Chard CA, Walters K, Barr-Anderson DJ. Weight-Dependent Disparities in Adolescent Girls: The Impact of a Brief Pilot Intervention on Exercise and Healthy Eater Identity. International Journal of Environmental Research and Public Health. 2018;15(7). |
| 192 | Not an RCT | Lindqvist AK, Mikaelsson K, Westerberg M, Gard G, Kostenius C. Moving from idea to action: promoting physical activity by empowering adolescents. Health Promot Pract. 2014;15(6):812-8. |
| 193 | Not an RCT | Markert J, Herget S, Marschke S, Lehnert T, Falkenberg C, Bluher S. Case management via telephone counseling and SMS for weight maintenance in adolescent obesity: Study concept of the TeAM program. BMC Obesity. 2014;1 (1) (no pagination)(8). |
| 194 | Not an RCT | Markert J, Herget S, Marschke S, Lehnert T, Falkenberg C, Bluher S. Case management via telephone counseling and SMS for weight maintenance in adolescent obesity: Study concept of the TeAM program. BMC Obesity. 2014;1 (1) (no pagination)(8). |
| 195 | Not an RCT | Saez L, Langlois J, Legrand K, Quinet MH, Lecomte E, Omorou AY, et al. Reach and Acceptability of a Mobile Reminder Strategy and Facebook Group Intervention for Weight Management in Less Advantaged Adolescents: Insights From the PRALIMAP-INES Trial. Jmir Mhealth and Uhealth. 2018;6(5). |
| 196 | Not an RCT | Schiel R, Kaps A, Bieber G, Schiel R, Kaps A, Bieber G. Electronic health technology for the assessment of physical activity and eating habits in children and adolescents with overweight and obesity IDA. Appetite. 2012;58(2):432-7. |
| 197 | Not an RCT | Schultz AT, Markowitz JT, Cousineau TM, Franko DL, Laffel LM. Mobile health (mHealth) intervention called BodiMojo using text messaging aimed at healthy lifestyles for youth with diabetes (DM): A pilot Randomized Controlled Trial (RCT). Diabetes. 2013;1):A342. |
| 198 | Not an RCT | Smith KL, Kerr DA, Howie EK, Straker LM. Do Overweight Adolescents Adhere to Dietary Intervention Messages? Twelve-Month Detailed Dietary Outcomes from Curtin University's Activity, Food and Attitudes Program. Nutrients. 2015;7(6):4363-82. |
| 199 | Not an RCT | Straker LM, Howie EK, Smith KL, Fenner AA, Kerr DA, Olds TS, et al. The impact of Curtin University's activity, food and attitudes program on physical activity, sedentary time and fruit, vegetable and junk food consumption among overweight and obese adolescents: a waitlist controlled trial. PLoS ONE. 2014;9(11):e111954. |
| 200 | Not an RCT | Thompson D, Baranowski T, Cullen K, Watson K, Liu Y, Canada A, et al. Food, fun, and fitness internet program for girls: pilot evaluation of an e-Health youth obesity prevention program examining predictors of obesity. Prev Med. 2008;47(5):494-7. |
| 201 | Not an RCT | Tu AW, Watts AW, Chanoine JP, Panagiotopoulos C, Geller J, Brant R, et al. Does parental and adolescent participation in an e-health lifestyle modification intervention improve weight outcomes? BMC Public Health. 2017;17. |
