# Supplementary material for: Effectiveness of Text Message Interventions for Weight Management in Adolescents: Systematic Review
Source: JMIR Mhealth Uhealth. 2020 May 26;8(5):e15849. doi: 10.2196/15849 (PMC7284408; doi:10.2196/15849)
Supplement: Multimedia Appendix 5 [file mhealth_v8i5e15849_app5.docx]

**Table S12.** Behaviour change techniques, text message development and process measures in individual included studies (n=8)

| **Author, Year, Citation** | **Behaviour change techniques in text messages** | **Text message development** | **Adherence and process measures** |
| --- | --- | --- | --- |
| Abraham et al. 2015 | 1. Goals and planning  1.1 Goal setting (behaviour)  1.3 Goal setting (outcome)  1.7 Review outcome goal(s)  8. Repetition and substitution  8.3 Habit formation  9. Comparison of outcomes  9.1 Credible source | Participants: 11 American Chinese adolescents (10 M, 1 F)  Methods: Focus groups that asked open-ended questions about the use of text messages  Results: Agreed weekly individualised text message reminders could be a good way to enhance their motivation to adopt healthy behaviours | - 400 text messages sent to 16 participants - 15 agreed to receive via WhatsApp and 1 via email - 78.3% responded to diet goals and 77.5% to exercise goals - Research assistant spent 2 hours per week sending personalised text messages |
| Bagherniya et al. 2018 | 1. Goals and planning  1.1 Goal setting (behaviour)  4. Shaping knowledge  4.1 Instructions on how to perform the behaviour  8. Repetition and substitution  8.3 Habit formation  9. Comparison of outcomes  9.1 Credible source | NR | NR |
| Chen et al. 2017 | 1. Goals and planning  1.1 Goal setting (behaviour)  1.3 Goal setting (outcome)  1.4 Action planning  4. Shaping knowledge  4.1 Instructions on how to perform the behaviour  7. Associations  7.1 Prompts/cues  8. Repetition and substitution  8.3 Habit formation  9. Comparison of outcomes  9.1 Credible source | Participants: Advisory group consisting of 2 primary care physicians and 4 adolescents and further consultation with 10 adolescents  Methods: Advisory group that identified purpose, goals and 8-modules of the program and focus groups piloted the FitBit procedures and appropriateness of the intervention content  Results: Adolescents ‘liked the program’ and minor changes were made.  Note: No specific details relating to text message development were provided. | - 17/ 23 intervention participants reported accessing the Fitbit program via the app or website several times a week - 5/23 adolescents accessed the program once a week - All who used the Fitbit Flex reported that the device was helpful in tracking physical activity level - 88% found the device helpful in tracking physical activity food intake - All would recommend this program to others - 91% shared their Fitbit data with their primary care providers |
| Jensen et al. 2019, USA | 1. Goals and planning  1.1 Goal setting (behaviour)  1.3 Goal setting (outcome)  2. Feedback and monitoring  2.3 Self-monitoring of behaviour  4. Shaping knowledge  4.1 Instructions on how to perform the behaviour  7. Associations  7.1 Prompts/cues  8. Repetition and substitution  8.3 Habit formation  9. Comparison of outcomes  9.1 Credible source | Participants: 20 adolescents mean age 14 years; 85% female and 77% Caucasian  Methods: Text messages were developed by research team and tested in 3-month feasibility and acceptability trial, where 1 text message was sent each day. Participants completed surveys at monthly intervals and participated in semi-structured interview at the end of study.  Results: Liked receiving text messages after school. Overall, they liked the text messages and could recall topics they addressed. A greater variety of messages were suggested, the most popular messages were recipe ideas, followed by testimonials and messages with pictures. Most found the messages personally relevant and they helped them to make healthy choices and kept them focused on weight management | - Over 6-months intervention participants sent self-monitoring text messages on 47% of intervention days, compared to 22% control participants - Intervention participants demonstrated significantly greater self-monitoring adherence (P < 0.01) - Text message adherence in first week was 67% for all participants, 51% at 3-months and 63% during last week of the intervention - 85% of intervention participants found the adaptive text messages helpful - 46% found the text messages made them accountable - 69% found the text message content annoying and repetitive - 54% would prefer fewer text messages - 54% would prefer more personalised text messages |
| Love-Osborne et al. 2016 | 1. Goals and planning  1.1 Goal setting (behaviour)  7. Associations  7.1 Prompts/cues  9. Comparison of outcomes  9.1 Credible source | NR | - 8 intervention participants received a mobile phone provided by research team - Text messages sent via Microsoft Outlook which was successful for 30/38 participants - 8/38 text messages were sent via study mobile phone - 8/38 changed numbers or lost mobile phones during the study - Intervention participants were sent an average of 12 goal- related texts and 12 log reminder texts during the first semester - Few adolescents turned in log sheets in either group |
| Mameli et al. 2018 | 1. Goals and planning  1.1 Goal setting (behaviour)  1.4 Action planning  1.5 Review of behavioural goal(s)  4. Shaping knowledge  4.1 Instructions on how to perform the behaviour  8. Repetition and substitution  8.3 Habit formation  9. Comparison of outcomes  9.1 Credible source | NR | - NR |
| Nguyen et al. 2012 | 1. Goals and planning  1.1 Goal setting (behaviour)  4. Shaping knowledge  4.1 Instructions on how to perform the behaviour  8. Repetition and substitution  8.3 Habit formation  9. Comparison of outcomes  9.1 Credible source | NR | - 10/49 opted for text message only contact, 26/49 opted for text message and email contact and 13/49 opted for email only contact - 592 e-contact messages with 130 replies - Messages about healthy eating and booster session reinforcement had highest reply rates (42% and 34%) - Self-esteem/stress messages had the lowest response rates (4%) - 17/39 found text messages somewhat helpful, 10/39 very helpful and 7 unhelpful |
| Patrick et al. 2013 | 1. Goals and planning  1.1 Goal setting (behaviour)  1.2 Problem solving  1.4 Action planning  1.5 Review of behavioural goal(s)  4. Shaping knowledge  4.1 Instructions on how to perform the behaviour  7. Associations  7.1 Prompts/cues  8. Repetition and substitution  8.3 Habit formation  9. Comparison of outcomes  9.1 Credible source | NR | NR |
| **Abbreviations:** F, female; M, male; NR, not reported. | | | |
