# Supplementary material for: Effectiveness of Text Message Interventions for Weight Management in Adolescents: Systematic Review
Source: JMIR Mhealth Uhealth. 2020 May 26;8(5):e15849. doi: 10.2196/15849 (PMC7284408; doi:10.2196/15849)
Supplement: Multimedia Appendix 6 [file mhealth_v8i5e15849_app6.docx]

| **Table S13.** The Cochrane Collaboration’s tool for assessing risk of bias in individual included studies (n=8) | | | | | | | | | | | | |
| --- | --- | --- | --- | --- | --- | --- | --- | --- | --- | --- | --- | --- |
| **Author, Year, Citation** | **Selection bias** | | | | **Performance bias** | | **Detection bias** | | **Attrition bias** | | **Reporting bias** | |
|  | **Random sequence generation** | | **Allocation concealment** | | **Blinding of participants and personnel** | | **Blinding of outcome assessment** | | **Incomplete outcome data** | | **Selective reporting** | |
|  | **Judgment** | **Supporting evidence** | **Judgment** | **Supporting evidence** | **Judgment** | **Supporting evidence** | **Judgment** | **Supporting evidence** | **Judgment** | **Supporting evidence** | **Judgment** | **Supporting evidence** |
| Abraham et al. 2015 | Low risk | Random sequence generated by a computer program in variable block sizes of 6 | Low risk | Central allocation by a paediatrician who was not involved in recruitment, enrolment or follow-up | Unclear risk | Insufficient information to permit judgement | Unclear risk | Insufficient information to permit judgement | Low risk | No missing outcome data | Low risk | Study protocol and trial registration available and all pre-specified outcomes reported |
| Bagherniya et al. 2018 | Unclear risk | Insufficient information to permit judgement | Unclear risk | Insufficient information to permit judgement | Unclear risk | Insufficient information to permit judgement | Low risk | Blinding of outcome assessment | High risk | Higher dropout in intervention  compared with control; no intention-to-treat analysis | Low risk | Trial registration available and all pre-specified outcomes reported |
| Chen et al. 2017 | Low risk | Random sequence generated using a randomisation table | Unclear risk | Insufficient information to permit judgement | Unclear risk | Insufficient information to permit judgement | Unclear risk | Insufficient information to permit judgement | Low risk | No missing outcome data at 3-mo, missing outcome data balanced across groups at 6-mo | Low risk | Trial registration available and all pre-specified outcomes reported |
| Jensen et al. 2019 | Low risk | Random sequence generated using a random number generator | Low risk | Performed by a research assistant not involved in clinical service provision | Unclear risk | Insufficient information to permit judgement | Unclear risk | Insufficient information to permit judgement | Low risk | Higher dropout in the intervention compared to control condition; intention-to-treat analysis | Unclear risk | Insufficient information to permit judgement |
| Love-Osborne et al. 2016 | Unclear risk | Insufficient information to permit judgement | Unclear risk | Insufficient information to permit judgement | Unclear risk | Insufficient information to permit judgement | Unclear risk | Insufficient information to permit judgement | Low risk | Missing outcome data balanced across groups at 6-8-mos with similar reasons for missing data; no intention-to-treat analysis | Unclear risk | Insufficient information to permit judgement |
| Mameli et al. 2018 | Low risk | Random sequence generated by a computer program | Unclear risk | Insufficient information to permit judgement | High risk | No blinding of participants and personnel | High risk | No blinding of outcome assessment | Low risk | Missing outcome data balanced across groups with similar reasons for missing data; no intention-to-treat analysis | Unclear risk | Insufficient information to permit judgement |
| Nguyen et al. 2012 | Low risk | Random sequence generated by a computer program | Low risk | Sequentially numbered opaque envelopes | Unclear risk | Insufficient information to permit judgement | Unclear risk | Insufficient information to permit judgement | Low risk | Missing outcome data balanced across groups with similar reasons for missing data; intention-to-treat analysis | Low risk | Study protocol available and all pre-specified outcomes reported |
| Patrick et al. 2013 | Unclear risk | Insufficient information to permit judgement | Unclear risk | Insufficient information to permit judgement | Unclear risk | Insufficient information to permit judgement | Unclear risk | Insufficient information to permit judgement | Low risk | Missing outcome data balanced across groups with similar reasons for missing data; intention-to-treat analysis | Unclear risk | Insufficient information to permit judgement |
